# Evaluation of the Involvement of Heme Oxygenase-1 Expression in Discoid Lupus Erythematosus Lesions

**DOI:** 10.3390/antiox12071352

**Published:** 2023-06-27

**Authors:** Paolo Fagone, Eliana Piombino, Katia Mangano, Rocco De Pasquale, Ferdinando Nicoletti, Rosario Caltabiano

**Affiliations:** 1 Department of Biomedical and Biotechnological Sciences, University of Catania, 95123 Catania, Italykmangano@unict.it (K.M.); 2 Department of Medical, Surgical and Advanced Technologies “G.F. Ingrassia”, University of Catania, Via Santa Sofia, 87, 95123 Catania, Italy; elianapiombino@hotmail.it (E.P.); rocaltab@unict.it (R.C.); 3 Department of General Surgery and Medical-Surgical Specialties, University of Catania, 95123 Catania, Italy; r.depasquale@unict.it

**Keywords:** discoid lupus erythematosus, *HMOX1*, inflammation, skin diseases

## Abstract

Discoid lupus erythematosus (DLE) is a chronic autoimmune disease that primarily affects the skin, causing red, scaly patches that may be disfiguring and can cause permanent scarring. This study aimed to investigate the potential clinical and therapeutic applications of heme oxygenase-1 (HMOX1) in the context of DLE. Immunohistochemical staining and bioinformatics analysis were performed on skin biopsy samples from DLE patients to examine the levels of HMOX1 and to correlate with markers of inflammation. Our study revealed a negative correlation between HMOX1 levels and the inflammatory status of DLE lesions, as well as an inverse correlation between HMOX1 levels and the infiltration of M1 macrophages and activated mastocytes. These findings suggest that HMOX1 plays a crucial role in the regulation of inflammation in DLE and could be a potential therapeutic target and biomarker for DLE.

## 1. Introduction

DLE (discoid lupus erythematosus) is a chronic autoimmune skin disease that primarily affects the skin and mucous membranes, with minimal involvement of other internal organs. Approximately 5% of DLE patients will develop systemic lupus erythematosus (SLE) during their lifetimes. It is more commonly diagnosed in people between the ages of 20 and 40, but it can also occur in children and in patients over 70 years of age in 3% of cases. DLE is more prevalent in women than in men and in individuals with dark skin. Its incidence is 4–5 cases per 1000 people. The exact cause of DLE is not well understood, but both genetic and environmental factors are thought to play a role. The pathogenesis of DLE involves the action of B lymphocytes and T cytotoxic lymphocytes, which attack the skin keratinocytes, leading to a hydropic degeneration of the basal layer [1].

The diagnosis of DLE is based on clinical features, but only a skin biopsy can provide a definitive diagnosis through histology [2,3]. Laboratory examinations can show anemia, leukopenia, or thrombocytopenia in 30% of patients, elevated ESR in 20%, and a positive rheumatoid factor in 17%. The presence of antinuclear antibodies (ANAs) is found in 35% of patients, and at immunofluorescence, they exhibit a dotted appearance with low titers. Anti-DNA antibodies are low, and if present, they predispose to DLE or disseminated DLE, with 10% of patients presenting anti-Ro antibodies without any effect on the clinical phenotype. Serum complement levels may occasionally be reduced. The presence of anti-Ro/SSA or anti-La/SSB antibodies is associated with photosensitivity to UVB rays, leading to apoptosis in keratinocytes and the translocation of antigens (Ro, La, and Sm) to the cell membrane.

Histological examinations reveal follicular hyperkeratosis, epidermal atrophy, modest basal layer vacuolar degeneration, thickened basal membranes, perivascular fibrinoid degeneration, dense perivascular and periannexial lymphocytic inflammatory infiltrates, basophilic collagen degeneration, fragmented elastic fibers, and ectasia of the superficial vessels. Direct immunofluorescence (DIF) is useful in detecting deposits of IgG immunoglobulins and complements components along the dermal–epidermal junction (lupus band test). DIF is positive in 80–90% of patients, but the lupus band is not considered pathognomonic [2].

The course of DLE is chronic with periodic relapses, typically in spring. Complete remission is observed in 50% of patients years later. Relapses are common due to increased stress, trauma, or sun exposure, particularly in the spring. The risk of DLE evolving into systemic lupus erythematosus (SLE) is 6.5%, especially in the case of disseminated DLE, particularly if there is renal or joint involvement. Lesions that do not respond to drug treatment can persist and result in atrophy and scarring alopecia. In 3% of patients, squamous cell carcinoma or, less commonly, basal cell carcinoma can develop into a scarring discoid lesion [2].

There is no permanent cure for DLE, but without treatment, the rash often worsens. The aim of treatment is to prevent lesion progression and limit scarring. The therapy consists of various types of treatments, including local and intralesional glucocorticoids, calcineurin inhibitors, and synthetic antimalarials. The use of topical corticosteroids is advised, but some authors believe they may not be effective and cause persistent erythroses. Intralesional glucocorticoids are recommended for hyperkeratotic lesions. Calcineurin inhibitors are effective topical immunosuppressants without the risk of atrophy or suppression of the hypothalamic–pituitary system. Synthetic antimalarials, such as hydroxychloroquine and chloroquine, have a response latency of three months and can produce clinical improvements in 80% of patients but should not be used together due to the risk of maculopathy. Other drugs, such as thalidomide and dapsone, can also be used but have side effects such as peripheral neuropathy and hemolysis, respectively. Prednisone can be used in acute cases but is not recommended for long-term therapy. Azathioprine and methotrexate are used for severe and refractory cases, respectively [2].

Heme oxygenase-1 (HMOX1), also known as heat shock protein 32, encoded by the *HMOX1* gene, is an enzyme involved in the breakdown of heme. This enzyme catabolizes heme, a highly reactive molecule, into bilirubin, iron, and carbon monoxide (CO). Each of these products is believed to play a unique role in mediating the anti-inflammatory and antioxidant effects of HMOX1. Bilirubin, the end product of heme degradation by HMOX1, has been shown to possess potent antioxidant properties. CO, on the other hand, is a gaseous signaling molecule that can interact with various metal-containing enzymes, including the transcription factors and enzymes involved in inflammation and oxidative stress.

There is growing evidence to support the anti-inflammatory and antioxidant effects of HMOX1 and its products in various disease models. We previously observed that peripheral blood mononuclear cells (PBMCs) from relapsing–remitting multiple sclerosis (RR MS) patients express less *HMOX1* than PBMCs from healthy controls and that *HMOX1* transcriptional levels are significantly downregulated during the acute exacerbation of the disease [4]. Additionally, the pharmacological induction of HMOX1 has been shown to be effective in reducing inflammation and oxidative stress in various animal models of diseases, such as rheumatoid arthritis, type 2 diabetes, and neurodegeneration [4,5,6,7,8,9,10]. Along the same lines, numerous preclinical studies conducted on animal models have indicated that the administration of carbon monoxide (CO) in appropriate doses could be an effective treatment for medical conditions that exhibit an abnormal inflammatory response [10]. Carbon monoxide-releasing molecules (CORMs) offer a promising alternative to CO gas in terms of both pharmaceutical feasibility and specificity of action. CORMs allow for controlled amounts of CO to be delivered to tissues and organs under appropriate conditions by binding CO covalently to transition metal carbonyls [11,12,13,14,15]. 

The aim of this study is to evaluate the expression of HMOX1 in patients with DLE and to evaluate the relationship between its expression and the extent of the inflammatory infiltrate.

## 2. Materials and Methods

### 2.1. Immunohistochemistry Analysis

#### 2.1.1. Patients

In total, 36 patients with DLE, 18 men and 18 women, with an age range between 30 and 50 years were recruited for the present study at the Dermatology of A.O.U. San Marco, Catania. Biopsies were performed after a six-month drug wash-out period. Demographic data of these patients are available in Caltabiano et al., 2021 [16].

The tissue samples were obtained via incisional skin biopsies and then fixed in 10% buffered formalin for 12 h and embedded in paraffin. Next, 3–4 μm sections were stained in hematoxylin and eosin and evaluated histologically to confirm the diagnosis of DLE. The morphological diagnosis was made on the basis of established criteria. The histological criteria were follicular plugging, the presence of interfollicular epidermal interface dermatitis, thickened basement membrane, and the presence of a lymphocytic infiltrate in both the superficial and deep dermis and the subcutis.

In cutaneous biopsy DLE samples of patients affected by DLE, we assessed the extent of the inflammatory infiltrate using hematoxylin and eosin staining and quantified it according to a semiquantitative score (0; 1+; 2+; 3+). As control, HMOX1 was also evaluated in unaffected skin samples from the same patients.

#### 2.1.2. Immunohistochemistry

Immunohistochemical analyses were performed using the standard streptavidin–biotin labeling technique using an LSAB kit (Dako, Glostrup, Denmark) with appropriate positive and negative controls. Sections derived from paraffin-embedded specimens were deparaffinized in xylene for 15 min, rehydrated, and treated with 3% H_2_O_2_ for 10 min to block endogenous peroxidase activity, followed by extensive rinsing in double-distilled water and further rinsing for 15 min in 0.01 M phosphate-buffered saline (PBS) at pH 7.4. Deparaffinized sections were incubated with anti-HMOX1 monoclonal antibody (MA1-112 ThermoFisher Scientific, Waltman, MA, USA) and diluted 1:500 in PBS (Sigma, Milan, Italy), and the immunoreaction was visualized using a DAB substrate kit (Vector Laboratories, CA, USA). Accordingly, all sections were pretreated with citrate buffer (pH 6.0) and exposed to radiation in a microwave oven. To reduce the commonly seen nonspecific immunoreactivity due to endogenous biotin, sections were pretreated with 10 mg/mL of ovalbumin in PBS, followed by 0.2% biotin in PBS, each for 15 min at room temperature. Bound antibodies were revealed via incubation with 3,3-diaminobenzidine (Sigma-Aldrich, St. Louis, MO, USA) in 0.01% H_2_O_2_ for 5 min at room temperature. The sections were lightly counterstained with Gill’s hematoxylin (Histolab Products AB, Göteborg, Sweden) and mounted in GVA mountant (Zymed Laboratories, San Francisco, CA, USA). Immunohistochemistry positive staining was defined as the presence of brown chromogen. Negative controls involved the omission of the primary antibody. The sections were examined using a Zeiss Axioplan light microscope (Carl Zeiss, Oberkochen, Germany) and photographed using the Aperio Scanscope CS2 system.

#### 2.1.3. Evaluation of Immunohistochemistry

Immunostained slides were separately evaluated by two pathologists (RC and EP), who were blinded to patient identity, clinical status, and group identification, using a light microscope. The percentage of HMOX1 positive cells was independently evaluated by 2 investigators and scored as a percentage of the final number of 100 cells in 4 categories: <5% (0); 5–33% (+); 33–66% (++); and >66% (+++). Counting was performed at 200× magnification.

### 2.2. In Silico Analysis

#### 2.2.1. Dataset Selection

The NCBI Gene Expression Omnibus (GEO) database (http://www.ncbi.nlm.nih.gov/geo/, accessed on 8 January 2022) was used to identify transcriptomic datasets for DLE skin lesions. The GEO database was manually searched using the string “(discoid lupus erythematosus, skin) AND “Homo sapiens” [porgn: txid9606]”. Finally, the GSE81071 dataset was selected for the subsequent analyses. For the generation of this dataset, RNA was extracted from 26 formalin-fixed paraffin embedded skin biopsy samples and then analyzed using the Affymetrix Human Gene ST 2.1 arrays. Batch correction was performed using the Combat function. A total of 20.410 genes passed the quality control. Details of the experimental design can be retrieved in the relative publications [17,18,19,20,21].

#### 2.2.2. Deconvolution Analysis

In order to evaluate the proportions of the different infiltrating immune cell subsets in DLE lesions, we performed a computational deconvolution analysis, using the web-based utility, CIBERSORTx [22]. The CIBERSORTx algorithm was executed with batch correction, quantile normalization, and 500 permutations in relative mode. The results of the deconvolution were considered significant if the CIBERSORTx *p*-value was less than 0.05, indicating the overall fit of the results across all cell subsets.

#### 2.2.3. Enrichment Analysis

To identify genes functionally associated with *HMOX1*, correlation analysis was performed using the nonparametric Spearman’s test. Spearman’s r ≥ |0.7|and FDR < 0.05 were chosen as thresholds for the selection of the genes of interest. Functional enrichment analysis was performed using the molecular complex detection (MCODE) algorithm [23]. Transcription factor prediction was performed using the GSEA enrichment score analysis [24]. Both MCODE and transcription factor analysis were implemented in the web-based utility, Metascape [25].

#### 2.2.4. *HMOX1* Perturbation Gene Signature

In order to characterize the biological processes regulated by *HMOX1*, we constructed an *HMOX1* perturbation consensus signature by meta-analyzing shRNA knockdown expression datasets from the Library of Integrated Network-based Cellular Signatures (LINCS) L1000 perturbation data [26]. The Stouffer’s method for the meta-analysis of the z-scores was employed [27], and genes with a z score > |1.96| were considered to define the consensus metasignature. Gene ontology was performed using the web-based utility, Metascape [25].

### 2.3. Statistical Analysis

For the correlation between the IHC levels of HMOX1 and the infiltration score, the Spearman’s test was used. A *p*-value < 0.05 was used as threshold for statistical significance. Hierarchical clustering was constructed using one-minus Spearman rank correlation as metric and average linkage. To identify differentially expressed genes (DEGs) in DLE lesions compared to normal skin using the GSE81071 dataset, we employed the LIMMA (linear model for microarray analysis) test. We considered genes with an adjusted (Benjamini–Hochberg-corrected) *p*-value (FDR: false discovery rate) < 0.05 and a log_2_ (fold change) > |1| as DEGs.

## 3. Results

### 3.1. Immunohistochemistry Study

In the DLE lesions, we first evaluated the extent of the inflammatory infiltrate and quantified it according to a semiquantitative score (0; 1+; 2+; and 3+). We then evaluated the expression of HMOX1 in three compartments: the epidermis, cutaneous appendages, and inflammatory infiltrate, assigning a score (0; 1+; 2+; and 3+). As indicated in Table 1, HMOX1 expression was diffused and intense (2+; 3+) in 26/36 of the cases examined (Table 1).

**Figure 1:**
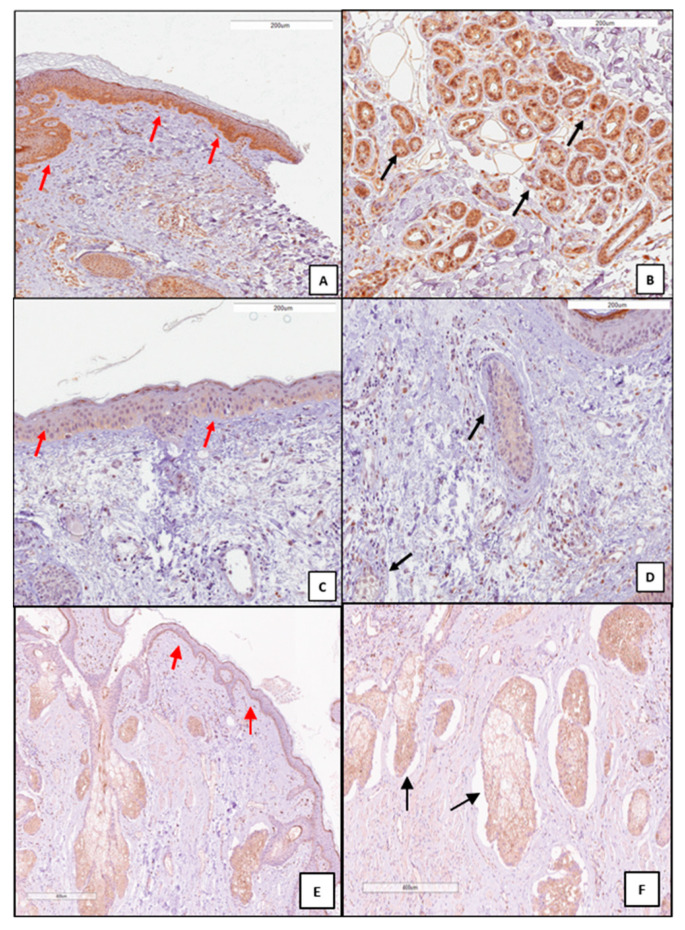
Immunohistochemistry analysis of HMOX1 expression in DLE skin biopsies. Representative microphotographs are shown. In the presence of a low inflammatory score (**A**,**B**) (0; 1+), HMOX1 high expression (2+; 3+) was observed in the epidermis (red arrow) and appendages (black arrow). In the presence of a high inflammatory score (**C**,**D**) (2+; 3+), HMOX1 low expression (0; 1+) was observed in the epidermis (red arrow) and appendages (black arrow). Immunohistochemistry analyses of HMOX1 expression in normal skin biopsies are shown in (**E**,**F**). High levels of HMOX1 can be detected at the basal layer of the epidermis (red arrow) and in the cutaneous appendages (black arrow). Immunohistochemistry positive staining was defined as the presence of brown chromogen detection within the cytoplasm.

In the samples in which the inflammatory score was low (1+), the expression of HMOX1 in the epidermis and in the appendages was found to be inversely proportional to the inflammatory score and consequently high (2+; 3+) (Figure 1A,B). In the samples in which the inflammatory score was high (2+; 3+), the expression of HMOX1 in the appendages and in the epidermis was low (1+) (Figure 1C,D).

Analysis of the expression of HMOX1 in the unaffected skin biopsies of DLE patients revealed that HMOX1 can be detected at low levels at the basal layer of the epidermis and in the cutaneous appendages (eccrine glands and sebocytes) (Figure 1E,F).

**Figure 2:**
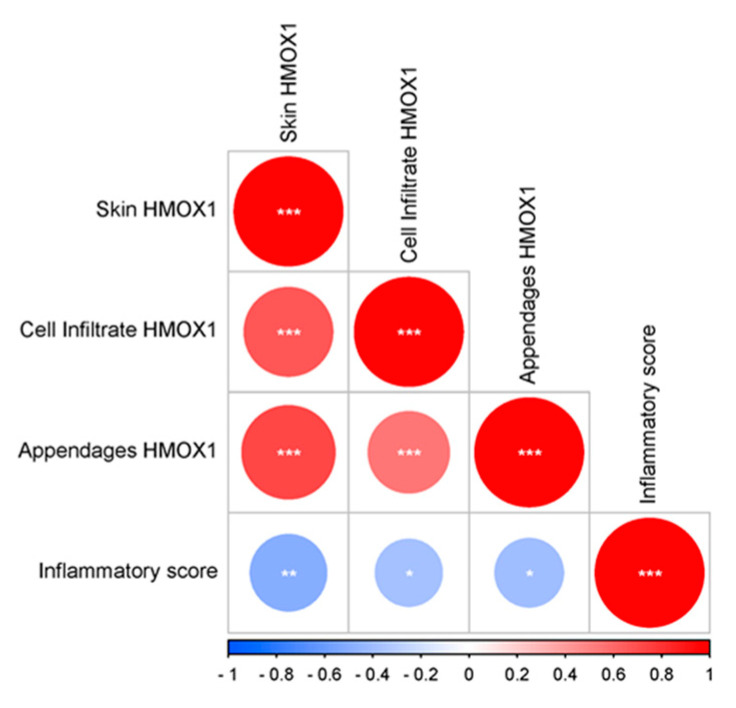
HMOX1 expression and inflammation in DLE lesion. Correlation plot for HMOX1 expression and the inflammatory infiltrate. The Spearman’s correlation test was applied for the assessment of the statistical significance. * *p* < 0.05; ** *p* < 0.01; and *** *p* < 0.001.

Overall, a significant negative correlation was observed between the inflammatory score and the levels of HMOX1 in DLE lesions (Figure 2).

### 3.2. In Silico Analysis

#### 3.2.1. Deconvolution Analysis

**Figure 3:**
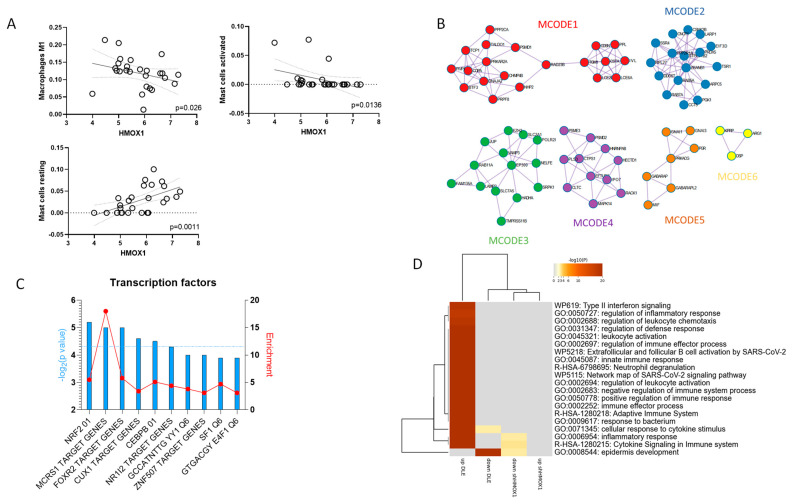
Functional analysis of HMOX1. (**A**) Correlation analysis between *HMOX1* expression levels and the proportions of immune infiltrate determined using deconvolution analysis, based on the GSE81071 dataset. (**B**) MCODE clustering analysis for the genes positively correlated to *HMOX1* in GSE81071 dataset. (**C**) Analysis of transcription factors putatively involved in the regulation of genes positively correlated with *HMOX1*, as determined using the GSE81071 dataset. (**D**) Hierarchical clustering for the top biological processes enriched by the genes regulated upon *HMOX1* inhibition from the L1000 database and for the differentially expressed genes characterizing DLE lesions, determined using the GSE81071 dataset.

To validate the IHC observations, we retrieved the publicly available microarray dataset, GSE81071, and we characterized the relative proportions of infiltrating immune cells, using the in silico deconvolution method implemented in the CIBERSORTx software. This method allowed us to determine the relative percentage of infiltration of 22 different hematopoietic cell populations. The analysis of correlation between the expression levels of *HMOX1* and the proportions of immune infiltrate revealed a significant inverse correlation for M1 macrophages (*p* = 0.026) and activated mastocytes (*p* = 0.0136), while a significant positive correlation was observed between the *HMOX1* levels and resting mast cells (*p* = 0.0011) (Figure 3A) (Table S1).

#### 3.2.2. Functional Prediction Analysis

In order to obtain insights into the biological meaning of *HMOX1* in DLE skin lesions, we first identified the genes significantly correlated, both positively and negatively, to *HMOX1* expression, and then we performed network and MCODE analyses. In total, 221 genes were positively correlated, while 50 genes were negatively correlated to HMOX1 (Spearman’s r ≥ |0.7| and FDR < 0.05) (File S1).

MCODE clustering analysis revealed a significant enrichment for several terms, in particular related to the formation of the cornified envelope (MCODE1), the response of Mtb to phagocytosis (MCODE2), the activation of anterior HOX genes in hindbrain development (MCODE3), the regulation of mRNA stability by proteins that bind AU-rich elements (MCODE4), and the GABAergic synapse (MCODE5) (Figure 3B) (Table 2) (File S1).

Analysis of the transcription factors putatively involved in the regulation of the genes positively correlated to *HMOX1* identified NRF2 (FDR = 0.004); MCRS1 (FDR = 0.006); FOXR2 (FDR = 0.006); CUX1 (FDR = 0.01); and CEBPB (FDR = 0.013) as the top five most significant transcription factors involved in their modulation (Figure 3C) (File S1).

Next, we aimed to investigate whether DLE lesions were associated with altered biological processes (BPs) controlled by HMOX1. To achieve this, we utilized a consensus *HMOX1* target gene signature obtained by meta-analyzing data from the L1000 Database. The database provided the gene expression signature of seven cell lines (A375, A549, HA1E, HEPG2, HT29, MCF7, and PC3) treated with a specific *HMOX1* shRNA for either 96 or 144 h (File S2).

We then conducted gene ontology analysis for the genes regulated upon *HMOX1* inhibition and for the differentially expressed genes (DEGs) characterizing the DLE lesions obtained from the GSE81071 dataset. As illustrated in Figure 3D, we found commonly enriched BPs in both DLE lesions and upon HMOX1 inhibition. These BPs include epidermis development (GO:0008544), multicellular organismal homeostasis (GO:0048871), and cell–cell adhesion (GO:0098609) (File S2).

## 4. Discussion

Our study aimed to investigate the potential clinical and therapeutic applications of this protein, particularly in the context of its role in inflammatory skin diseases, such as DLE. Although there have been studies on HMOX1 expression in the skin [28,29] and linking the over-expression of HMOX1 to dermatological disorders, such as psoriasis and atopic dermatitis, and to systemic diseases, such as systemic lupus erythematosus, no studies have yet investigated HMOX1 in DLE.

We have shown for the first time that reduced levels of HMOX1 negatively correlates with the inflammatory status of DLE lesions. The IHC data were strengthened by the bioinformatic analysis that also revealed the inverse correlation between *HMOX1* levels and the infiltration of M1 macrophages and the presence of activated mastocytes in DLE lesions. Our observation is in line with the recent evidence showing that myeloid cells, including monocytes and macrophages, are important drivers of cutaneous inflammation in CLE disease, contrary to the long-standing association of T cells with the disease. In mice, monocytes were found to infiltrate the irradiated skin and to produce type I IFN following UV-mediated injury. Upon irradiation, the increase in CSF-1 production by keratinocytes mediates the infiltration of macrophages into the skin and the development of CLE-like lesions [30,31,32]. Immunohistochemical studies have shown that FasL-expressing CD68+ macrophages are present near the hair follicles in lesional CLE and that CD68+ macrophages can be identified in CLE lesions following photoprovocation [33]. Moreover, the depletion of macrophages abrogated skin disease in murine lupus, supporting the hypothesis that macrophages play a proinflammatory role in CLE via the production of IFNα and IFNβ [34].

Mast cells have also been associated with the development of CLE, with CLE skin having higher numbers of mast cells than healthy skin. Mast cells are chemo-attracted to UV-exposed skin by the IL-15 and CCL5 produced by keratinocytes and, in turn, produce activated MMPs, which correlate with disease activity [32,33].

The findings from our study have brought to light the crucial role of HMOX1 in the context of DLE and the need for further research to fully understand its properties and potential applications. DLE is a chronic and inflammatory skin disease that can have a significant impact on the patient’s quality of life. The discovery of HMOX1 as a potential therapeutic target and biomarker opens up new diagnostic and treatment options for DLE patients.

Interestingly, we observed that *HMOX1* expression correlates with levels of the macrophage migration inhibitory factor (*MIF*).

MIF is a cytokine that plays a role in both innate and adaptive immunity and has several proinflammatory properties [35,36,37,38,39,40,41,42,43,44]. MIF is a highly evolutionary conserved protein. It actively participates in various stages of the inflammatory response, either directly on cells or by amplifying the effects of other stimuli. MIF has been found to counteract the inhibitory effects of glucocorticoids on the production of several cytokines, and it can also restore T cell proliferation by enhancing the production of cytokines. MIF can activate the extracellular signal-regulated kinase (ERK) pathway and has chemokine-like properties, binding to the CXCR2 and CXCR4 chemokine receptors. Additionally, MIF can inhibit downstream pathways by binding intracellular proteins, such as JAB1. Recent studies have suggested that MIF is involved in the activation of the inflammasome [35,36,37,38,39,40,41,42,43,44]. In a previous study, we found high levels of MIF in both the basal layer of the epidermis and the cutaneous appendage, including the eccrine glands and sebocytes in normal skin, and in DLE lesions, higher expression levels of MIF were associated with less severe inflammation [16]. This finding was also supported by an analysis of MIF expression levels in a public microarray dataset, which showed a similar trend toward reduced *MIF* expression in the skin of DLE patients, as well as a negative correlation between the expression of the MIF homologue, *DDT*, and inflammatory infiltrates [16]. The underlying mechanism behind the protective effects of endogenous MIF and its homologue DDT in negatively correlating with inflammation in DLE skin remains to be fully investigated. It is noteworthy to mention that MIF expression can be significantly upregulated by growth factors, including transforming growth factor-β and platelet-derived growth factor. Moreover, MIF has been found to inhibit the tumor suppressor gene, p53. Based on these observations, it is plausible to speculate that MIF, by counteracting p53 activity, may stimulate epidermal cell growth and facilitate damage repair in DLE lesions. However, it is currently unclear whether the inverse correlation between MIF and inflammation is merely an epiphenomenon associated with wound-healing processes following acute inflammatory phases or if MIF indeed plays a regulatory role in skin inflammation.

The recent evidence suggests the involvement of the MIF/CD74 cell signaling pathway in wound healing after injury, which adds further interest to the potential role of MIF in regulating inflammation. It is important to note that MIF is primarily produced by Th2 cells rather than Th1 cells, indicating its potential role in the resolution phase of the disease. The apparent protective effects of endogenous MIF and DDT in DLE place this disease among the pathologies that could potentially benefit from either the endogenous or exogenous administration of MIF and DDT. This has been demonstrated in preclinical settings in amyotrophic lateral sclerosis, ischemic cardio-protection, and acute kidney injury [45].

Overall, the combined data of these two studies suggest that MIF and HMOX1 play a protective role in regulating homeostasis and inflammation in the skin and provide new insights into the etiopathogenesis of DLE. While the observed correlation between MIF, HMOX1, and the inflammatory score in DLE lesions is an interesting finding, it is important to note that correlation does not imply causation. We should be cautious in drawing any causal relationship between these variables, and further studies are needed to investigate the biological mechanisms underlying the observed correlation. A functional study will be necessary to identify whether and how MIF and HMOX1 are functionally related and to clarify their roles in the regulation of inflammation in DLE. This could help us to better understand the biological mechanisms underlying the observed correlation and to identify new targets for the treatment of DLE.

It is also interesting to note that the transcription factor NRF2 was significantly enriched for the genes positively correlated to *HMOX1*. The NRF2-KEAP1-HMOX1 pathway is a cellular defense mechanism that plays a critical role in protection against oxidative stress and inflammation. This pathway is activated in response to various stimuli, including reactive oxygen species (ROS), heavy metals, and xenobiotics. Once activated, it leads to the transcriptional upregulation of antioxidant and detoxifying genes, including HMOX1 [46,47]

This is important in light of the fact that numerous NRF2-activating compounds have been identified and investigated in preclinical studies, although the use of these substances for therapeutic purposes in clinical settings is still limited. To date, DMF (Tecfidera) is the only NRF2-inducing agent approved by the United States Food and Drug Administration for the treatment of multiple sclerosis [48]. In Europe, Fumaderm, a drug made up of DMF with monoethyl fumarate salts, is used to treat plaque psoriasis [49]. Two research studies have shown the beneficial effects of fumaric acid esters in effectively treating DLE [49,50] and severe chilblain lesions [50]. These data, along with our findings, establish NRF2 as a leading target for the possible treatment of DLE. To fully understand the role of NRF2 in DLE and to explore its potential as a therapeutic target, it is essential to conduct further studies specifically focused on the expression and modulation of NRF2 in individuals with DLE. These studies should be aimed at investigating the levels of NRF2 and its downstream targets in affected skin lesions, as well as evaluating the efficacy and safety of NRF2-inducing agents in DLE patients.

Another important aspect of our study was the establishment of a consensus *HMOX1*-related gene signature, which was then compared with the differentially expressed genes characterizing the DLE lesions. This comparative analysis revealed a number of commonly enriched biological processes that shed light on the potential pathogenetic effect of HMOX1 alteration in DLE. The identification of commonly enriched biological processes is of particular importance as it highlights key pathways and molecular mechanisms that are potentially influenced by HMOX1 in the context of DLE. These findings provide a deeper understanding of how HMOX1 dysregulation may contribute to the development and progression of DLE, paving the way for future investigations and potentially guiding the development of targeted therapeutic approaches. The data presented here highlight the need for further studies aimed at understanding the role of HMOX1 and identifying the genetic and clinical factors that may contribute to its anti-inflammatory properties. Our study provides new diagnostic and therapeutic avenues in the context of DLE, particularly in terms of using the local expression of HMOX1 in skin lesions as a biomarker. In larger patient populations, it will be interesting to determine if a lack of HMOX1 expression in skin lesions is associated with a poorer response to treatment. Additionally, exploring genetic variations of HMOX1 in DLE is also of interest.

From a pharmacological perspective, with the advancement of specific HMOX1 inducers and CO-releasing molecules [5,14,51,52,53,54], it is important to assess the safety and effectiveness of these agents in DLE patients, both through systemic and local administration. In the future, such treatments may provide a new approach to managing DLE and other inflammatory skin diseases.

While our study has provided novel insights into the relationship between *HMOX1* expression, immune cell populations, and the biological processes underlying DLE, it is essential to acknowledge several limitations that should be taken into consideration when interpreting our findings. Firstly, our study made use of a combination of immunohistochemistry (IHC) and bioinformatics analyses. While IHC allowed us to directly assess HMOX1 expression and establish a negative correlation with the inflammatory score in DLE patients, it is important to recognize that IHC provides a snapshot of protein expression in specific tissue samples. The limited sample size and the potential variability within the patient population may impact the generalizability of these findings.

Secondly, our bioinformatic analyses heavily relied on a publicly available dataset, and the lack of detailed clinical information and metadata associated with these datasets could potentially limit the accuracy of the results. Additionally, our study focused on correlations and associations rather than establishing causality. Therefore, further experimental studies are needed to elucidate the functional relevance and underlying mechanisms. Functional studies, including in vitro and in vivo experiments, would provide a more comprehensive understanding of the role of HMOX1 in modulating inflammation and immune responses in DLE.

Furthermore, our study did not extensively explore potential confounding factors or comorbidities that could influence the observed associations. DLE is a complex disease influenced by various factors, including medication use, disease duration, and individual variations. These factors may introduce additional complexities and could impact the observed correlations. Future studies incorporating larger and more diverse cohorts with comprehensive clinical information would allow for a more thorough analysis of these confounding factors.

In conclusion, our data provide new insights into the potential therapeutic applications of HMOX1 in the context of DLE. Further research is needed to fully understand the role of HMOX1 in this disease and to explore the potential of HMOX1-based treatments. Our study highlights the importance of considering the local expression of HMOX1 in skin lesions as a diagnostic tool and therapeutic target in DLE. With the advancements in specific HMOX1 inducers and CO-releasing molecules, the presented results establish a proof-of-concept for the development of novel treatments for DLE and other inflammatory skin diseases.

## Figures and Tables

**Table 1:** Results from the IHC study of HMOX1 in DLE patients.

Patient	Skin	Inflammatory Infiltrate	Annexes	Inflammatory Score
**16/3436**	1	2	2	3
**15/161**	2	3	1	2
**15/1175**	1	1	1	3
**15/5303**	1	1	1	1
**15/5218**	1	1	1	2
**153162**	1	1	1	1
**15/4589**	1	1	1	2
**15/4494**	1	1	1	2
**14/4788**	3	3	3	2
**16/3949**	3	3	3	1
**V 14/1412**	2	1	2	3
**V 14/1812**	1	2	1	3
**V 14/1967**	3	3	3	2
**V 14/2544**	2	3	3	1
**V 14/3529**	2	2	2	1
**V 14/3962**	2	2	1	1
**V 14/3979**	3	3	3	1
**V 15/2561**	1	1	2	2
**V 16/2768**	2	2	1	2
**V 15/2561**	1	1	2	2
**V 16/2768**	2	2	1	2
**V 16/2820**	3	3	2	1
**V 16/6534**	1	1	1	3
**V 13/6726**	1	2	1	3
**V 13/6499**	1	2	1	3
**V 14/211**	2	2	2	2
**V 14/3691**	1	2	1	2
**V 14/4134**	1	1	1	2
**V 14/4804**	1	1	1	3
**V 16/7906**	2	2	1	3
**V 14/5895**	1	2	1	2
**V 14/7815**	0	0	0	3
**V 15/1041**	2	1	2	3
**V 15/4192**	1	2	1	3
**V 16/2033**	2	1	1	2
**V 16/1972**	1	1	1	3
**V 16/1891**	1	2	1	3
**V 14/7513**	1	1	1	3
**V 16/7695**	1	1	1	3

**Table 2:** MCODE analysis for the genes positively correlated to HMOX1 in GSE81071 dataset.

Network	Annotation
_FINAL_SUB1_MCODE_1	R-HSA-6809371|Formation of the cornified envelope|−11.8; GO:0030216|keratinocyte differentiation|−11.5; GO:0009913|epidermal cell differentiation|−10.5
_FINAL_SUB1_MCODE_2	R-HSA-9637690|Response of Mtb to phagocytosis|−6.6; R-HSA-9635486|Infection with Mycobacterium tuberculosis|−6.4; M210|PID IL8 CXCR2 PATHWAY|−6.1
_FINAL_SUB1_MCODE_3	R-HSA-5617472|Activation of anterior HOX genes in hindbrain development during early embryogenesis|−4.7;R-HSA-5619507|Activation of HOX genes during differentiation|−4.7; GO:0009267|cellular response to starvation|−4.2
_FINAL_SUB1_MCODE_4	R-HSA-450531|Regulation of mRNA stability by proteins that bind AU-rich elements|−5.4; R-HSA-4086400|PCP/CE pathway|−5.4; GO:0042176|regulation of protein catabolic process|−5.2
_FINAL_SUB1_MCODE_5	hsa04727|GABAergic synapse|−11.4; hsa04914|Progesterone-mediated oocyte maturation|−8.4; hsa04915|Estrogen signaling pathway|−7.8

## Data Availability

Data is contained within the article and Supplementary Material.

## Supplementary Materials

The following supporting information can be downloaded at: https://www.mdpi.com/article/10.3390/antiox12071352/s1, Table S1: CIBERSORTx deconvolution results; File S1: Ontology analysis of DEGs correlating with *HMOX1* in DLE; File S2: Ontology analysis of genes regulated upon *HMOX1* inhibition.
