# Supplementary material for: Evaluation of the Involvement of Heme Oxygenase-1 Expression in Discoid Lupus Erythematosus Lesions
Source: Antioxidants (Basel). 2023 Jun 27;12(7):1352. doi: 10.3390/antiox12071352 (PMC10376595; doi:10.3390/antiox12071352)
Supplement: Supplementary file 1 [file antioxidants-12-01352-s001.zip › File S1.zip!/Enrichment_GO/ColorByCluster.pdf]

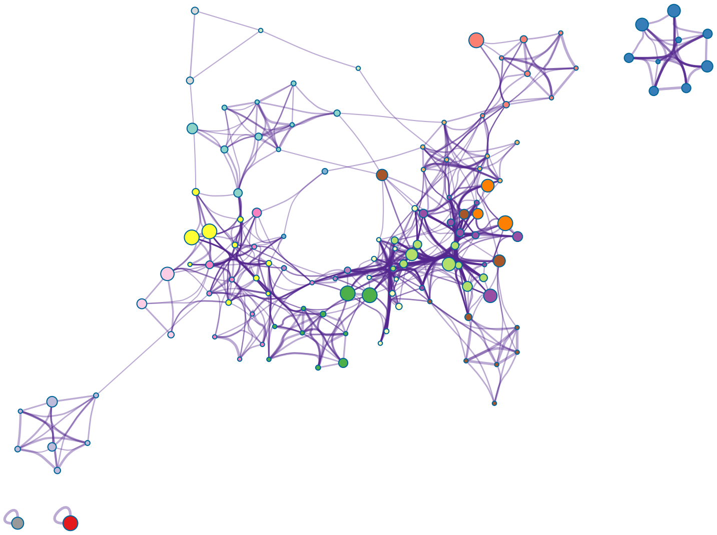

- Neutrophil degranulation
- Formation of the cornified envelope
- Signaling by Rho GTPases
- Cellular response to chemical stress
- Cellular responses to stress
- Membrane Trafficking
- Transcriptional Regulation by TP53
- Rab regulation of trafficking
- VEGFA-VEGFR2 signaling pathway
- regulation of autophagy
- PP2A A/C-striatin complex
- regulation of protein catabolic process
- Metabolism of lipids
- Intracellular trafficking proteins involved in CMT neur[illegible]
- FACT complex, UV-activated
- Cell Cycle
- intracellular protein transport
- Protein processing in endoplasmic reticulum
- PID PDGFRB PATHWAY
- Toposome

created by
http://metascape.org
