# Supplementary material for: Evaluation of the Involvement of Heme Oxygenase-1 Expression in Discoid Lupus Erythematosus Lesions
Source: Antioxidants (Basel). 2023 Jun 27;12(7):1352. doi: 10.3390/antiox12071352 (PMC10376595; doi:10.3390/antiox12071352)
Supplement: Supplementary file 1 [file antioxidants-12-01352-s001.zip › File S1.zip!/Enrichment_QC/HeatmapSelectedGO_Cell_Type_Signatures.pdf]

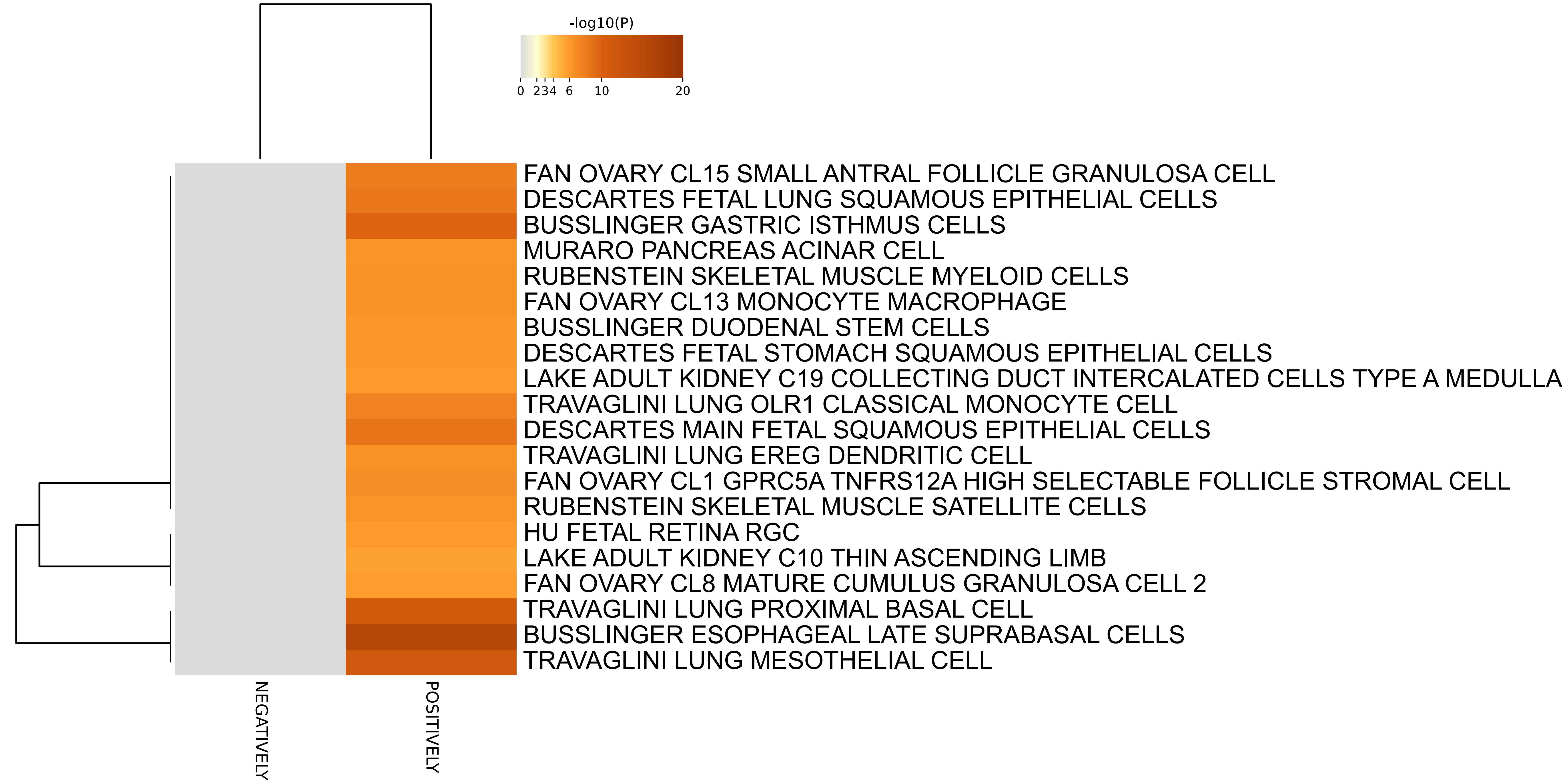
-log10(P)
0 2 3 4 6 10 20
FAN OVARY CL15 SMALL ANTRAL FOLLICLE GRANULOSA CELL
DESCARTES FETAL LUNG SQUAMOUS EPITHELIAL CELLS
BUSSLINGER GASTRIC ISTHMUS CELLS
MURARO PANCREAS ACINAR CELL
RUBENSTEIN SKELETAL MUSCLE MYELOID CELLS
FAN OVARY CL13 MONOCYTE MACROPHAGE
BUSSLINGER DUODENAL STEM CELLS
DESCARTES FETAL STOMACH SQUAMOUS EPITHELIAL CELLS
LAKE ADULT KIDNEY C19 COLLECTING DUCT INTERCALATED CELLS TYPE A MEDULLA
TRAVAGLINI LUNG OLR1 CLASSICAL MONOCYTE CELL
DESCARTES MAIN FETAL SQUAMOUS EPITHELIAL CELLS
TRAVAGLINI LUNG EREG DENDRITIC CELL
FAN OVARY CL1 GPRC5A TNFRS12A HIGH SELECTABLE FOLLICLE STROMAL CELL
RUBENSTEIN SKELETAL MUSCLE SATELLITE CELLS
HU FETAL RETINA RGC
LAKE ADULT KIDNEY C10 THIN ASCENDING LIMB
FAN OVARY CL8 MATURE CUMULUS GRANULOSA CELL 2
TRAVAGLINI LUNG PROXIMAL BASAL CELL
BUSSLINGER ESOPHAGEAL LATE SUPRABASAL CELLS
TRAVAGLINI LUNG MESOTHELIAL CELL
NEGATIVELY
POSITIVELY
