# Supplementary material for: Evaluation of the Involvement of Heme Oxygenase-1 Expression in Discoid Lupus Erythematosus Lesions
Source: Antioxidants (Basel). 2023 Jun 27;12(7):1352. doi: 10.3390/antiox12071352 (PMC10376595; doi:10.3390/antiox12071352)
Supplement: Supplementary file 1 [file antioxidants-12-01352-s001.zip › File S1.zip!/Enrichment_QC/HeatmapSelectedGO_Transcription_Factor_Targets.pdf]

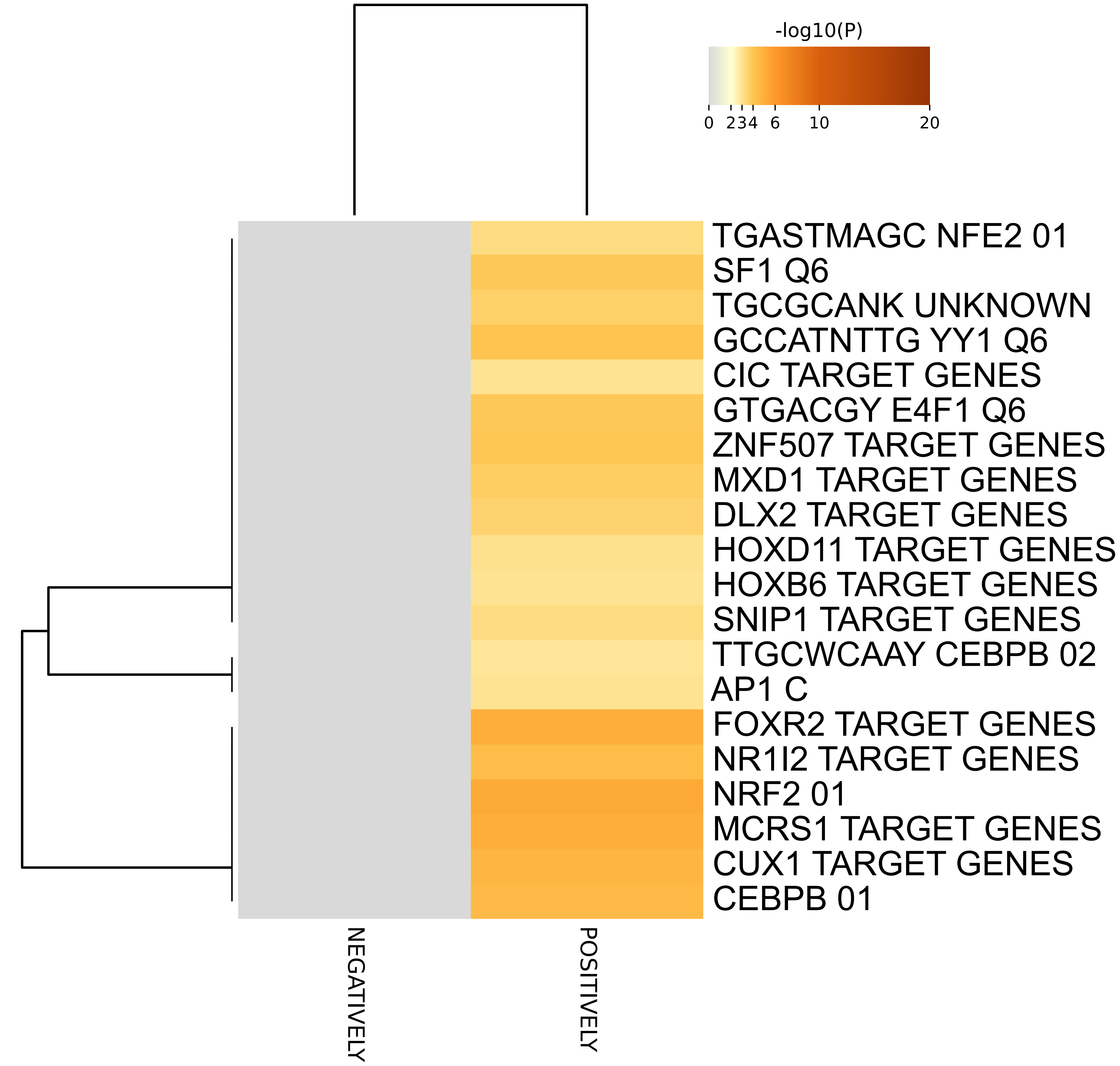
-log10(P)
0
2
3
4
6
10
20
TGASTMAGC NFE2 01
SF1 Q6
TGCGCANK UNKNOWN
GCCATNTTG YY1 Q6
CIC TARGET GENES
GTGACGY E4F1 Q6
ZNF507 TARGET GENES
MXD1 TARGET GENES
DLX2 TARGET GENES
HOXD11 TARGET GENES
HOXB6 TARGET GENES
SNIP1 TARGET GENES
TTGCWCAAY CEBPB 02
AP1 C
FOXR2 TARGET GENES
NR1I2 TARGET GENES
NRF2 01
MCRS1 TARGET GENES
CUX1 TARGET GENES
CEBPB 01
NEGATIVELY
POSITIVELY
