# Supplementary material for: Evaluation of the Involvement of Heme Oxygenase-1 Expression in Discoid Lupus Erythematosus Lesions
Source: Antioxidants (Basel). 2023 Jun 27;12(7):1352. doi: 10.3390/antiox12071352 (PMC10376595; doi:10.3390/antiox12071352)
Supplement: Supplementary file 1 [file antioxidants-12-01352-s001.zip › File S1.zip!/Enrichment_QC/HeatmapSelectedGO_TRRUST.pdf]

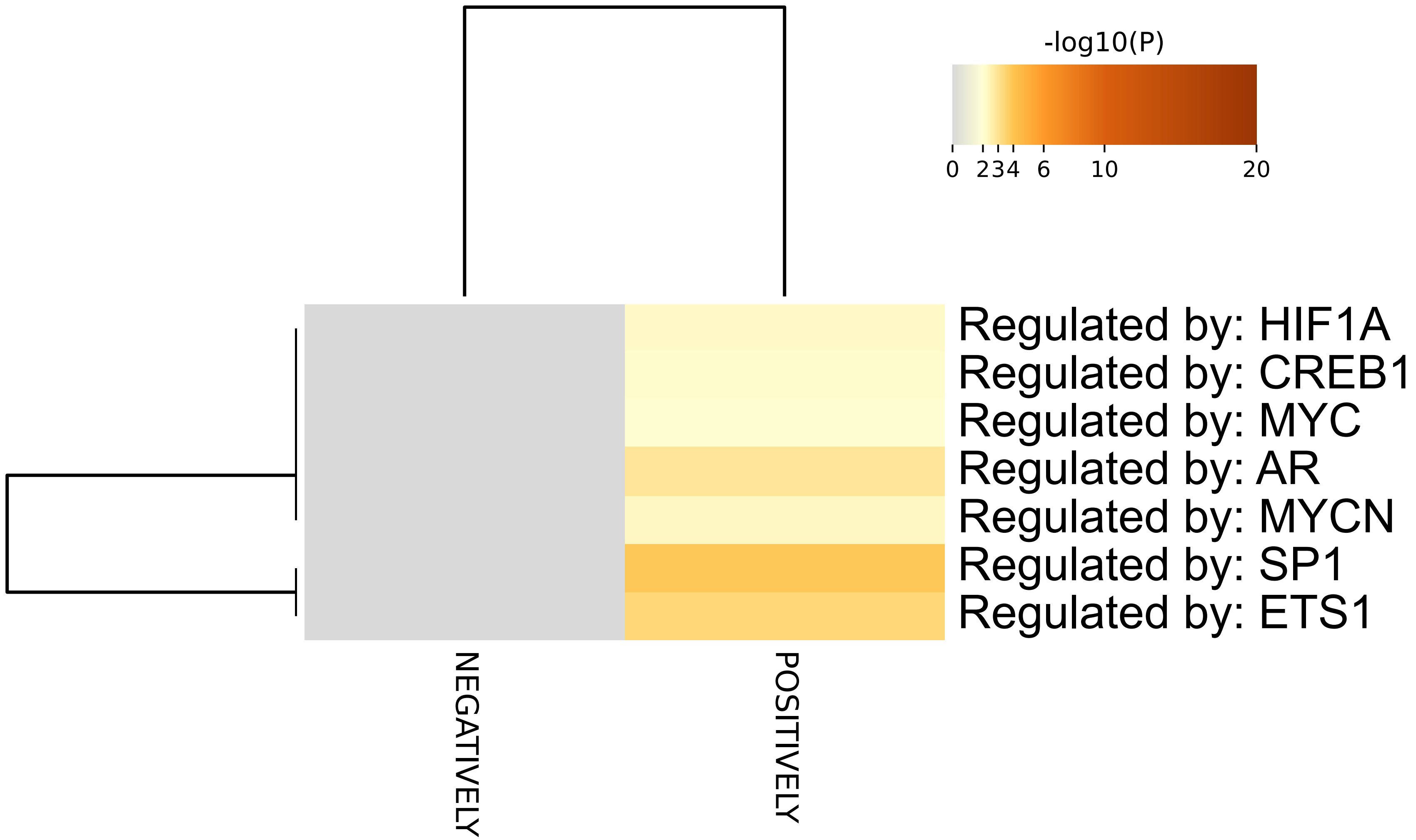
-log10(P)
0
2
3
4
6
10
20
Regulated by: HIF1A
Regulated by: CREB1
Regulated by: MYC
Regulated by: AR
Regulated by: MYCN
Regulated by: SP1
Regulated by: ETS1
NEGATIVELY
POSITIVELY
