# Supplementary material for: Evaluation of the Involvement of Heme Oxygenase-1 Expression in Discoid Lupus Erythematosus Lesions
Source: Antioxidants (Basel). 2023 Jun 27;12(7):1352. doi: 10.3390/antiox12071352 (PMC10376595; doi:10.3390/antiox12071352)
Supplement: Supplementary file 1 [file antioxidants-12-01352-s001.zip › File S1.zip!/Enrichment_QC/HeatmapSelectedGO_DisGeNET.pdf]

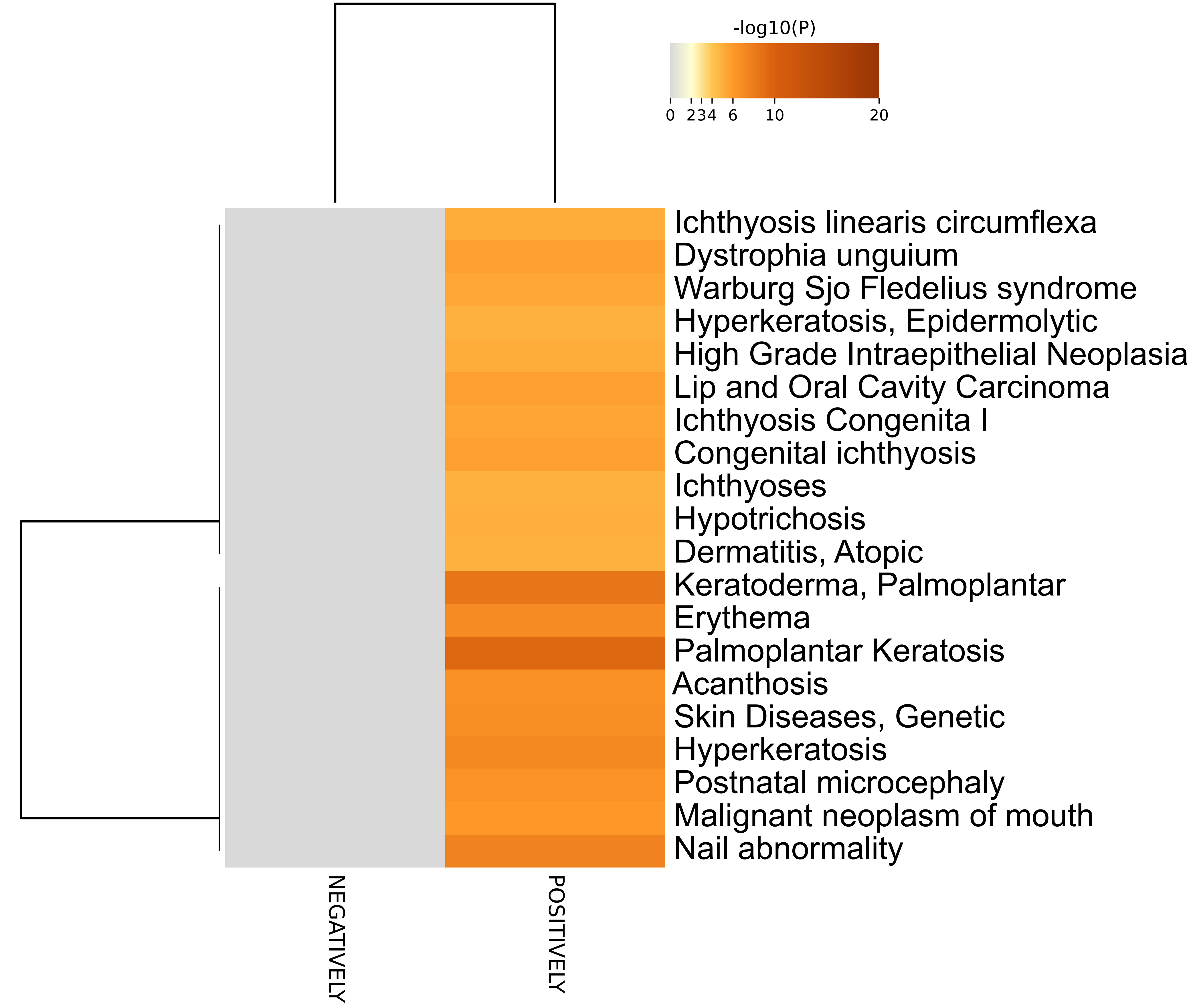
-log10(P)
0
2
3
4
6
10
20
Ichthyosis linearis circumflexa
Dystrophia unguium
Warburg Sjo Fledelius syndrome
Hyperkeratosis, Epidermolytic
High Grade Intraepithelial Neoplasia
Lip and Oral Cavity Carcinoma
Ichthyosis Congenita I
Congenital ichthyosis
Ichthyoses
Hypotrichosis
Dermatitis, Atopic
Keratoderma, Palmoplantar
Erythema
Palmoplantar Keratosis
Acanthosis
Skin Diseases, Genetic
Hyperkeratosis
Postnatal microcephaly
Malignant neoplasm of mouth
Nail abnormality
NEGATIVELY
POSITIVELY
