# Supplementary material for: Evaluation of the Involvement of Heme Oxygenase-1 Expression in Discoid Lupus Erythematosus Lesions
Source: Antioxidants (Basel). 2023 Jun 27;12(7):1352. doi: 10.3390/antiox12071352 (PMC10376595; doi:10.3390/antiox12071352)
Supplement: Supplementary file 1 [file antioxidants-12-01352-s001.zip › File S1.zip!/Enrichment_QC/HeatmapSelectedGO_COVID.pdf]

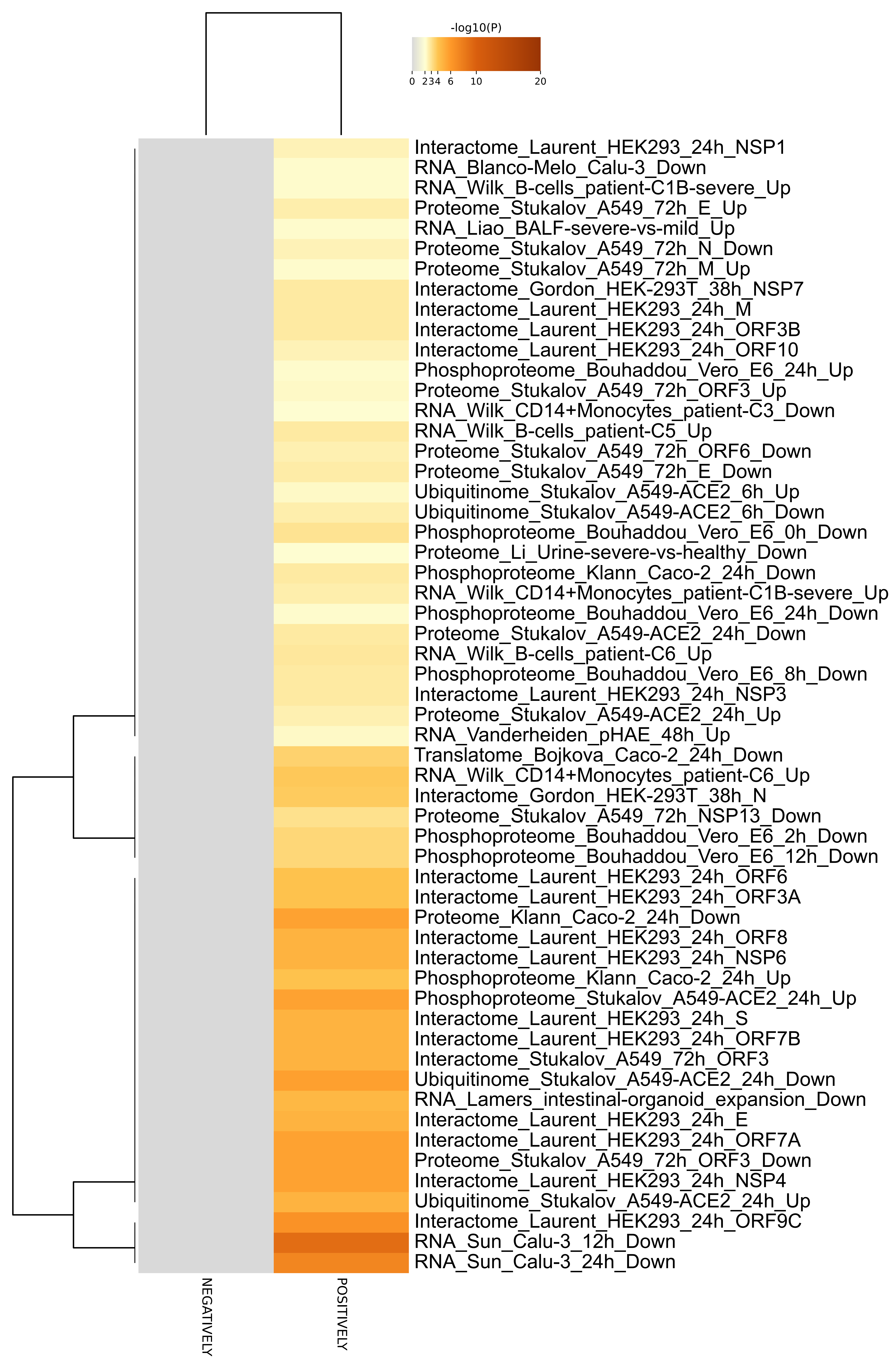

-log10(P)
0
2
3
4
6
10
20
Interactome_Laurent_HEK293_24h_NSP1
RNA_Blanco-Melo_Calu-3_Down
RNA_Wilk_B-cells_patient-C1B-severe_Up
Proteome_Stukalov_A549_72h_E_Up
RNA_Liao_BALF-severe-vs-mild_Up
Proteome_Stukalov_A549_72h_N_Down
Proteome_Stukalov_A549_72h_M_Up
Interactome_Gordon_HEK-293T_38h_NSP7
Interactome_Laurent_HEK293_24h_M
Interactome_Laurent_HEK293_24h_ORF3B
Interactome_Laurent_HEK293_24h_ORF10
Phosphoproteome_Bouhaddou_Vero_E6_24h_Up
Proteome_Stukalov_A549_72h_ORF3_Up
RNA_Wilk_CD14+Monocytes_patient-C3_Down
RNA_Wilk_B-cells_patient-C5_Up
Proteome_Stukalov_A549_72h_ORF6_Down
Proteome_Stukalov_A549_72h_E_Down
Ubiquitinome_Stukalov_A549-ACE2_6h_Up
Ubiquitinome_Stukalov_A549-ACE2_6h_Down
Phosphoproteome_Bouhaddou_Vero_E6_0h_Down
Proteome_Li_Urine-severe-vs-healthy_Down
Phosphoproteome_Klann_Caco-2_24h_Down
RNA_Wilk_CD14+Monocytes_patient-C1B-severe_Up
Phosphoproteome_Bouhaddou_Vero_E6_24h_Down
Proteome_Stukalov_A549-ACE2_24h_Down
RNA_Wilk_B-cells_patient-C6_Up
Phosphoproteome_Bouhaddou_Vero_E6_8h_Down
Interactome_Laurent_HEK293_24h_NSP3
Proteome_Stukalov_A549-ACE2_24h_Up
RNA_Vanderheiden_pHAE_48h_Up
Translatome_Bojkova_Caco-2_24h_Down
RNA_Wilk_CD14+Monocytes_patient-C6_Up
Interactome_Gordon_HEK-293T_38h_N
Proteome_Stukalov_A549_72h_NSP13_Down
Phosphoproteome_Bouhaddou_Vero_E6_2h_Down
Phosphoproteome_Bouhaddou_Vero_E6_12h_Down
Interactome_Laurent_HEK293_24h_ORF6
Interactome_Laurent_HEK293_24h_ORF3A
Proteome_Klann_Caco-2_24h_Down
Interactome_Laurent_HEK293_24h_ORF8
Interactome_Laurent_HEK293_24h_NSP6
Phosphoproteome_Klann_Caco-2_24h_Up
Phosphoproteome_Stukalov_A549-ACE2_24h_Up
Interactome_Laurent_HEK293_24h_S
Interactome_Laurent_HEK293_24h_ORF7B
Interactome_Stukalov_A549_72h_ORF3
Ubiquitinome_Stukalov_A549-ACE2_24h_Down
RNA_Lamers_intestinal-organoid_expansion_Down
Interactome_Laurent_HEK293_24h_E
Interactome_Laurent_HEK293_24h_ORF7A
Proteome_Stukalov_A549_72h_ORF3_Down
Interactome_Laurent_HEK293_24h_NSP4
Ubiquitinome_Stukalov_A549-ACE2_24h_Up
Interactome_Laurent_HEK293_24h_ORF9C
RNA_Sun_Calu-3_12h_Down
RNA_Sun_Calu-3_24h_Down
NEGATIVELY
POSITIVELY
