# Supplementary material for: Evaluation of the Involvement of Heme Oxygenase-1 Expression in Discoid Lupus Erythematosus Lesions
Source: Antioxidants (Basel). 2023 Jun 27;12(7):1352. doi: 10.3390/antiox12071352 (PMC10376595; doi:10.3390/antiox12071352)
Supplement: Supplementary file 1 [file antioxidants-12-01352-s001.zip › File S1.zip!/Enrichment_heatmap/HeatmapSelectedGO.pdf]

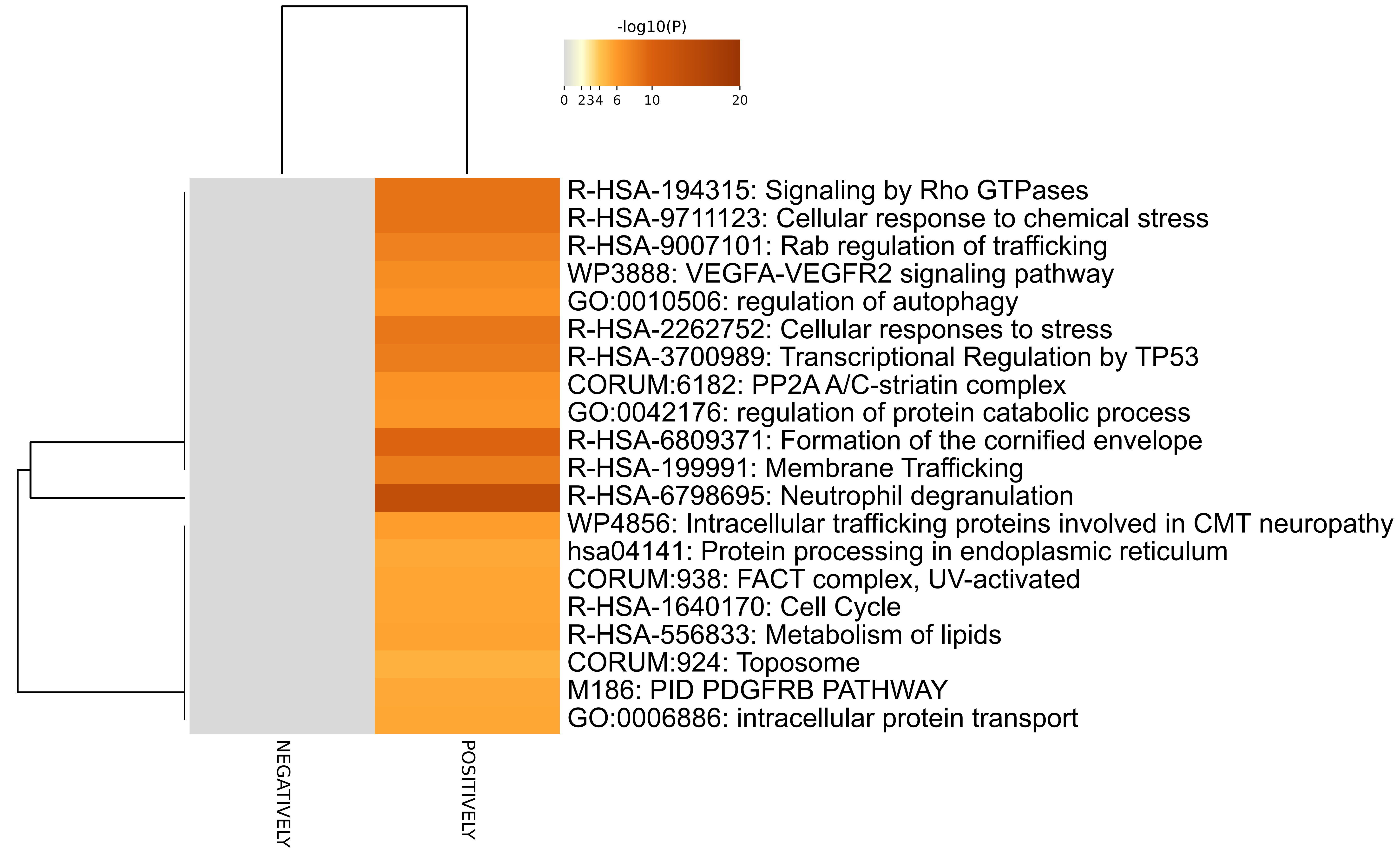
-log10(P)
0
2
3
4
6
10
20
R-HSA-194315: Signaling by Rho GTPases
R-HSA-9711123: Cellular response to chemical stress
R-HSA-9007101: Rab regulation of trafficking
WP3888: VEGFA-VEGFR2 signaling pathway
GO:0010506: regulation of autophagy
R-HSA-2262752: Cellular responses to stress
R-HSA-3700989: Transcriptional Regulation by TP53
CORUM:6182: PP2A A/C-striatin complex
GO:0042176: regulation of protein catabolic process
R-HSA-6809371: Formation of the cornified envelope
R-HSA-199991: Membrane Trafficking
R-HSA-6798695: Neutrophil degranulation
WP4856: Intracellular trafficking proteins involved in CMT neuropathy
hsa04141: Protein processing in endoplasmic reticulum
CORUM:938: FACT complex, UV-activated
R-HSA-1640170: Cell Cycle
R-HSA-556833: Metabolism of lipids
CORUM:924: Toposome
M186: PID PDGFRB PATHWAY
GO:0006886: intracellular protein transport
NEGATIVELY
POSITIVELY
