# Supplementary material for: Evaluation of the Involvement of Heme Oxygenase-1 Expression in Discoid Lupus Erythematosus Lesions
Source: Antioxidants (Basel). 2023 Jun 27;12(7):1352. doi: 10.3390/antiox12071352 (PMC10376595; doi:10.3390/antiox12071352)
Supplement: Supplementary file 1 [file antioxidants-12-01352-s001.zip › File S1.zip!/Enrichment_heatmap/HeatmapSelectedGOTop100.pdf]

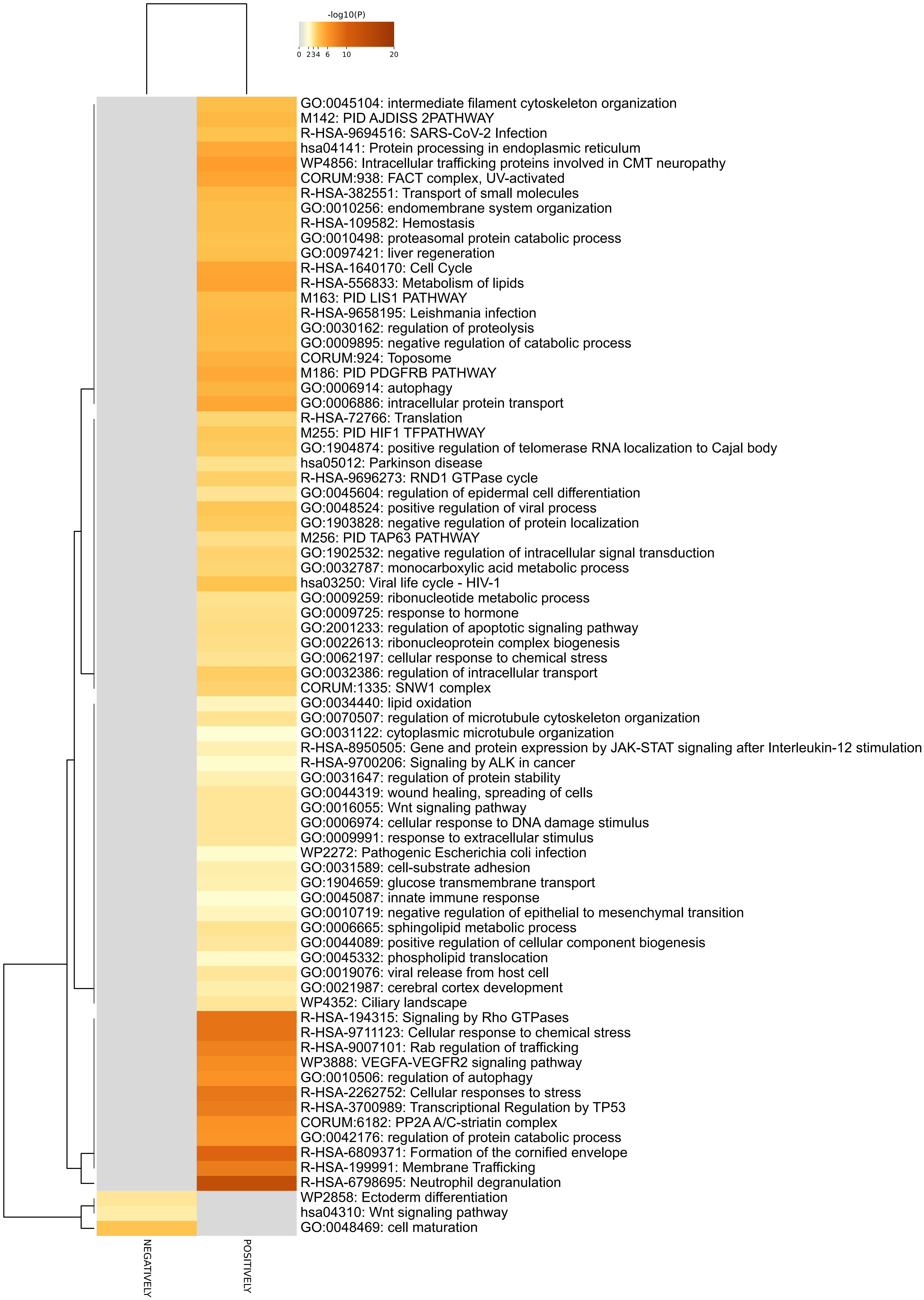

-log10(P)
0 2 3 4 6 10 20
GO:0045104: intermediate filament cytoskeleton organization
M142: PID AJDISS 2PATHWAY
R-HSA-9694516: SARS-CoV-2 Infection
hsa04141: Protein processing in endoplasmic reticulum
WP4856: Intracellular trafficking proteins involved in CMT neuropathy
CORUM:938: FACT complex, UV-activated
R-HSA-382551: Transport of small molecules
GO:0010256: endomembrane system organization
R-HSA-109582: Hemostasis
GO:0010498: proteasomal protein catabolic process
GO:0097421: liver regeneration
R-HSA-1640170: Cell Cycle
R-HSA-556833: Metabolism of lipids
M163: PID LIS1 PATHWAY
R-HSA-9658195: Leishmania infection
GO:0030162: regulation of proteolysis
GO:0009895: negative regulation of catabolic process
CORUM:924: Toposome
M186: PID PDGFRB PATHWAY
GO:0006914: autophagy
GO:0006886: intracellular protein transport
R-HSA-72766: Translation
M255: PID HIF1 TFPATHWAY
GO:1904874: positive regulation of telomerase RNA localization to Cajal body
hsa05012: Parkinson disease
R-HSA-9696273: RND1 GTPase cycle
GO:0045604: regulation of epidermal cell differentiation
GO:0048524: positive regulation of viral process
GO:1903828: negative regulation of protein localization
M256: PID TAP63 PATHWAY
GO:1902532: negative regulation of intracellular signal transduction
GO:0032787: monocarboxylic acid metabolic process
hsa03250: Viral life cycle - HIV-1
GO:0009259: ribonucleotide metabolic process
GO:0009725: response to hormone
GO:2001233: regulation of apoptotic signaling pathway
GO:0022613: ribonucleoprotein complex biogenesis
GO:0062197: cellular response to chemical stress
GO:0032386: regulation of intracellular transport
CORUM:1335: SNW1 complex
GO:0034440: lipid oxidation
GO:0070507: regulation of microtubule cytoskeleton organization
GO:0031122: cytoplasmic microtubule organization
R-HSA-8950505: Gene and protein expression by JAK-STAT signaling after Interleukin-12 stimulation
R-HSA-9700206: Signaling by ALK in cancer
GO:0031647: regulation of protein stability
GO:0044319: wound healing, spreading of cells
GO:0016055: Wnt signaling pathway
GO:0006974: cellular response to DNA damage stimulus
GO:0009991: response to extracellular stimulus
WP2272: Pathogenic Escherichia coli infection
GO:0031589: cell-substrate adhesion
GO:1904659: glucose transmembrane transport
GO:0045087: innate immune response
GO:0010719: negative regulation of epithelial to mesenchymal transition
GO:0006665: sphingolipid metabolic process
GO:0044089: positive regulation of cellular component biogenesis
GO:0045332: phospholipid translocation
GO:0019076: viral release from host cell
GO:0021987: cerebral cortex development
WP4352: Ciliary landscape
R-HSA-194315: Signaling by Rho GTPases
R-HSA-9711123: Cellular response to chemical stress
R-HSA-9007101: Rab regulation of trafficking
WP3888: VEGFA-VEGFR2 signaling pathway
GO:0010506: regulation of autophagy
R-HSA-2262752: Cellular responses to stress
R-HSA-3700989: Transcriptional Regulation by TP53
CORUM:6182: PP2A A/C-striatin complex
GO:0042176: regulation of protein catabolic process
R-HSA-6809371: Formation of the cornified envelope
R-HSA-199991: Membrane Trafficking
R-HSA-6798695: Neutrophil degranulation
WP2858: Ectoderm differentiation
hsa04310: Wnt signaling pathway
GO:0048469: cell maturation
NEGATIVELY
POSITIVELY
