# Supplementary material for: Evaluation of the Involvement of Heme Oxygenase-1 Expression in Discoid Lupus Erythematosus Lesions
Source: Antioxidants (Basel). 2023 Jun 27;12(7):1352. doi: 10.3390/antiox12071352 (PMC10376595; doi:10.3390/antiox12071352)
Supplement: Supplementary file 1 [file antioxidants-12-01352-s001.zip › File S1.zip!/Enrichment_PPI/_FINAL_MCODE_ALL_PPIColorByCounts.pdf]

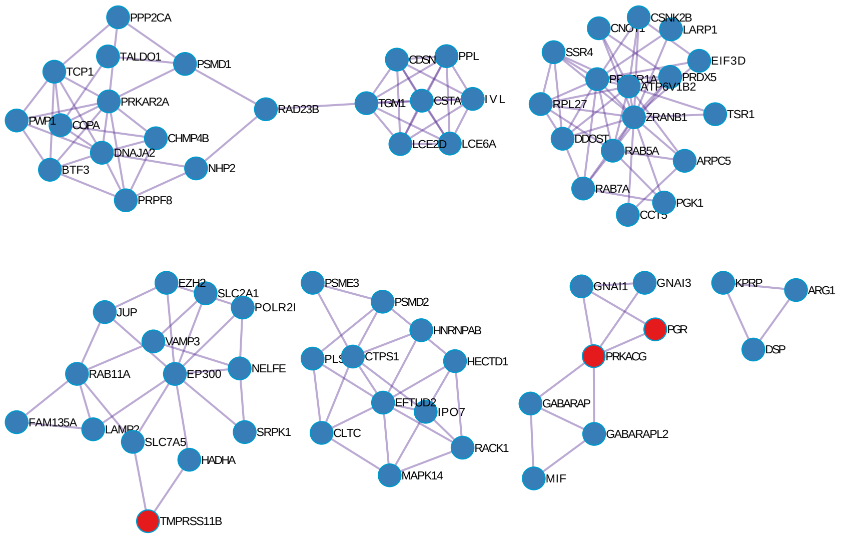
PPP2CA
TALDO1
PSMD1
TCP1
PRKAR2A
RAD23B
PWP1
COPA
CHMP4B
DNAJA2
BTF3
NHP2
PRPF8
CDSN
PPL
TGM1
CSTA
IVL
LCE2D
LCE6A
CSNK2B
LARP1
SSR4
EIF3D
PRDX5
ATP6V1B2
RPL27
ZRANB1
TSR1
DDOST
RAB5A
ARPC5
RAB7A
PGK1
EZH2
SLC2A1
POLR2I
JUP
VAMP3
RAB11A
EP300
NELFE
FAM135A
LAMP2
SLC7A5
SRPK1
HADHA
TMPRSS11B
PSME3
PSMD2
HNRNPAB
CTPS1
HECTD1
EFTUD2
IPO7
CLTC
RACK1
MAPK14
GNAI1
GNAI3
PGR
PRKACG
GABARAP
GABARAPL2
MIF
KPRP
ARG1
DSP

NEGATIVELY
POSITIVELY

created by
http://metascape.org
