# Supplementary material for: Evaluation of the Involvement of Heme Oxygenase-1 Expression in Discoid Lupus Erythematosus Lesions
Source: Antioxidants (Basel). 2023 Jun 27;12(7):1352. doi: 10.3390/antiox12071352 (PMC10376595; doi:10.3390/antiox12071352)
Supplement: Supplementary file 1 [file antioxidants-12-01352-s001.zip › File S1.zip!/Enrichment_PPI/_FINAL_MCODE_ALL_PPIColorByCluster.pdf]

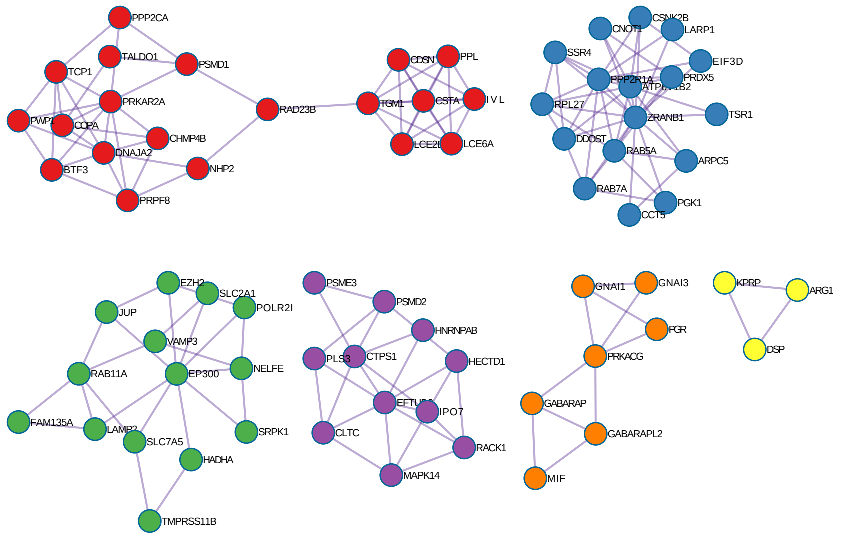
PPP2CA
TALDO1
PSMD1
TCP1
PRKAR2A
RAD23B
PWP1
COPA
CHMP4B
DNAJA2
BTF3
NHP2
PRPF8
PPL
TGM1
CSTA
IVL
LCE6A
CSNK2B
CNOT1
LARP1
SSR4
EIF3D
PPP2R1A
PRDX5
RPL27
ZRANB1
TSR1
DDOST
RAB5A
ARPC5
RAB7A
PGK1
CCT5
EZH2
SLC2A1
POLR2I
JUP
VAMP3
RAB11A
EP300
NELFE
FAM135A
LAMP2
SLC7A5
SRPK1
HADHA
TMPRSS11B
PSME3
PSMD2
HNRNPAB
PLS3
CTPS1
HECTD1
IPO7
CLTC
RACK1
MAPK14
GNAI1
GNAI3
PGR
PRKACG
GABARAP
GABARAPL2
MIF
KPRP
ARG1
DSP

MCODE1
MCODE2
MCODE3
MCODE4
MCODE5
MCODE6

created by
http://metascape.org
