# Supplementary material for: Evaluation of the Involvement of Heme Oxygenase-1 Expression in Discoid Lupus Erythematosus Lesions
Source: Antioxidants (Basel). 2023 Jun 27;12(7):1352. doi: 10.3390/antiox12071352 (PMC10376595; doi:10.3390/antiox12071352)
Supplement: Supplementary file 1 [file antioxidants-12-01352-s001.zip › File S1.zip!/Enrichment_PPI/POSITIVELY_MCODE_ALL_PPIColorByCluster.pdf]

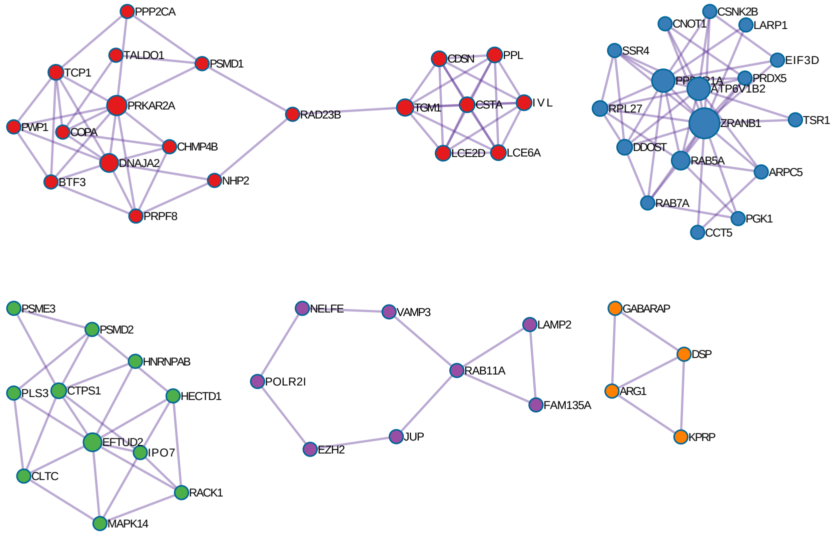
PPP2CA
TALDO1
PSMD1
TCP1
PRKAR2A
RAD23B
PWP1
COPA
CHMP4B
DNAJA2
BTF3
NHP2
PRPF8
CDSN
PPL
TGM1
CSTA
IVL
LCE2D
LCE6A
CSNK2B
CNOT1
LARP1
SSR4
EIF3D
ATP6V1B2
PRDX5
RPL27
ZRANB1
TSR1
DDOST
RAB5A
ARPC5
RAB7A
PGK1
CCT5
PSME3
PSMD2
HNRNPAB
PLS3
CTPS1
HECTD1
EFTUD2
IPO7
CLTC
RACK1
MAPK14
NELFE
VAMP3
LAMP2
POLR2I
RAB11A
FAM135A
EZH2
JUP
GABARAP
DSP
ARG1
KPRP

MCODE1
MCODE2
MCODE3
MCODE4
MCODE5

created by
http://metascape.org
