# Supplementary material for: Evaluation of the Involvement of Heme Oxygenase-1 Expression in Discoid Lupus Erythematosus Lesions
Source: Antioxidants (Basel). 2023 Jun 27;12(7):1352. doi: 10.3390/antiox12071352 (PMC10376595; doi:10.3390/antiox12071352)
Supplement: Supplementary file 1 [file antioxidants-12-01352-s001.zip › File S2.zip!/Enrichment_GO/ColorByCluster.pdf]

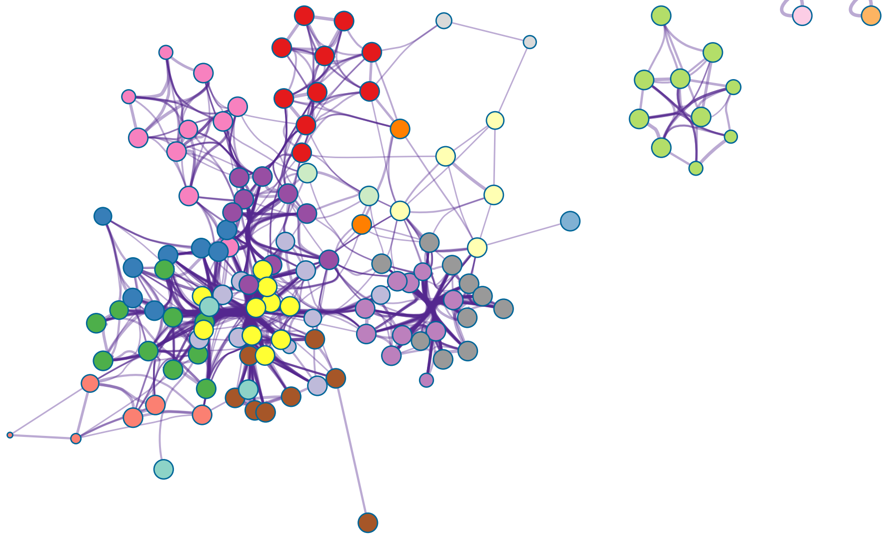

- innate immune response
- positive regulation of immune response
- leukocyte activation
- regulation of defense response
- Cytokine Signaling in Immune system
- regulation of leukocyte activation
- response to bacterium
- negative regulation of immune system process
- inflammatory response
- Adaptive Immune System
- cellular response to cytokine stimulus
- regulation of immune effector process
- immune effector process
- Network map of SARS-CoV-2 signaling pathway
- Extrafollicular and follicular B cell activation by SARS
- epidermis development
- Neutrophil degranulation
- Type II interferon signaling
- regulation of leukocyte chemotaxis
- regulation of inflammatory response

created by
http://metascape.org
