# Supplementary material for: Evaluation of the Involvement of Heme Oxygenase-1 Expression in Discoid Lupus Erythematosus Lesions
Source: Antioxidants (Basel). 2023 Jun 27;12(7):1352. doi: 10.3390/antiox12071352 (PMC10376595; doi:10.3390/antiox12071352)
Supplement: Supplementary file 1 [file antioxidants-12-01352-s001.zip › File S2.zip!/Enrichment_QC/HeatmapSelectedGO_Cell_Type_Signatures.pdf]

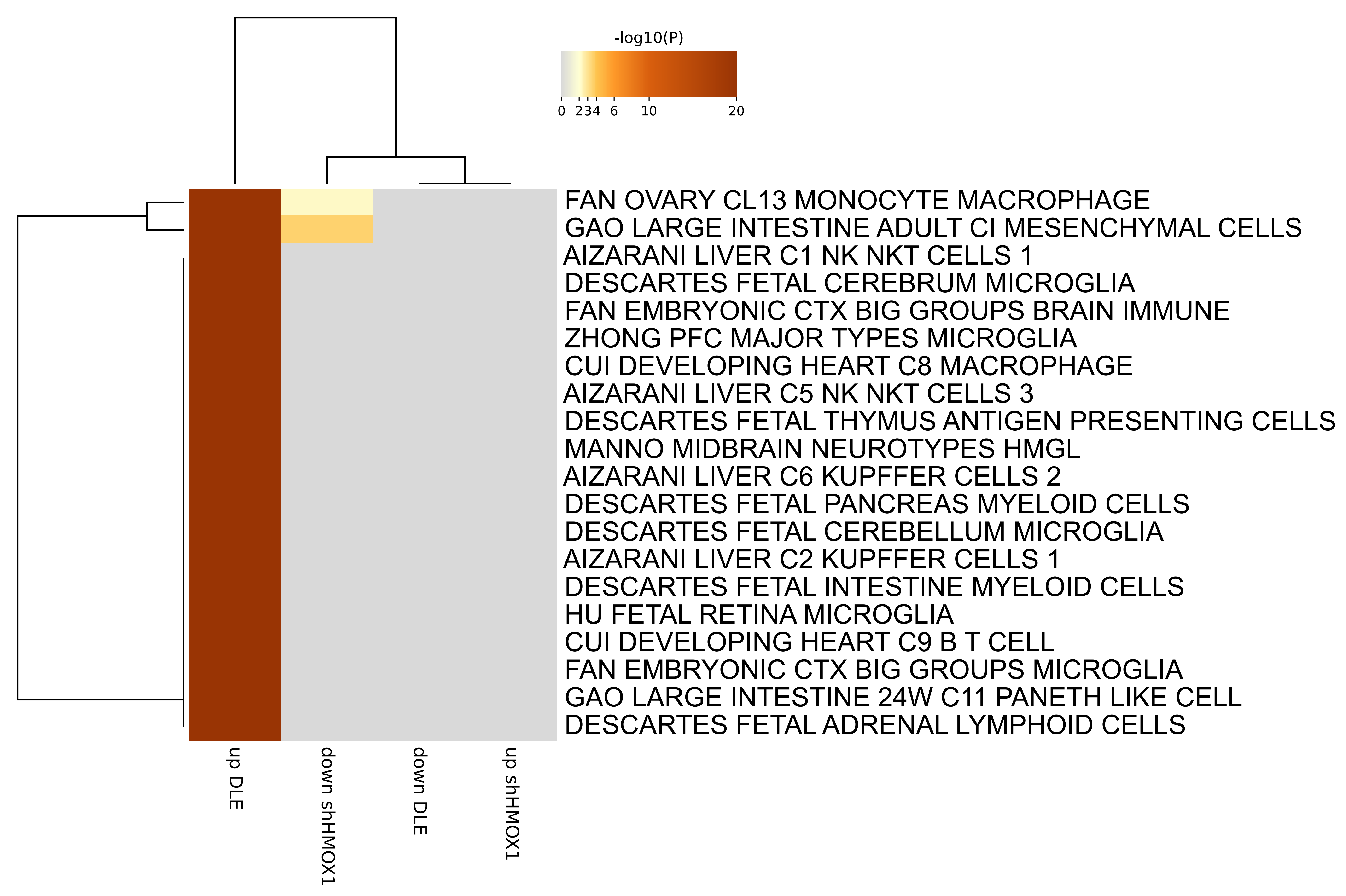
-log10(P)
0
2
3
4
6
10
20
FAN OVARY CL13 MONOCYTE MACROPHAGE
GAO LARGE INTESTINE ADULT CI MESENCHYMAL CELLS
AIZARANI LIVER C1 NK NKT CELLS 1
DESCARTES FETAL CEREBRUM MICROGLIA
FAN EMBRYONIC CTX BIG GROUPS BRAIN IMMUNE
ZHONG PFC MAJOR TYPES MICROGLIA
CUI DEVELOPING HEART C8 MACROPHAGE
AIZARANI LIVER C5 NK NKT CELLS 3
DESCARTES FETAL THYMUS ANTIGEN PRESENTING CELLS
MANNO MIDBRAIN NEUROTYPES HMGL
AIZARANI LIVER C6 KUPFFER CELLS 2
DESCARTES FETAL PANCREAS MYELOID CELLS
DESCARTES FETAL CEREBELLUM MICROGLIA
AIZARANI LIVER C2 KUPFFER CELLS 1
DESCARTES FETAL INTESTINE MYELOID CELLS
HU FETAL RETINA MICROGLIA
CUI DEVELOPING HEART C9 B T CELL
FAN EMBRYONIC CTX BIG GROUPS MICROGLIA
GAO LARGE INTESTINE 24W C11 PANETH LIKE CELL
DESCARTES FETAL ADRENAL LYMPHOID CELLS
up DLE
down shHMOX1
down DLE
up shHMOX1
