# Supplementary material for: Evaluation of the Involvement of Heme Oxygenase-1 Expression in Discoid Lupus Erythematosus Lesions
Source: Antioxidants (Basel). 2023 Jun 27;12(7):1352. doi: 10.3390/antiox12071352 (PMC10376595; doi:10.3390/antiox12071352)
Supplement: Supplementary file 1 [file antioxidants-12-01352-s001.zip › File S2.zip!/Enrichment_QC/HeatmapSelectedGO_Transcription_Factor_Targets.pdf]

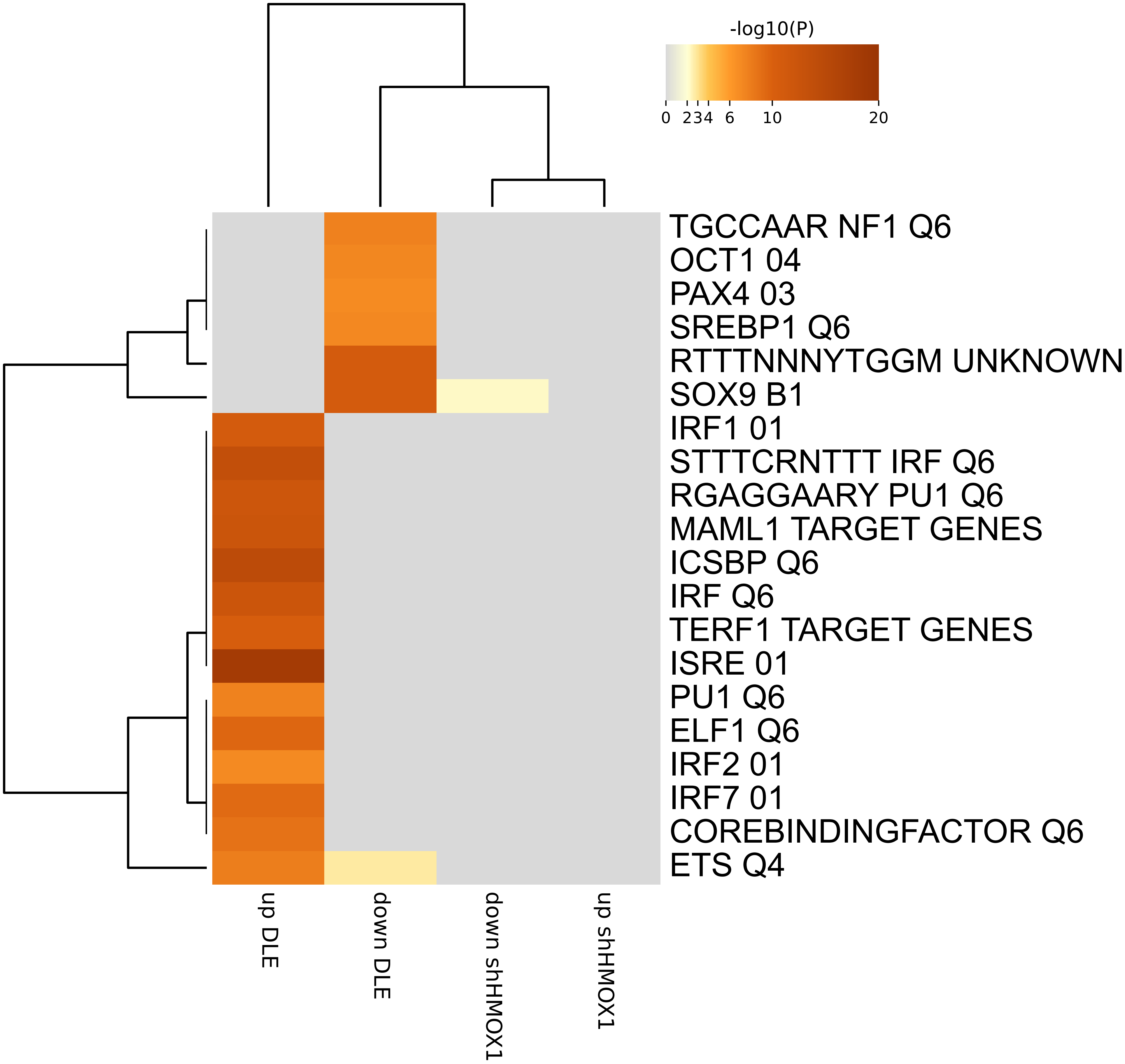
-log10(P)
0
2
3
4
6
10
20
TGCCAAR NF1 Q6
OCT1 04
PAX4 03
SREBP1 Q6
RTTTNNNYTGGM UNKNOWN
SOX9 B1
IRF1 01
STTTCRNTTT IRF Q6
RGAGGAARY PU1 Q6
MAML1 TARGET GENES
ICSBP Q6
IRF Q6
TERF1 TARGET GENES
ISRE 01
PU1 Q6
ELF1 Q6
IRF2 01
IRF7 01
COREBINDINGFACTOR Q6
ETS Q4
up DLE
down DLE
down shHMOX1
up shHMOX1
