# Supplementary material for: Evaluation of the Involvement of Heme Oxygenase-1 Expression in Discoid Lupus Erythematosus Lesions
Source: Antioxidants (Basel). 2023 Jun 27;12(7):1352. doi: 10.3390/antiox12071352 (PMC10376595; doi:10.3390/antiox12071352)
Supplement: Supplementary file 1 [file antioxidants-12-01352-s001.zip › File S2.zip!/Enrichment_QC/HeatmapSelectedGO_TRRUST.pdf]

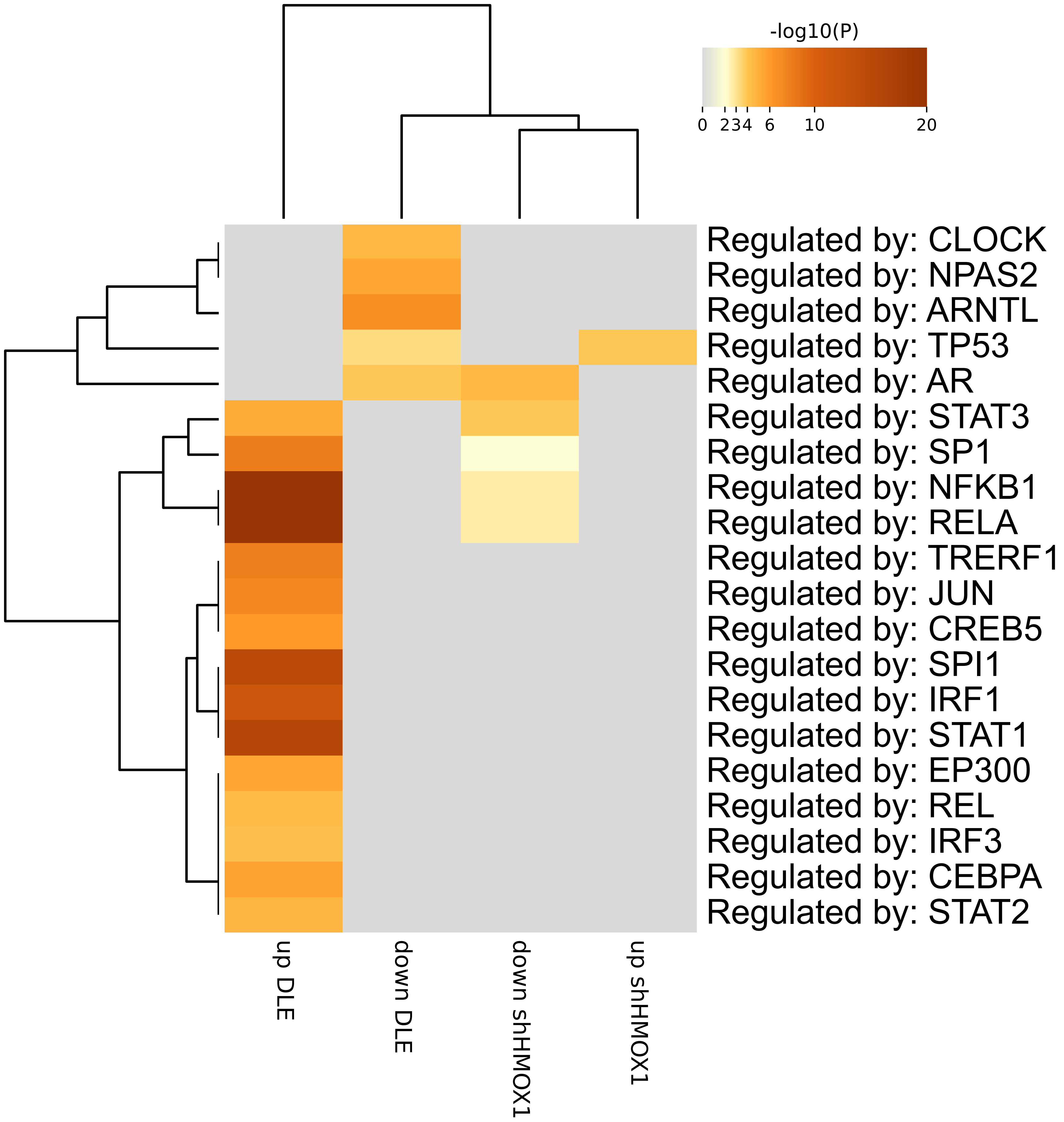

-log10(P)
0
2
3
4
6
10
20
Regulated by: CLOCK
Regulated by: NPAS2
Regulated by: ARNTL
Regulated by: TP53
Regulated by: AR
Regulated by: STAT3
Regulated by: SP1
Regulated by: NFKB1
Regulated by: RELA
Regulated by: TRERF1
Regulated by: JUN
Regulated by: CREB5
Regulated by: SPI1
Regulated by: IRF1
Regulated by: STAT1
Regulated by: EP300
Regulated by: REL
Regulated by: IRF3
Regulated by: CEBPA
Regulated by: STAT2
up DLE
down DLE
down shHMOX1
up shHMOX1
