# Supplementary material for: Evaluation of the Involvement of Heme Oxygenase-1 Expression in Discoid Lupus Erythematosus Lesions
Source: Antioxidants (Basel). 2023 Jun 27;12(7):1352. doi: 10.3390/antiox12071352 (PMC10376595; doi:10.3390/antiox12071352)
Supplement: Supplementary file 1 [file antioxidants-12-01352-s001.zip › File S2.zip!/Enrichment_QC/HeatmapSelectedGO_DisGeNET.pdf]

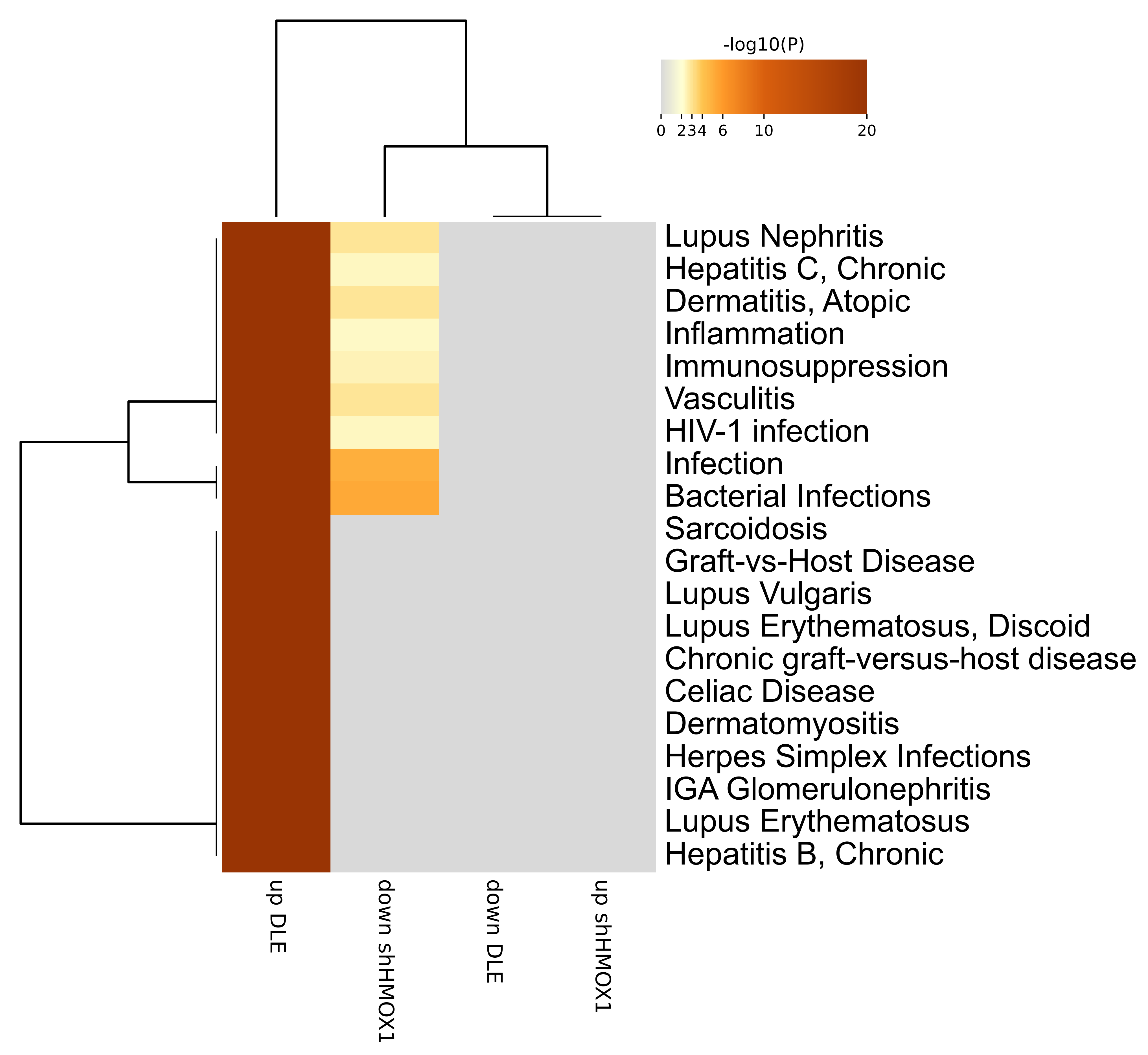
-log10(P)
0
2
3
4
6
10
20
Lupus Nephritis
Hepatitis C, Chronic
Dermatitis, Atopic
Inflammation
Immunosuppression
Vasculitis
HIV-1 infection
Infection
Bacterial Infections
Sarcoidosis
Graft-vs-Host Disease
Lupus Vulgaris
Lupus Erythematosus, Discoid
Chronic graft-versus-host disease
Celiac Disease
Dermatomyositis
Herpes Simplex Infections
IGA Glomerulonephritis
Lupus Erythematosus
Hepatitis B, Chronic
up DLE
down shHMOX1
down DLE
up shHMOX1
