# Supplementary material for: Evaluation of the Involvement of Heme Oxygenase-1 Expression in Discoid Lupus Erythematosus Lesions
Source: Antioxidants (Basel). 2023 Jun 27;12(7):1352. doi: 10.3390/antiox12071352 (PMC10376595; doi:10.3390/antiox12071352)
Supplement: Supplementary file 1 [file antioxidants-12-01352-s001.zip › File S2.zip!/Enrichment_QC/HeatmapSelectedGO_PaGenBase.pdf]

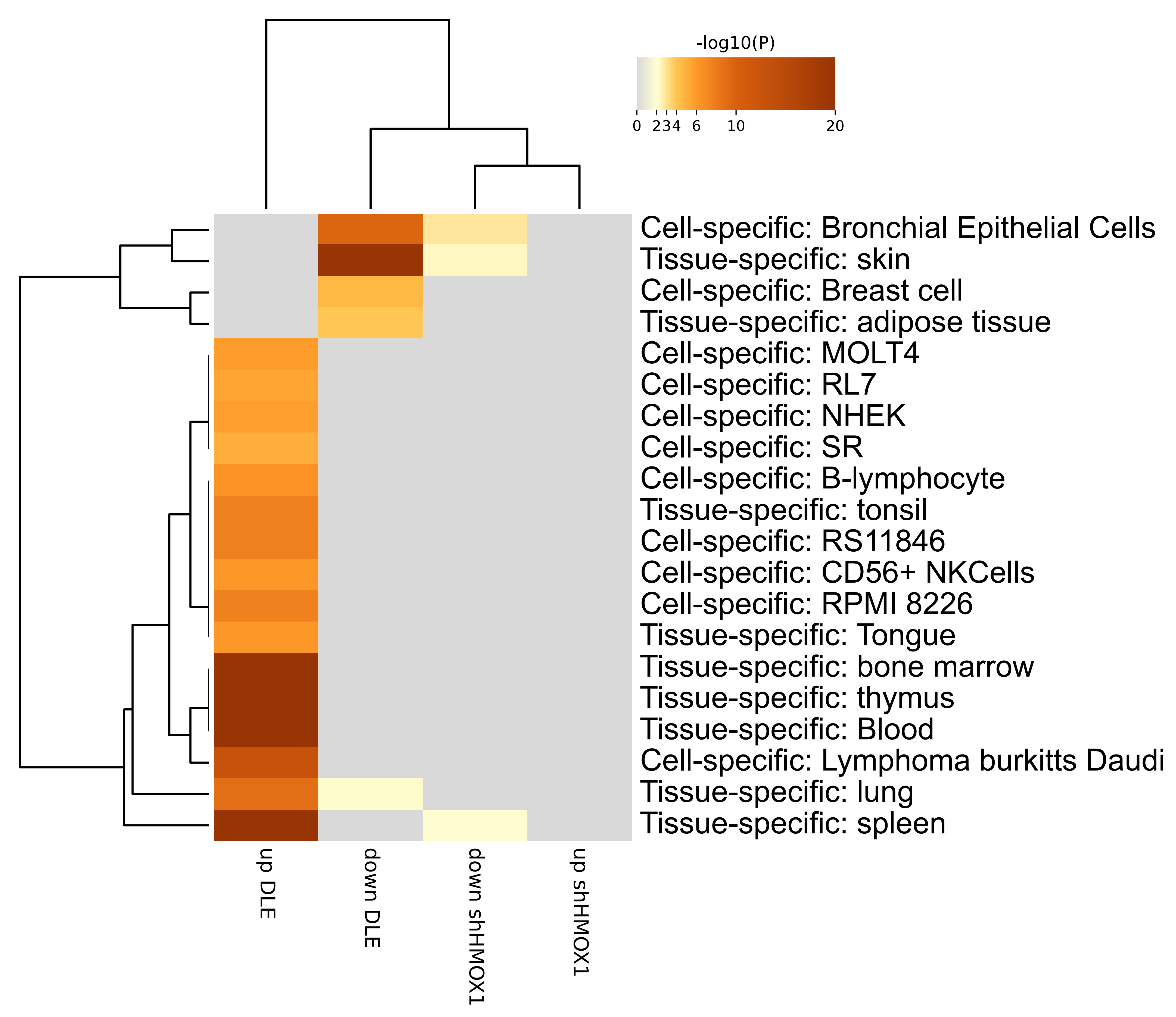
-log10(P)
0
2
3
4
6
10
20
Cell-specific: Bronchial Epithelial Cells
Tissue-specific: skin
Cell-specific: Breast cell
Tissue-specific: adipose tissue
Cell-specific: MOLT4
Cell-specific: RL7
Cell-specific: NHEK
Cell-specific: SR
Cell-specific: B-lymphocyte
Tissue-specific: tonsil
Cell-specific: RS11846
Cell-specific: CD56+ NKCells
Cell-specific: RPMI 8226
Tissue-specific: Tongue
Tissue-specific: bone marrow
Tissue-specific: thymus
Tissue-specific: Blood
Cell-specific: Lymphoma burkitts Daudi
Tissue-specific: lung
Tissue-specific: spleen
up DLE
down DLE
down shHMOX1
up shHMOX1
