# Supplementary material for: Evaluation of the Involvement of Heme Oxygenase-1 Expression in Discoid Lupus Erythematosus Lesions
Source: Antioxidants (Basel). 2023 Jun 27;12(7):1352. doi: 10.3390/antiox12071352 (PMC10376595; doi:10.3390/antiox12071352)
Supplement: Supplementary file 1 [file antioxidants-12-01352-s001.zip › File S2.zip!/Enrichment_QC/HeatmapSelectedGO_COVID.pdf]

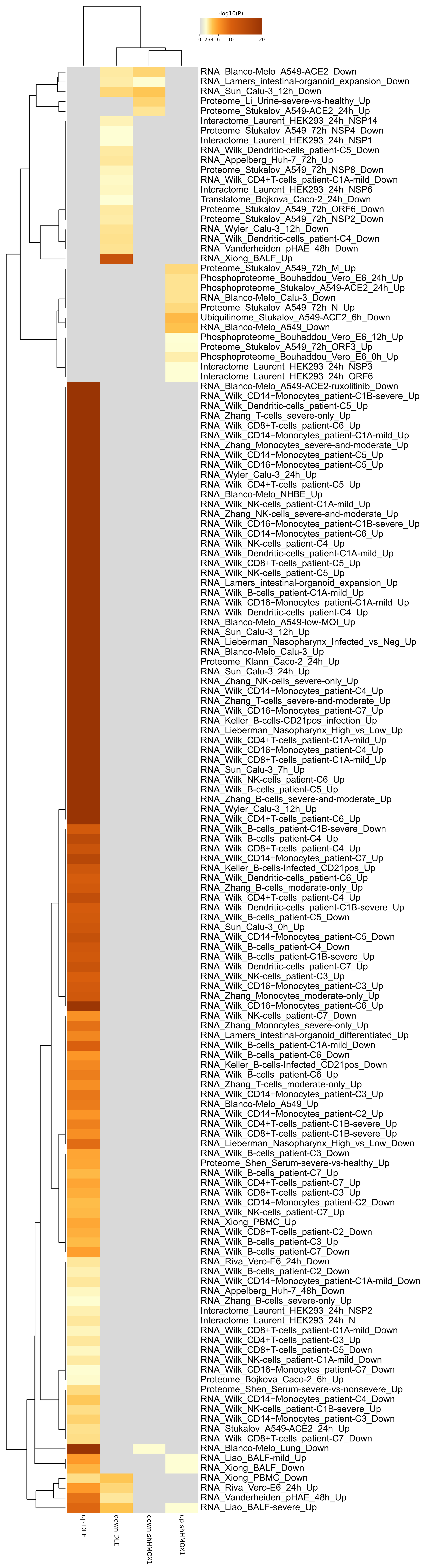

-log10(P)
0
234
6
10
20
RNA_Blanco-Melo_A549-ACE2_Down
RNA_Lamers_intestinal-organoid_expansion_Down
RNA_Sun_Calu-3_12h_Down
Proteome_Li_Urine-severe-vs-healthy_Up
Proteome_Stukalov_A549-ACE2_24h_Up
Interactome_Laurent_HEK293_24h_NSP14
Proteome_Stukalov_A549_72h_NSP4_Down
Interactome_Laurent_HEK293_24h_NSP1
RNA_Wilk_Dendritic-cells_patient-C5_Down
RNA_Appelberg_Huh-7_72h_Up
Proteome_Stukalov_A549_72h_NSP8_Down
RNA_Wilk_CD4+T-cells_patient-C1A-mild_Down
Interactome_Laurent_HEK293_24h_NSP6
Translatome_Bojkova_Caco-2_24h_Down
Proteome_Stukalov_A549_72h_ORF6_Down
Proteome_Stukalov_A549_72h_NSP2_Down
RNA_Wyler_Calu-3_12h_Down
RNA_Wilk_Dendritic-cells_patient-C4_Down
RNA_Vanderheiden_pHAE_48h_Down
RNA_Xiong_BALF_Up
Proteome_Stukalov_A549_72h_M_Up
Phosphoproteome_Bouhaddou_Vero_E6_24h_Up
Phosphoproteome_Stukalov_A549-ACE2_24h_Up
RNA_Blanco-Melo_Calu-3_Down
Proteome_Stukalov_A549_72h_N_Up
Ubiquitinome_Stukalov_A549-ACE2_6h_Down
RNA_Blanco-Melo_A549_Down
Phosphoproteome_Bouhaddou_Vero_E6_12h_Up
Proteome_Stukalov_A549_72h_ORF3_Up
Phosphoproteome_Bouhaddou_Vero_E6_0h_Up
Interactome_Laurent_HEK293_24h_NSP3
Interactome_Laurent_HEK293_24h_ORF6
RNA_Blanco-Melo_A549-ACE2-ruxolitinib_Down
RNA_Wilk_CD14+Monocytes_patient-C1B-severe_Up
RNA_Wilk_Dendritic-cells_patient-C5_Up
RNA_Zhang_T-cells_severe-only_Up
RNA_Wilk_CD8+T-cells_patient-C6_Up
RNA_Wilk_CD14+Monocytes_patient-C1A-mild_Up
RNA_Zhang_Monocytes_severe-and-moderate_Up
RNA_Wilk_CD14+Monocytes_patient-C5_Up
RNA_Wilk_CD16+Monocytes_patient-C5_Up
RNA_Wyler_Calu-3_24h_Up
RNA_Wilk_CD4+T-cells_patient-C5_Up
RNA_Blanco-Melo_NHBE_Up
RNA_Wilk_NK-cells_patient-C1A-mild_Up
RNA_Zhang_NK-cells_severe-and-moderate_Up
RNA_Wilk_CD16+Monocytes_patient-C1B-severe_Up
RNA_Wilk_CD14+Monocytes_patient-C6_Up
RNA_Wilk_NK-cells_patient-C4_Up
RNA_Wilk_Dendritic-cells_patient-C1A-mild_Up
RNA_Wilk_CD8+T-cells_patient-C5_Up
RNA_Wilk_NK-cells_patient-C5_Up
RNA_Lamers_intestinal-organoid_expansion_Up
RNA_Wilk_B-cells_patient-C1A-mild_Up
RNA_Wilk_CD16+Monocytes_patient-C1A-mild_Up
RNA_Wilk_Dendritic-cells_patient-C4_Up
RNA_Blanco-Melo_A549-low-MOI_Up
RNA_Sun_Calu-3_12h_Up
RNA_Lieberman_Nasopharynx_Infected_vs_Neg_Up
RNA_Blanco-Melo_Calu-3_Up
Proteome_Klann_Caco-2_24h_Up
RNA_Sun_Calu-3_24h_Up
RNA_Zhang_NK-cells_severe-only_Up
RNA_Wilk_CD14+Monocytes_patient-C4_Up
RNA_Zhang_T-cells_severe-and-moderate_Up
RNA_Wilk_CD16+Monocytes_patient-C7_Up
RNA_Keller_B-cells-CD21pos_infection_Up
RNA_Lieberman_Nasopharynx_High_vs_Low_Up
RNA_Wilk_CD4+T-cells_patient-C1A-mild_Up
RNA_Wilk_CD16+Monocytes_patient-C4_Up
RNA_Wilk_CD8+T-cells_patient-C1A-mild_Up
RNA_Sun_Calu-3_7h_Up
RNA_Wilk_NK-cells_patient-C6_Up
RNA_Wilk_B-cells_patient-C5_Up
RNA_Zhang_B-cells_severe-and-moderate_Up
RNA_Wyler_Calu-3_12h_Up
RNA_Wilk_CD4+T-cells_patient-C6_Up
RNA_Wilk_B-cells_patient-C1B-severe_Down
RNA_Wilk_B-cells_patient-C4_Up
RNA_Wilk_CD8+T-cells_patient-C4_Up
RNA_Wilk_CD14+Monocytes_patient-C7_Up
RNA_Keller_B-cells-Infected_CD21pos_Up
RNA_Wilk_Dendritic-cells_patient-C6_Up
RNA_Zhang_B-cells_moderate-only_Up
RNA_Wilk_CD4+T-cells_patient-C4_Up
RNA_Wilk_Dendritic-cells_patient-C1B-severe_Up
RNA_Wilk_B-cells_patient-C5_Down
RNA_Sun_Calu-3_0h_Up
RNA_Wilk_CD14+Monocytes_patient-C5_Down
RNA_Wilk_B-cells_patient-C4_Down
RNA_Wilk_B-cells_patient-C1B-severe_Up
RNA_Wilk_Dendritic-cells_patient-C7_Up
RNA_Wilk_NK-cells_patient-C3_Up
RNA_Wilk_CD16+Monocytes_patient-C3_Up
RNA_Zhang_Monocytes_moderate-only_Up
RNA_Wilk_CD16+Monocytes_patient-C6_Up
RNA_Wilk_NK-cells_patient-C7_Down
RNA_Zhang_Monocytes_severe-only_Up
RNA_Lamers_intestinal-organoid_differentiated_Up
RNA_Wilk_B-cells_patient-C1A-mild_Down
RNA_Wilk_B-cells_patient-C6_Down
RNA_Keller_B-cells-Infected_CD21pos_Down
RNA_Wilk_B-cells_patient-C6_Up
RNA_Zhang_T-cells_moderate-only_Up
RNA_Wilk_CD14+Monocytes_patient-C3_Up
RNA_Blanco-Melo_A549_Up
RNA_Wilk_CD14+Monocytes_patient-C2_Up
RNA_Wilk_CD4+T-cells_patient-C1B-severe_Up
RNA_Wilk_CD8+T-cells_patient-C1B-severe_Up
RNA_Lieberman_Nasopharynx_High_vs_Low_Down
RNA_Wilk_B-cells_patient-C3_Down
Proteome_Shen_Serum-severe-vs-healthy_Up
RNA_Wilk_B-cells_patient-C7_Up
RNA_Wilk_CD4+T-cells_patient-C7_Up
RNA_Wilk_CD8+T-cells_patient-C3_Up
RNA_Wilk_CD14+Monocytes_patient-C2_Down
RNA_Wilk_NK-cells_patient-C7_Up
RNA_Xiong_PBMC_Up
RNA_Wilk_CD8+T-cells_patient-C2_Down
RNA_Wilk_B-cells_patient-C3_Up
RNA_Wilk_B-cells_patient-C7_Down
RNA_Riva_Vero-E6_24h_Down
RNA_Wilk_B-cells_patient-C2_Down
RNA_Wilk_CD14+Monocytes_patient-C1A-mild_Down
RNA_Appelberg_Huh-7_48h_Down
RNA_Zhang_B-cells_severe-only_Up
Interactome_Laurent_HEK293_24h_NSP2
Interactome_Laurent_HEK293_24h_N
RNA_Wilk_CD8+T-cells_patient-C1A-mild_Down
RNA_Wilk_CD4+T-cells_patient-C3_Up
RNA_Wilk_CD8+T-cells_patient-C5_Down
RNA_Wilk_NK-cells_patient-C1A-mild_Down
RNA_Wilk_CD16+Monocytes_patient-C7_Down
Proteome_Bojkova_Caco-2_6h_Up
Proteome_Shen_Serum-severe-vs-nonsevere_Up
RNA_Wilk_CD14+Monocytes_patient-C4_Down
RNA_Wilk_NK-cells_patient-C1B-severe_Up
RNA_Wilk_CD14+Monocytes_patient-C3_Down
RNA_Stukalov_A549-ACE2_24h_Up
RNA_Wilk_CD8+T-cells_patient-C7_Down
RNA_Blanco-Melo_Lung_Down
RNA_Liao_BALF-mild_Up
RNA_Xiong_BALF_Down
RNA_Xiong_PBMC_Down
RNA_Riva_Vero-E6_24h_Up
RNA_Vanderheiden_pHAE_48h_Up
RNA_Liao_BALF-severe_Up
up DLE
down DLE
down shHMOX1
up shHMOX1
