# Supplementary material for: Evaluation of the Involvement of Heme Oxygenase-1 Expression in Discoid Lupus Erythematosus Lesions
Source: Antioxidants (Basel). 2023 Jun 27;12(7):1352. doi: 10.3390/antiox12071352 (PMC10376595; doi:10.3390/antiox12071352)
Supplement: Supplementary file 1 [file antioxidants-12-01352-s001.zip › File S2.zip!/Enrichment_heatmap/HeatmapSelectedGO.pdf]

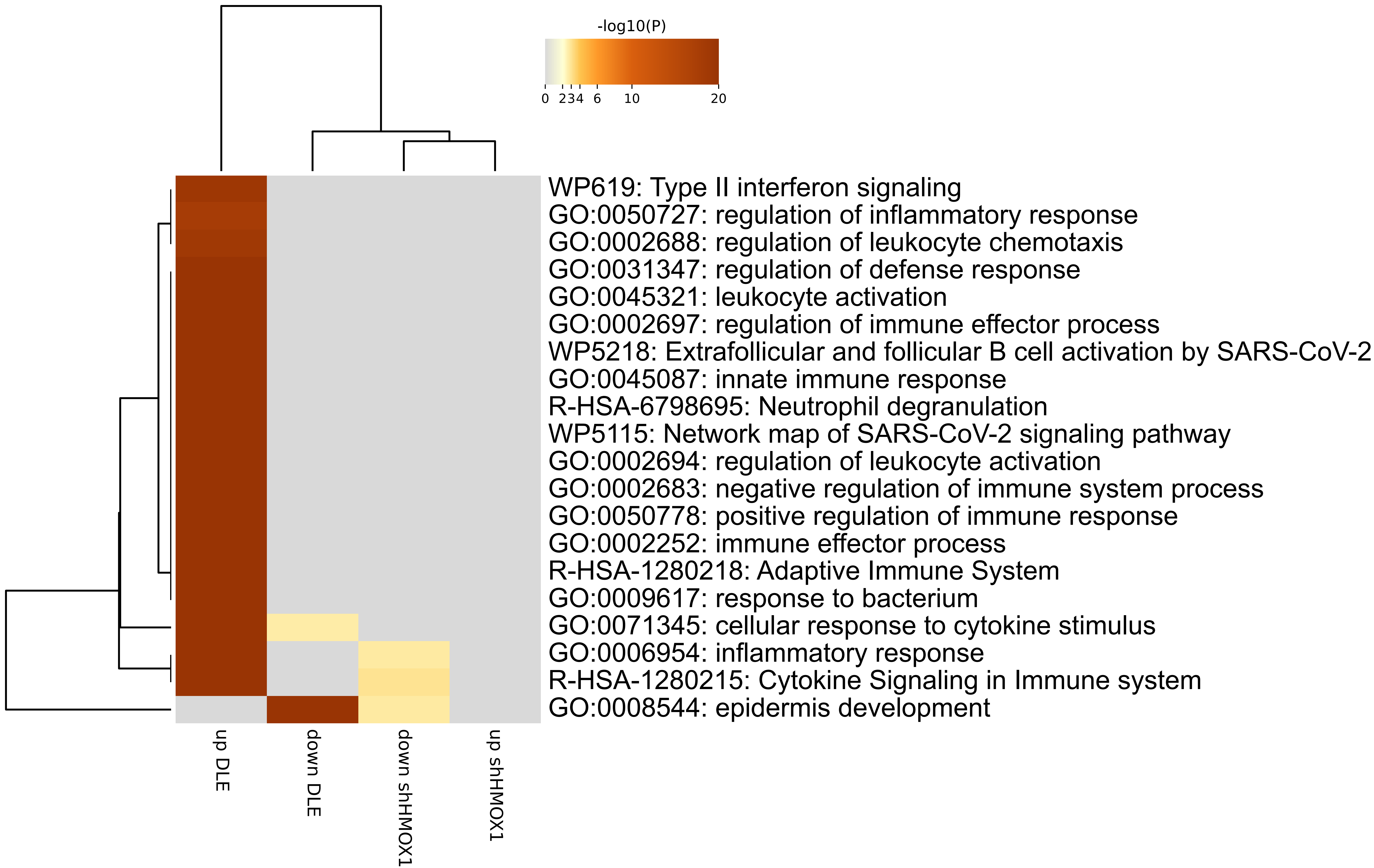

-log10(P)
0
2
3
4
6
10
20
WP619: Type II interferon signaling
GO:0050727: regulation of inflammatory response
GO:0002688: regulation of leukocyte chemotaxis
GO:0031347: regulation of defense response
GO:0045321: leukocyte activation
GO:0002697: regulation of immune effector process
WP5218: Extrafollicular and follicular B cell activation by SARS-CoV-2
GO:0045087: innate immune response
R-HSA-6798695: Neutrophil degranulation
WP5115: Network map of SARS-CoV-2 signaling pathway
GO:0002694: regulation of leukocyte activation
GO:0002683: negative regulation of immune system process
GO:0050778: positive regulation of immune response
GO:0002252: immune effector process
R-HSA-1280218: Adaptive Immune System
GO:0009617: response to bacterium
GO:0071345: cellular response to cytokine stimulus
GO:0006954: inflammatory response
R-HSA-1280215: Cytokine Signaling in Immune system
GO:0008544: epidermis development
up DLE
down DLE
down shHMOX1
up shHMOX1
