# Supplementary material for: Evaluation of the Involvement of Heme Oxygenase-1 Expression in Discoid Lupus Erythematosus Lesions
Source: Antioxidants (Basel). 2023 Jun 27;12(7):1352. doi: 10.3390/antiox12071352 (PMC10376595; doi:10.3390/antiox12071352)
Supplement: Supplementary file 1 [file antioxidants-12-01352-s001.zip › File S2.zip!/Enrichment_heatmap/HeatmapSelectedGOTop100.pdf]

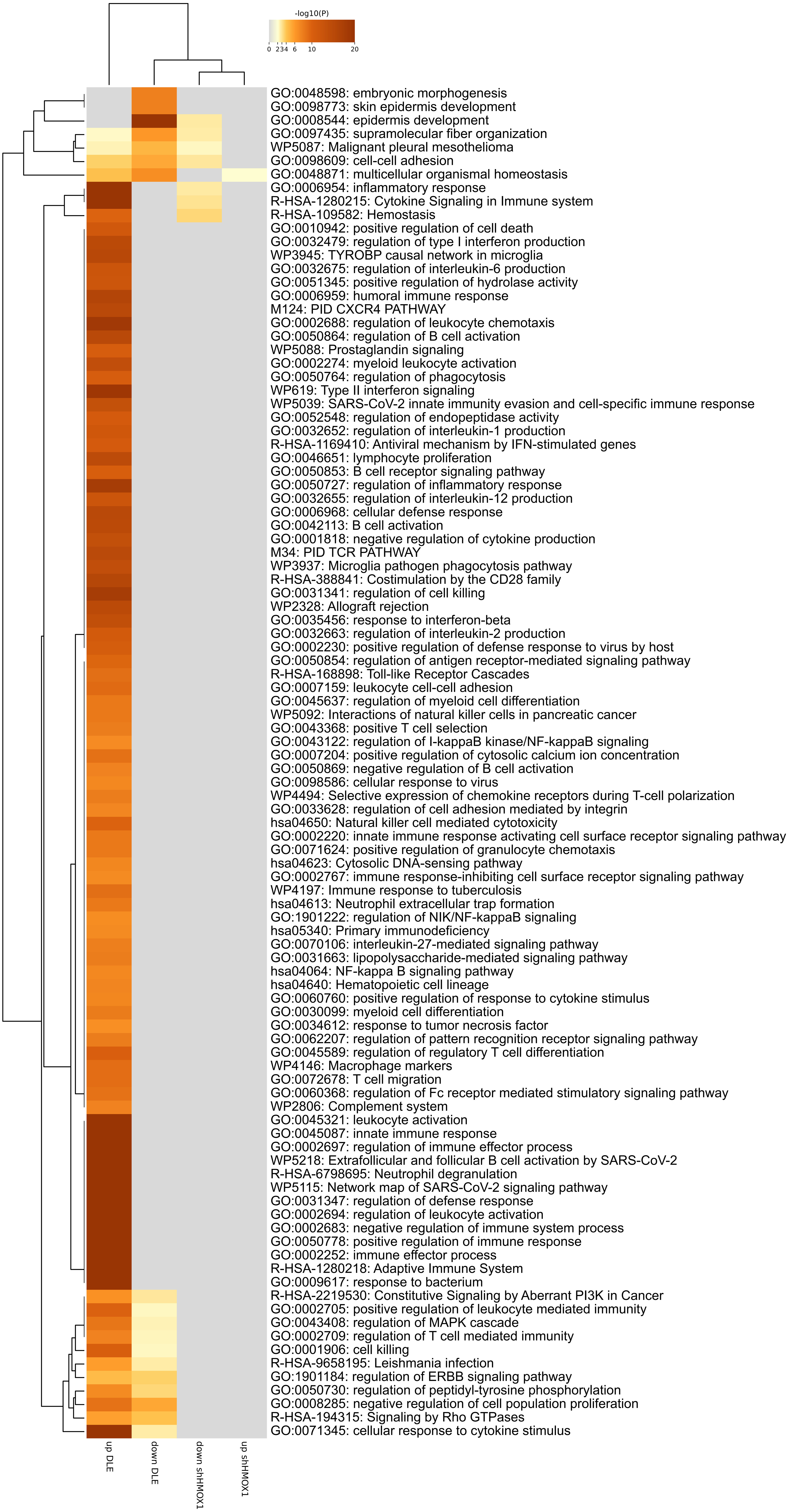

-log10(P)
0 2 3 4 6 10 20
GO:0048598: embryonic morphogenesis
GO:0098773: skin epidermis development
GO:0008544: epidermis development
GO:0097435: supramolecular fiber organization
WP5087: Malignant pleural mesothelioma
GO:0098609: cell-cell adhesion
GO:0048871: multicellular organismal homeostasis
GO:0006954: inflammatory response
R-HSA-1280215: Cytokine Signaling in Immune system
R-HSA-109582: Hemostasis
GO:0010942: positive regulation of cell death
GO:0032479: regulation of type I interferon production
WP3945: TYROBP causal network in microglia
GO:0032675: regulation of interleukin-6 production
GO:0051345: positive regulation of hydrolase activity
GO:0006959: humoral immune response
M124: PID CXCR4 PATHWAY
GO:0002688: regulation of leukocyte chemotaxis
GO:0050864: regulation of B cell activation
WP5088: Prostaglandin signaling
GO:0002274: myeloid leukocyte activation
GO:0050764: regulation of phagocytosis
WP619: Type II interferon signaling
WP5039: SARS-CoV-2 innate immunity evasion and cell-specific immune response
GO:0052548: regulation of endopeptidase activity
GO:0032652: regulation of interleukin-1 production
R-HSA-1169410: Antiviral mechanism by IFN-stimulated genes
GO:0046651: lymphocyte proliferation
GO:0050853: B cell receptor signaling pathway
GO:0050727: regulation of inflammatory response
GO:0032655: regulation of interleukin-12 production
GO:0006968: cellular defense response
GO:0042113: B cell activation
GO:0001818: negative regulation of cytokine production
M34: PID TCR PATHWAY
WP3937: Microglia pathogen phagocytosis pathway
R-HSA-388841: Costimulation by the CD28 family
GO:0031341: regulation of cell killing
WP2328: Allograft rejection
GO:0035456: response to interferon-beta
GO:0032663: regulation of interleukin-2 production
GO:0002230: positive regulation of defense response to virus by host
GO:0050854: regulation of antigen receptor-mediated signaling pathway
R-HSA-168898: Toll-like Receptor Cascades
GO:0007159: leukocyte cell-cell adhesion
GO:0045637: regulation of myeloid cell differentiation
WP5092: Interactions of natural killer cells in pancreatic cancer
GO:0043368: positive T cell selection
GO:0043122: regulation of I-kappaB kinase/NF-kappaB signaling
GO:0007204: positive regulation of cytosolic calcium ion concentration
GO:0050869: negative regulation of B cell activation
GO:0098586: cellular response to virus
WP4494: Selective expression of chemokine receptors during T-cell polarization
GO:0033628: regulation of cell adhesion mediated by integrin
hsa04650: Natural killer cell mediated cytotoxicity
GO:0002220: innate immune response activating cell surface receptor signaling pathway
GO:0071624: positive regulation of granulocyte chemotaxis
hsa04623: Cytosolic DNA-sensing pathway
GO:0002767: immune response-inhibiting cell surface receptor signaling pathway
WP4197: Immune response to tuberculosis
hsa04613: Neutrophil extracellular trap formation
GO:1901222: regulation of NIK/NF-kappaB signaling
hsa05340: Primary immunodeficiency
GO:0070106: interleukin-27-mediated signaling pathway
GO:0031663: lipopolysaccharide-mediated signaling pathway
hsa04064: NF-kappa B signaling pathway
hsa04640: Hematopoietic cell lineage
GO:0060760: positive regulation of response to cytokine stimulus
GO:0030099: myeloid cell differentiation
GO:0034612: response to tumor necrosis factor
GO:0062207: regulation of pattern recognition receptor signaling pathway
GO:0045589: regulation of regulatory T cell differentiation
WP4146: Macrophage markers
GO:0072678: T cell migration
GO:0060368: regulation of Fc receptor mediated stimulatory signaling pathway
WP2806: Complement system
GO:0045321: leukocyte activation
GO:0045087: innate immune response
GO:0002697: regulation of immune effector process
WP5218: Extrafollicular and follicular B cell activation by SARS-CoV-2
R-HSA-6798695: Neutrophil degranulation
WP5115: Network map of SARS-CoV-2 signaling pathway
GO:0031347: regulation of defense response
GO:0002694: regulation of leukocyte activation
GO:0002683: negative regulation of immune system process
GO:0050778: positive regulation of immune response
GO:0002252: immune effector process
R-HSA-1280218: Adaptive Immune System
GO:0009617: response to bacterium
R-HSA-2219530: Constitutive Signaling by Aberrant PI3K in Cancer
GO:0002705: positive regulation of leukocyte mediated immunity
GO:0043408: regulation of MAPK cascade
GO:0002709: regulation of T cell mediated immunity
GO:0001906: cell killing
R-HSA-9658195: Leishmania infection
GO:1901184: regulation of ERBB signaling pathway
GO:0050730: regulation of peptidyl-tyrosine phosphorylation
GO:0008285: negative regulation of cell population proliferation
R-HSA-194315: Signaling by Rho GTPases
GO:0071345: cellular response to cytokine stimulus
up DLE
down DLE
down shHMOX1
up shHMOX1
