# Supplementary material for: Evaluation of the Involvement of Heme Oxygenase-1 Expression in Discoid Lupus Erythematosus Lesions
Source: Antioxidants (Basel). 2023 Jun 27;12(7):1352. doi: 10.3390/antiox12071352 (PMC10376595; doi:10.3390/antiox12071352)
Supplement: Supplementary file 1 [file antioxidants-12-01352-s001.zip › File S2.zip!/Enrichment_heatmap/HeatmapSelectedGOParent.pdf]

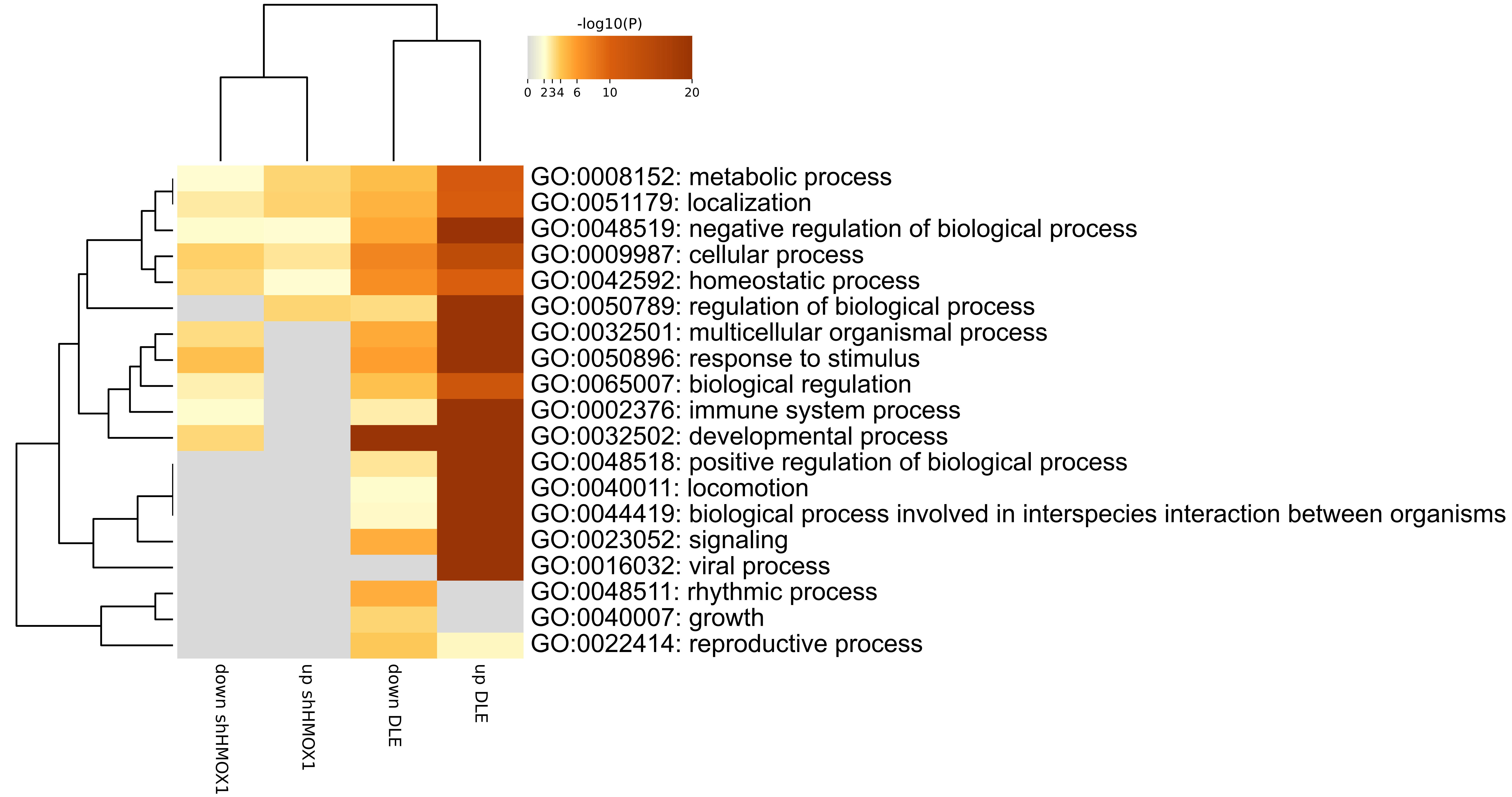
-log10(P)
0
2
3
4
6
10
20
GO:0008152: metabolic process
GO:0051179: localization
GO:0048519: negative regulation of biological process
GO:0009987: cellular process
GO:0042592: homeostatic process
GO:0050789: regulation of biological process
GO:0032501: multicellular organismal process
GO:0050896: response to stimulus
GO:0065007: biological regulation
GO:0002376: immune system process
GO:0032502: developmental process
GO:0048518: positive regulation of biological process
GO:0040011: locomotion
GO:0044419: biological process involved in interspecies interaction between organisms
GO:0023052: signaling
GO:0016032: viral process
GO:0048511: rhythmic process
GO:0040007: growth
GO:0022414: reproductive process
down shHMOX1
up shHMOX1
down DLE
up DLE
