# Supplementary material for: Evaluation of the Involvement of Heme Oxygenase-1 Expression in Discoid Lupus Erythematosus Lesions
Source: Antioxidants (Basel). 2023 Jun 27;12(7):1352. doi: 10.3390/antiox12071352 (PMC10376595; doi:10.3390/antiox12071352)
Supplement: Supplementary file 1 [file antioxidants-12-01352-s001.zip › File S2.zip!/Enrichment_PPI/_FINAL_MCODE_ALL_PPIColorByCluster.pdf]

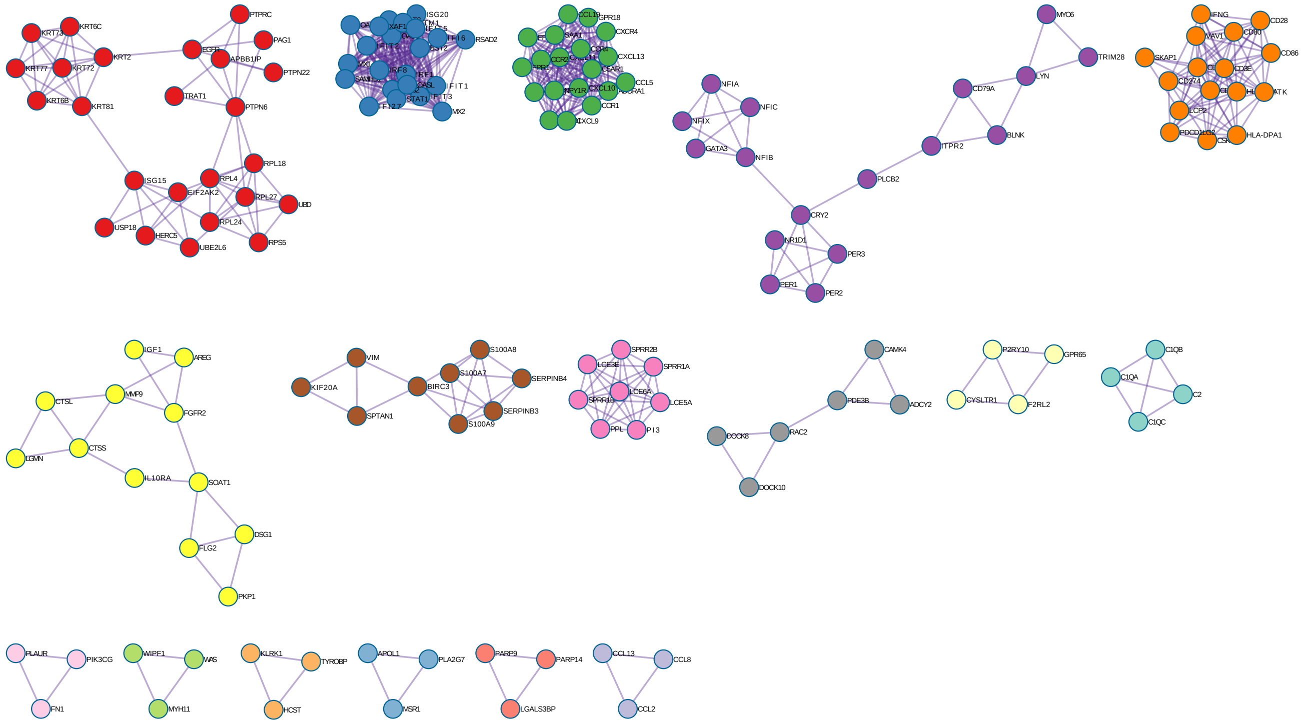

KRT73
KRT6C
KRT2
KRT77
KRT72
KRT6B
KRT81
PTPRC
EGFR
PAG1
APBB1IP
PTPN22
TRAT1
PTPN6
ISG15
RPL4
RPL18
EIF2AK2
RPL27
UBD
USP18
HERC5
UBE2L6
RPL24
RPS5
ISG20
RSAD2
IFIT1
IFIT3
STAT1
MX2
CCR1
CXCL9
CCL5
CXCL13
CXCR4
GPR18
NFIA
NFIX
NFIC
GATA3
NFIB
CRY2
NR1D1
PER3
PER1
PER2
PLCB2
ITPR2
CD79A
LYN
BLNK
MYO6
TRIM28
IFNG
CD28
SKAP1
VAV1
CD86
CD274
LCP2
HLA-DPA1
IGF1
AREG
CTSL
MMP9
FGFR2
CTSS
LGMN
IL10RA
SOAT1
DSG1
FLG2
PKP1
VIM
KIF20A
SPTAN1
BIRC3
S100A8
S100A7
SERPINB4
SERPINB3
S100A9
SPRR2B
SPRR1A
LCE3E
LCE5A
PPL
PI3
CAMK4
PDE3B
ADCY2
RAC2
DOCK8
DOCK10
P2RY10
GPR65
CYSLTR1
F2RL2
C1QB
C1QA
C2
C1QC
PLAUR
PIK3CG
FN1
WIPF1
WAS
MYH11
KLRK1
TYROBP
HCST
APOL1
PLA2G7
MSR1
PARP9
PARP14
LGALS3BP
CCL13
CCL8
CCL2

MCODE1
MCODE2
MCODE3
MCODE4
MCODE5
MCODE6
MCODE7
MCODE8
MCODE9
MCODE10
MCODE11
MCODE12
MCODE13
MCODE14
MCODE15
MCODE16
MCODE17

created by
http://metascape.org
