# Supplementary material for: Evaluation of the Involvement of Heme Oxygenase-1 Expression in Discoid Lupus Erythematosus Lesions
Source: Antioxidants (Basel). 2023 Jun 27;12(7):1352. doi: 10.3390/antiox12071352 (PMC10376595; doi:10.3390/antiox12071352)
Supplement: Supplementary file 1 [file antioxidants-12-01352-s001.zip › File S2.zip!/Enrichment_PPI/up shHMOX1_MCODE_ALL_PPIColorByCluster.pdf]

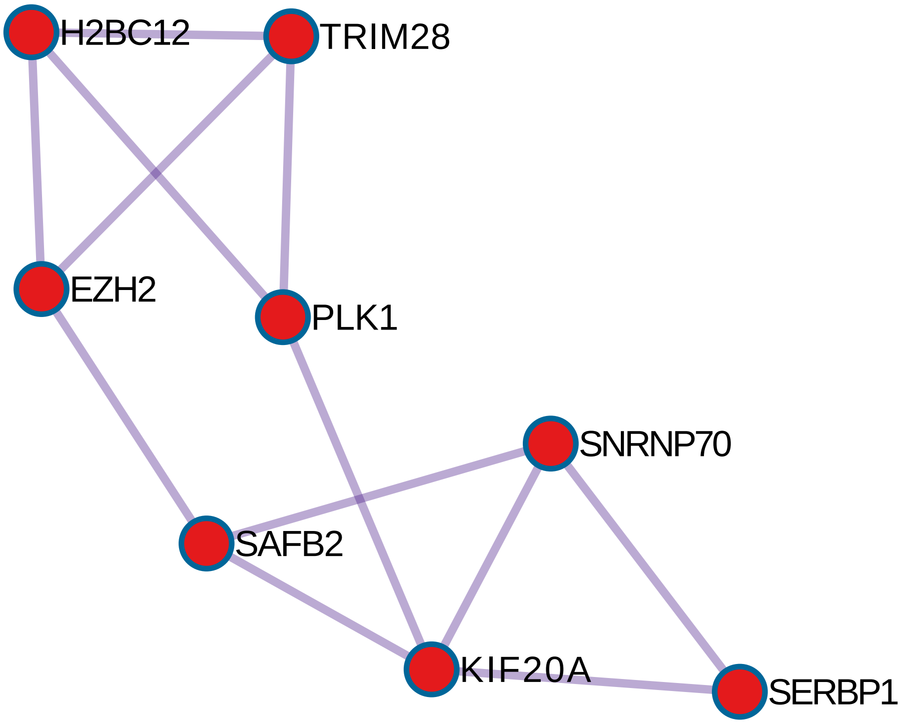
H2BC12
TRIM28
EZH2
PLK1
SNRNP70
SAFB2
KIF20A
SERBP1

MCODE1

created by
http://metascape.org
