# Supplementary material for: Evaluation of the Involvement of Heme Oxygenase-1 Expression in Discoid Lupus Erythematosus Lesions
Source: Antioxidants (Basel). 2023 Jun 27;12(7):1352. doi: 10.3390/antiox12071352 (PMC10376595; doi:10.3390/antiox12071352)
Supplement: Supplementary file 1 [file antioxidants-12-01352-s001.zip › File S2.zip!/Enrichment_PPI/up DLE_MCODE_ALL_PPIColorByCluster.pdf]

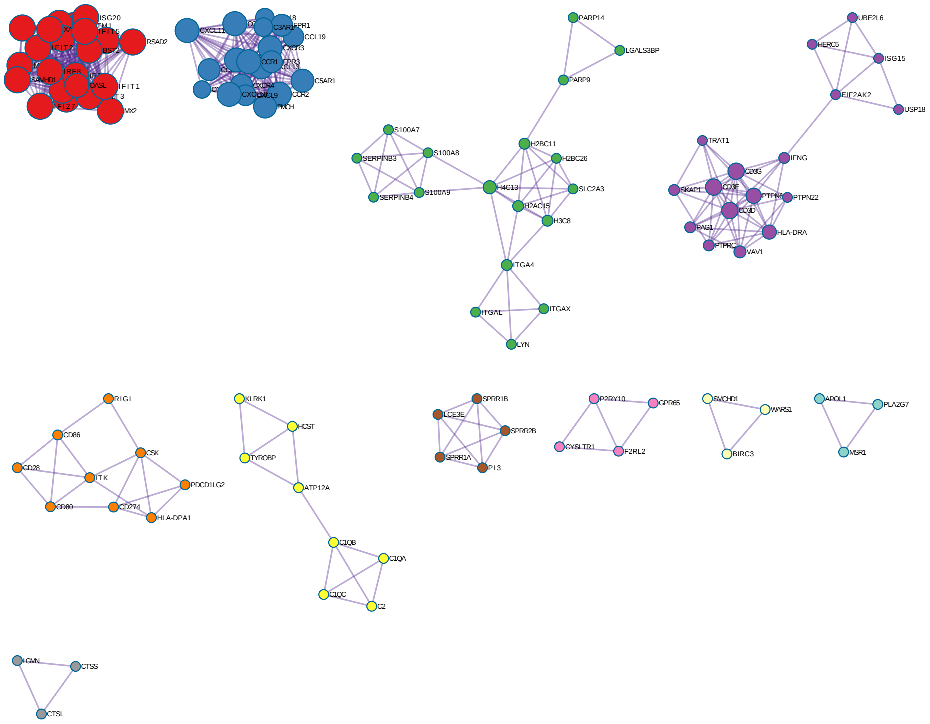

ISG20
RSAD2
OASL
IFIT1
MX2
C3AR1
FPR1
CCL19
CCR1
C5AR1
CCR2
PARP14
LGALS3BP
PARP9
UBE2L6
HERC5
ISG15
EIF2AK2
USP18
S100A7
SERPINB3
S100A8
SERPINB4
S100A9
H2BC11
H2BC26
H4C13
SLC2A3
H2AC15
H3C8
ITGA4
ITGAL
ITGAX
LYN
TRAT1
IFNG
CD3G
SKAP1
CD3E
PTPN22
CD3D
PAG1
HLA-DRA
PTPRC
VAV1
RIGI
CD86
CSK
CD28
ITK
PDCD1LG2
CD80
CD274
HLA-DPA1
KLRK1
HCST
TYROBP
ATP12A
C1QB
C1QA
C1QC
C2
SPRR1B
LCE3E
SPRR2B
SPRR1A
PI3
P2RY10
GPR65
CYSLTR1
F2RL2
SMCHD1
WARS1
BIRC3
APOL1
PLA2G7
MSR1
LGMN
CTSS
CTSL

MCODE1
MCODE2
MCODE3
MCODE4
MCODE5
MCODE6
MCODE7
MCODE8
MCODE9
MCODE10
MCODE11

created by
http://metascape.org
