# Supplementary material for: Evaluation of the Involvement of Heme Oxygenase-1 Expression in Discoid Lupus Erythematosus Lesions
Source: Antioxidants (Basel). 2023 Jun 27;12(7):1352. doi: 10.3390/antiox12071352 (PMC10376595; doi:10.3390/antiox12071352)
Supplement: Supplementary file 1 [file antioxidants-12-01352-s001.zip › File S2.zip!/Enrichment_PPI/down DLE_MCODE_ALL_PPIColorByCluster.pdf]

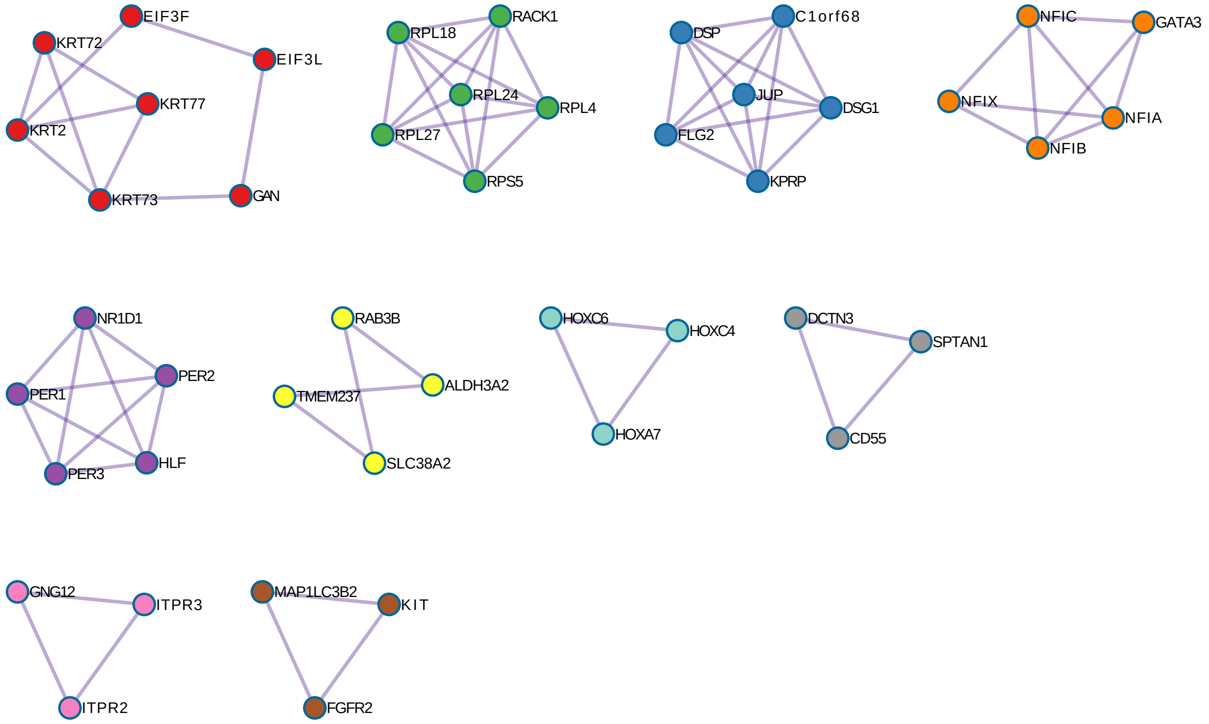
EIF3F
KRT72
EIF3L
KRT77
KRT2
KRT73
GAN
RPL18
RACK1
RPL24
RPL4
RPL27
RPS5
DSP
C1orf68
JUP
DSG1
FLG2
KPRP
NFIC
GATA3
NFIX
NFIA
NFIB
NR1D1
PER2
PER1
HLF
PER3
RAB3B
ALDH3A2
TMEM237
SLC38A2
HOXC6
HOXC4
HOXA7
DCTN3
SPTAN1
CD55
GNG12
ITPR3
ITPR2
MAP1LC3B2
KIT
FGFR2

MCODE1
MCODE2
MCODE3
MCODE4
MCODE5
MCODE6
MCODE7
MCODE8
MCODE9
MCODE10

created by
http://metascape.org
