# Supplementary material for: Evaluation of the Involvement of Heme Oxygenase-1 Expression in Discoid Lupus Erythematosus Lesions
Source: Antioxidants (Basel). 2023 Jun 27;12(7):1352. doi: 10.3390/antiox12071352 (PMC10376595; doi:10.3390/antiox12071352)
Supplement: Supplementary file 1 [file antioxidants-12-01352-s001.zip › File S2.zip!/Enrichment_PPI/down DLE_PPIColorByCluster.pdf]

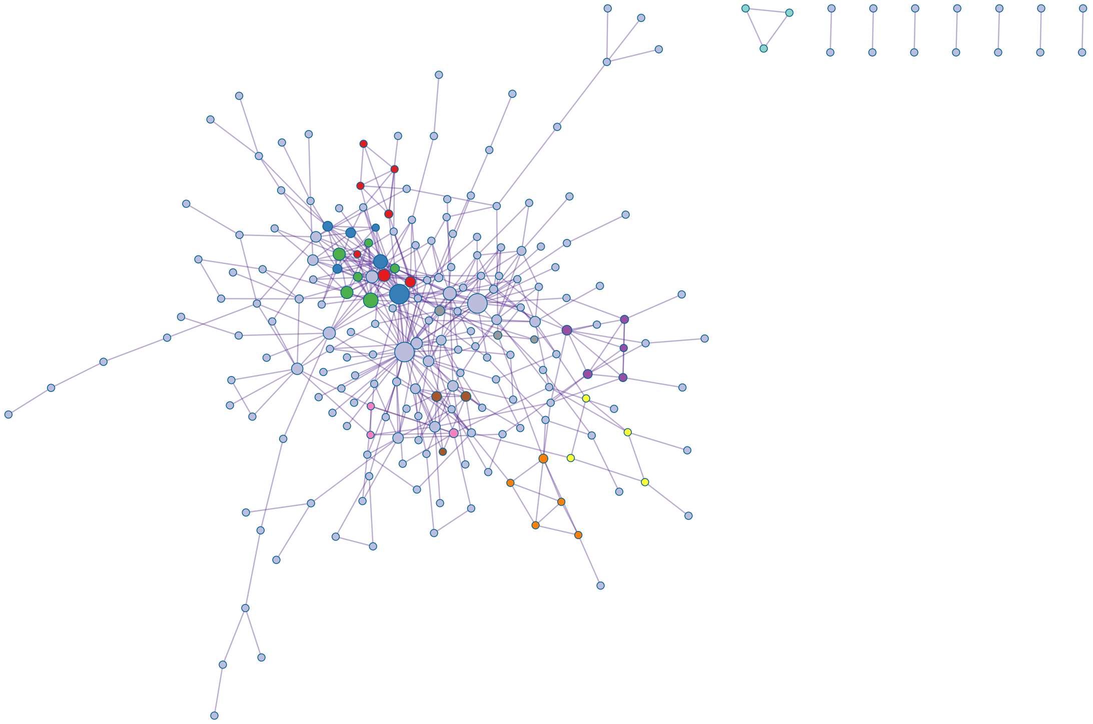

MCODE1
MCODE2
MCODE3
MCODE4
MCODE5
MCODE6
MCODE7
MCODE8
MCODE9
MCODE10

created by
http://metascape.org
