# Supplementary material for: Evaluation of the Involvement of Heme Oxygenase-1 Expression in Discoid Lupus Erythematosus Lesions
Source: Antioxidants (Basel). 2023 Jun 27;12(7):1352. doi: 10.3390/antiox12071352 (PMC10376595; doi:10.3390/antiox12071352)
Supplement: Supplementary file 1 [file antioxidants-12-01352-s001.zip › File S2.zip!/AnalysisReport.html]

## Metascape Gene List Analysis Report

metascape.org^1^

### Heatmap Summary

Figure 1. Heatmap of enriched terms across input gene lists, colored by p-values.

Metascape only visualizes the top 20 clusters. Up to 100 enriched clusters can be viewed here.

The top-level Gene Ontology biological processes can be viewed here.

The heatmap can be interactively viewed using JTreeView^2^ (.cdt, .gtr and .atr files can be found in the Zip package).

### Gene Lists

User-provided gene identifiers are first converted into their corresponding H. sapiens Entrez gene IDs using the latest version of the database (last updated on 2023-01-01). If multiple identifiers correspond to the same Entrez gene ID, they will be considered as a single Entrez gene ID in downstream analyses. Each gene list is assigned a unique color, which is used throughout the analysis. The gene lists are summarized in Table 1.

Table 1. Statistics of input gene lists.

| Name | Total | Unique | Color Code |
| --- | --- | --- | --- |
| down DLE | 331 | 331 |  |
| down shHMOX1 | 48 | 48 |  |
| up DLE | 469 | 469 |  |
| up shHMOX1 | 44 | 44 |  |

The overlaps between these lists are shown in a Circos^3^ plot (Figure 2.a). Another useful representation is to overlap genes based on their functions or shared pathways. The overlaps between gene lists can be significantly improved by considering overlaps between genes sharing the same enriched ontology term(s) (Figure 2.b). Only ontology terms that contain less than 100 genes are used to calculate functional overlaps to avoid linking genes using very general annotation. (We do not want to link all genes, only genes that belong to specific biological processes.)

Figure 2. Overlap between gene lists: (a) only at the gene level, where purple curves link identical genes; (b) including the shared term level, where blue curves link genes that belong to the same enriched ontology term. The inner circle represents gene lists, where hits are arranged along the arc. Genes that hit multiple lists are colored in dark orange, and genes unique to a list are shown in light orange. The publication-quality version of the figures is included in the Zip package as a .svg file under the Overlap\_circos folder (readable by popular web browsers and Adobe Illustrator).

|  |  |
| --- | --- |
|  |  |
|  |  |

### Gene Annotation

The following are the list of annotations retrieved from the latest version of the database (last updated on 2023-01-01) (Table 2).

Table 2. Gene annotations extracted

| Name | Type | Description |
| --- | --- | --- |
| Gene Symbol | Description | Primary HUGO gene symbol. |
| Description | Description | Short description. |
| Biological Process (GO) | Function/Location | Descriptions summarized based on gene ontology database, where up to three most informative GO terms are kept. |
| Kinase Class (UniProt) | Function/Location | Detailed kinase classes. |
| Protein Function (Protein Atlas) | Function/Location | Protein Function (Protein Atlas) |
| Subcellular Location (Protein Atlas) | Function/Location | Subcellular Location (Protein Atlas) |
| Drug (DrugBank) | Genotype/Phenotype/Disease | Drug information for the given gene as target. |
| Canonical Pathways | Ontology | Canonical Pathways |
| Hallmark Gene Sets | Ontology | Hallmark Gene Sets |

### Pathway and Process Enrichment Analysis

For each given gene list, pathway and process enrichment analysis have been carried out with the following ontology sources: KEGG Pathway, GO Biological Processes, Reactome Gene Sets, Canonical Pathways, CORUM, WikiPathways, and PANTHER Pathway. All genes in the genome have been used as the enrichment background. Terms with a p-value < 0.01, a minimum count of 3, and an enrichment factor > 1.5 (the enrichment factor is the ratio between the observed counts and the counts expected by chance) are collected and grouped into clusters based on their membership similarities. More specifically, p-values are calculated based on the cumulative hypergeometric distribution^4^, and q-values are calculated using the Benjamini-Hochberg procedure to account for multiple testings^5^. Kappa scores^6^ are used as the similarity metric when performing hierarchical clustering on the enriched terms, and sub-trees with a similarity of > 0.3 are considered a cluster. The most statistically significant term within a cluster is chosen to represent the cluster.

When multiple gene lists are provided, all lists are merged into one list called "\_FINAL". A term may be found enriched in several individual gene lists and/or in the \_FINAL gene list, and the best p-value among them is chosen as the final p-value. The pathway/process clusters that are found to be of interest (either shared or unique based on specific list enrichment) are used to prioritize the genes that fall into those clusters (membership is presented as 1/0 binary columns in the Excel spreadsheet). Note that individual gene lists containing more than 3000 genes are ignored during the enrichment analysis to avoid superficial terms; this is because long gene lists are often not random and generally trigger too many terms that are not of direct relevance to the biology under study.

Table 3. Top 20 clusters with their representative enriched terms (one per cluster). "Count" is the number of genes in the user-provided lists with membership in the given ontology term. "%" is the percentage of all of the user-provided genes that are found in the given ontology term (only input genes with at least one ontology term annotation are included in the calculation). "Log10(P)" is the p-value in log base 10. "Log10(q)" is the multi-test adjusted p-value in log base 10. \_\_PATTERN\_\_ shows the color code used for the gene lists where the term is found statistically significant, i.e., multiple colors indicate a pathway/process that is shared across multiple lists.

| \_PATTERN\_ | GO | Category | Description | Count | % | Log10(P) | Log10(q) |
| --- | --- | --- | --- | --- | --- | --- | --- |
|  | GO:0045087 | GO Biological Processes | innate immune response | 108 | 23.33 | -72.50 | -68.15 |
|  | GO:0050778 | GO Biological Processes | positive regulation of immune response | 93 | 20.09 | -63.78 | -59.73 |
|  | GO:0045321 | GO Biological Processes | leukocyte activation | 83 | 17.93 | -54.32 | -50.58 |
|  | GO:0031347 | GO Biological Processes | regulation of defense response | 91 | 19.65 | -52.83 | -49.26 |
|  | R-HSA-1280215 | Reactome Gene Sets | Cytokine Signaling in Immune system | 83 | 17.93 | -46.54 | -43.27 |
|  | GO:0002694 | GO Biological Processes | regulation of leukocyte activation | 74 | 15.98 | -43.92 | -40.72 |
|  | GO:0009617 | GO Biological Processes | response to bacterium | 76 | 16.41 | -41.22 | -38.13 |
|  | GO:0002683 | GO Biological Processes | negative regulation of immune system process | 60 | 12.96 | -35.35 | -32.40 |
|  | GO:0006954 | GO Biological Processes | inflammatory response | 61 | 13.17 | -33.72 | -30.78 |
|  | R-HSA-1280218 | Reactome Gene Sets | Adaptive Immune System | 71 | 15.33 | -33.67 | -30.75 |
|  | GO:0071345 | GO Biological Processes | cellular response to cytokine stimulus | 66 | 14.25 | -31.09 | -28.28 |
|  | GO:0002697 | GO Biological Processes | regulation of immune effector process | 48 | 10.37 | -28.53 | -25.79 |
|  | GO:0002252 | GO Biological Processes | immune effector process | 46 | 9.94 | -26.29 | -23.61 |
|  | WP5115 | WikiPathways | Network map of SARS-CoV-2 signaling pathway | 36 | 7.78 | -25.72 | -23.05 |
|  | WP5218 | WikiPathways | Extrafollicular and follicular B cell activation by SARS-CoV-2 | 24 | 5.18 | -24.70 | -22.07 |
|  | GO:0008544 | GO Biological Processes | epidermis development | 34 | 10.59 | -23.02 | -20.46 |
|  | R-HSA-6798695 | Reactome Gene Sets | Neutrophil degranulation | 45 | 9.72 | -21.45 | -18.93 |
|  | WP619 | WikiPathways | Type II interferon signaling | 16 | 3.46 | -19.16 | -16.71 |
|  | GO:0002688 | GO Biological Processes | regulation of leukocyte chemotaxis | 24 | 5.18 | -18.82 | -16.42 |
|  | GO:0050727 | GO Biological Processes | regulation of inflammatory response | 38 | 8.21 | -17.91 | -15.54 |

To further capture the relationships between the terms, a subset of enriched terms has been selected and rendered as a network plot, where terms with a similarity > 0.3 are connected by edges. We select the terms with the best p-values from each of the 20 clusters, with the constraint that there are no more than 15 terms per cluster and no more than 250 terms in total. The network is visualized using Cytoscape^7^, where each node represents an enriched term and is colored first by its cluster ID (Figure 3.a) and then by its p-value (Figure 3.b). These networks can be interactively viewed in Cytoscape through the .cys files (contained in the Zip package, which also contains a publication-quality version as a PDF) or within a browser by clicking on the web icon. For clarity, term labels are only shown for one term per cluster, so it is recommended to use Cytoscape or a browser to visualize the network in order to inspect all node labels. We can also export the network into a PDF file within Cytoscape, and then edit the labels using Adobe Illustrator for publication purposes. To switch off all labels, delete the "Label" mapping under the "Style" tab within Cytoscape, and then export the network view.

Figure 3. Network of enriched terms: (a) colored by cluster ID, where nodes that share the same cluster ID are typically close to each other; (b) colored by p-value, where terms containing more genes tend to have a more significant p-value.

|  |  |
| --- | --- |
|  |  |
|  |  |

In the case of when multiple gene lists are provided, the nodes are represented as pie charts, where the size of a pie is proportional to the total number of hits that fall into that specific term. The pie charts are color-coded based on the gene list identities, where the size of a slice represents the percentage of genes under the term that originated from the corresponding gene list. This plot is particularly useful for visualizing whether the terms are shared by multiple lists or unique to a specific list, as well as for understanding how these terms associate with each other within the biological context of the meta study (Figure 4).

Figure 4. Network of enriched terms represented as pie charts, where pies are color-coded based on the identities of the gene lists.

### Protein-protein Interaction Enrichment Analysis

For each given gene list, protein-protein interaction enrichment analysis has been carried out with the following databases: STRING^8^, BioGrid^9^, OmniPath^10^, InWeb\_IM^11^.Only physical interactions in STRING (physical score > 0.132) and BioGrid are used (details). The resultant network contains the subset of proteins that form physical interactions with at least one other member in the list. If the network contains between 3 and 500 proteins, the Molecular Complex Detection (MCODE) algorithm^12^ has been applied to identify densely connected network components. The MCODE networks identified for individual gene lists have been gathered and are shown in Figure 5.

Pathway and process enrichment analysis has been applied to each MCODE component independently, and the three best-scoring terms by p-value have been retained as the functional description of the corresponding components, shown in the tables underneath corresponding network plots within Figure 5.

Figure 5. Protein-protein interaction network and MCODE components identified in the gene lists.

#### down DLE (Full Connection)

| GO | Description | Log10(P) |
| --- | --- | --- |
| GO:0008544 | epidermis development | -19.9 |
| GO:0043588 | skin development | -17.2 |
| GO:0030855 | epithelial cell differentiation | -12.1 |

#### down DLE (Keep MCODE Nodes Only)

| Color | MCODE | GO | Description | Log10(P) |
| --- | --- | --- | --- | --- |
|  | MCODE\_1 | GO:0045109 | intermediate filament organization | -9.0 |
|  | MCODE\_1 | GO:0031424 | keratinization | -8.8 |
|  | MCODE\_1 | GO:0045104 | intermediate filament cytoskeleton organization | -8.6 |
|  | MCODE\_2 | R-HSA-6798695 | Neutrophil degranulation | -6.0 |
|  | MCODE\_2 | R-HSA-6809371 | Formation of the cornified envelope | -5.8 |
|  | MCODE\_2 | GO:0045216 | cell-cell junction organization | -5.4 |
|  | MCODE\_3 | GO:0002181 | cytoplasmic translation | -14.4 |
|  | MCODE\_3 | CORUM:306 | Ribosome, cytoplasmic | -12.2 |
|  | MCODE\_3 | WP477 | Cytoplasmic ribosomal proteins | -11.9 |
|  | MCODE\_4 | hsa04710 | Circadian rhythm | -11.2 |
|  | MCODE\_4 | GO:0048511 | rhythmic process | -10.2 |
|  | MCODE\_4 | GO:0032922 | circadian regulation of gene expression | -9.9 |
|  | MCODE\_5 | R-HSA-73980 | RNA Polymerase III Transcription Termination | -11.9 |
|  | MCODE\_5 | R-HSA-74158 | RNA Polymerase III Transcription | -10.8 |
|  | MCODE\_5 | R-HSA-749476 | RNA Polymerase III Abortive And Retractive Initiation | -10.8 |
|  | MCODE\_8 | R-HSA-381676 | Glucagon-like Peptide-1 (GLP1) regulates insulin secretion | -8.6 |
|  | MCODE\_8 | R-HSA-4086398 | Ca2+ pathway | -8.1 |
|  | MCODE\_8 | R-HSA-422356 | Regulation of insulin secretion | -7.8 |
|  | MCODE\_9 | R-HSA-6807878 | COPI-mediated anterograde transport | -7.4 |
|  | MCODE\_9 | R-HSA-199977 | ER to Golgi Anterograde Transport | -6.9 |
|  | MCODE\_9 | R-HSA-948021 | Transport to the Golgi and subsequent modification | -6.6 |
|  | MCODE\_10 | GO:0048706 | embryonic skeletal system development | -7.1 |
|  | MCODE\_10 | GO:0009952 | anterior/posterior pattern specification | -6.5 |
|  | MCODE\_10 | GO:0003002 | regionalization | -5.6 |

#### down shHMOX1 (Full Connection)

| GO | Description | Log10(P) |
| --- | --- | --- |
| WP2814 | Mammary gland development pathway - Puberty (Stage 2 of 4) | -7.0 |
| R-HSA-6785807 | Interleukin-4 and Interleukin-13 signaling | -6.0 |
| R-HSA-109582 | Hemostasis | -5.4 |

#### up DLE (Full Connection)

| GO | Description | Log10(P) |
| --- | --- | --- |
| GO:0045087 | innate immune response | -80.4 |
| GO:0050778 | positive regulation of immune response | -66.7 |
| GO:0045321 | leukocyte activation | -57.2 |

#### up DLE (Keep MCODE Nodes Only)

| Color | MCODE | GO | Description | Log10(P) |
| --- | --- | --- | --- | --- |
|  | MCODE\_1 | R-HSA-909733 | Interferon alpha/beta signaling | -59.2 |
|  | MCODE\_1 | R-HSA-913531 | Interferon Signaling | -48.4 |
|  | MCODE\_1 | GO:0051607 | defense response to virus | -39.5 |
|  | MCODE\_2 | R-HSA-375276 | Peptide ligand-binding receptors | -38.2 |
|  | MCODE\_2 | R-HSA-373076 | Class A/1 (Rhodopsin-like receptors) | -37.3 |
|  | MCODE\_2 | R-HSA-500792 | GPCR ligand binding | -34.5 |
|  | MCODE\_3 | hsa04613 | Neutrophil extracellular trap formation | -9.0 |
|  | MCODE\_3 | R-HSA-3214815 | HDACs deacetylate histones | -8.7 |
|  | MCODE\_3 | R-HSA-6799990 | Metal sequestration by antimicrobial proteins | -8.4 |
|  | MCODE\_4 | R-HSA-202427 | Phosphorylation of CD3 and TCR zeta chains | -18.1 |
|  | MCODE\_4 | M34 | PID TCR PATHWAY | -17.2 |
|  | MCODE\_4 | M124 | PID CXCR4 PATHWAY | -15.6 |
|  | MCODE\_5 | R-HSA-388841 | Costimulation by the CD28 family | -17.1 |
|  | MCODE\_5 | GO:0031295 | T cell costimulation | -15.4 |
|  | MCODE\_5 | GO:0031294 | lymphocyte costimulation | -15.3 |
|  | MCODE\_6 | CORUM:6418 | C1q complex | -10.9 |
|  | MCODE\_6 | R-HSA-166663 | Initial triggering of complement | -10.7 |
|  | MCODE\_6 | WP545 | Complement activation | -10.7 |
|  | MCODE\_7 | R-HSA-6809371 | Formation of the cornified envelope | -11.9 |
|  | MCODE\_7 | R-HSA-6805567 | Keratinization | -10.8 |
|  | MCODE\_7 | GO:0031424 | keratinization | -9.6 |
|  | MCODE\_8 | GO:0007204 | positive regulation of cytosolic calcium ion concentration | -9.0 |
|  | MCODE\_8 | R-HSA-416476 | G alpha (q) signalling events | -8.6 |
|  | MCODE\_8 | GO:0051482 | positive regulation of cytosolic calcium ion concentration involved in phospholipase C-activating G protein-coupled signaling pathway | -8.4 |
|  | MCODE\_9 | R-HSA-1679131 | Trafficking and processing of endosomal TLR | -10.2 |
|  | MCODE\_9 | GO:0019886 | antigen processing and presentation of exogenous peptide antigen via MHC class II | -9.0 |
|  | MCODE\_9 | GO:0002495 | antigen processing and presentation of peptide antigen via MHC class II | -8.9 |

#### up shHMOX1 (Full Connection)

| GO | Description | Log10(P) |
| --- | --- | --- |
| M256 | PID TAP63 PATHWAY | -4.8 |
| R-HSA-68886 | M Phase | -4.5 |
| R-HSA-2262752 | Cellular responses to stress | -4.2 |

#### up shHMOX1 (Keep MCODE Nodes Only)

| Color | MCODE | GO | Description | Log10(P) |
| --- | --- | --- | --- | --- |
|  | MCODE\_1 | R-HSA-9609690 | HCMV Early Events | -5.3 |
|  | MCODE\_1 | R-HSA-9609646 | HCMV Infection | -5.1 |
|  | MCODE\_1 | GO:1903311 | regulation of mRNA metabolic process | -4.2 |

#### All lists merged Colored by Counts(Full Connection)

| GO | Description | Log10(P) |
| --- | --- | --- |
| GO:0045087 | innate immune response | -75.5 |
| GO:0050778 | positive regulation of immune response | -62.8 |
| GO:0001775 | cell activation | -59.6 |

#### All lists merged Colored by Counts(Keep MCODE Nodes Only)

| Color | MCODE | GO | Description | Log10(P) |
| --- | --- | --- | --- | --- |
|  | MCODE\_1 | GO:0045109 | intermediate filament organization | -12.8 |
|  | MCODE\_1 | GO:0031424 | keratinization | -12.4 |
|  | MCODE\_1 | GO:0045104 | intermediate filament cytoskeleton organization | -12.1 |
|  | MCODE\_2 | R-HSA-909733 | Interferon alpha/beta signaling | -59.2 |
|  | MCODE\_2 | R-HSA-913531 | Interferon Signaling | -48.4 |
|  | MCODE\_2 | GO:0051607 | defense response to virus | -39.5 |
|  | MCODE\_3 | R-HSA-418594 | G alpha (i) signalling events | -37.8 |
|  | MCODE\_3 | R-HSA-373076 | Class A/1 (Rhodopsin-like receptors) | -37.3 |
|  | MCODE\_3 | R-HSA-375276 | Peptide ligand-binding receptors | -35.1 |
|  | MCODE\_4 | hsa04710 | Circadian rhythm | -11.1 |
|  | MCODE\_4 | M95 | PID CIRCADIAN PATHWAY | -9.9 |
|  | MCODE\_4 | GO:0032922 | circadian regulation of gene expression | -9.4 |
|  | MCODE\_5 | R-HSA-388841 | Costimulation by the CD28 family | -28.9 |
|  | MCODE\_5 | M34 | PID TCR PATHWAY | -26.2 |
|  | MCODE\_5 | GO:0050778 | positive regulation of immune response | -21.7 |
|  | MCODE\_6 | R-HSA-1679131 | Trafficking and processing of endosomal TLR | -7.9 |
|  | MCODE\_6 | M5885 | NABA MATRISOME ASSOCIATED | -6.7 |
|  | MCODE\_6 | GO:0019886 | antigen processing and presentation of exogenous peptide antigen via MHC class II | -6.7 |
|  | MCODE\_7 | R-HSA-6799990 | Metal sequestration by antimicrobial proteins | -9.4 |
|  | MCODE\_7 | GO:0035425 | autocrine signaling | -9.2 |
|  | MCODE\_7 | GO:0052548 | regulation of endopeptidase activity | -7.7 |
|  | MCODE\_8 | R-HSA-6809371 | Formation of the cornified envelope | -19.0 |
|  | MCODE\_8 | R-HSA-6805567 | Keratinization | -17.2 |
|  | MCODE\_8 | GO:0031424 | keratinization | -17.2 |
|  | MCODE\_9 | hsa04024 | cAMP signaling pathway | -7.4 |
|  | MCODE\_9 | hsa04371 | Apelin signaling pathway | -5.7 |
|  | MCODE\_9 | R-HSA-9006925 | Intracellular signaling by second messengers | -4.7 |
|  | MCODE\_10 | R-HSA-166663 | Initial triggering of complement | -12.6 |
|  | MCODE\_10 | WP545 | Complement activation | -12.6 |
|  | MCODE\_10 | GO:0006958 | complement activation, classical pathway | -12.1 |
|  | MCODE\_11 | GO:0007204 | positive regulation of cytosolic calcium ion concentration | -9.0 |
|  | MCODE\_11 | R-HSA-416476 | G alpha (q) signalling events | -8.6 |
|  | MCODE\_11 | GO:0051482 | positive regulation of cytosolic calcium ion concentration involved in phospholipase C-activating G protein-coupled signaling pathway | -8.4 |
|  | MCODE\_12 | GO:0048245 | eosinophil chemotaxis | -9.2 |
|  | MCODE\_12 | GO:0072677 | eosinophil migration | -9.1 |
|  | MCODE\_12 | GO:0002548 | monocyte chemotaxis | -8.5 |
|  | MCODE\_15 | hsa04650 | Natural killer cell mediated cytotoxicity | -7.1 |
|  | MCODE\_15 | R-HSA-198933 | Immunoregulatory interactions between a Lymphoid and a non-Lymphoid cell | -7.1 |
|  | MCODE\_15 | R-HSA-1280218 | Adaptive Immune System | -4.8 |
|  | MCODE\_16 | R-HSA-195258 | RHO GTPase Effectors | -5.9 |
|  | MCODE\_16 | GO:0030036 | actin cytoskeleton organization | -5.3 |
|  | MCODE\_16 | GO:0097435 | supramolecular fiber organization | -5.2 |
|  | MCODE\_17 | GO:0009611 | response to wounding | -5.5 |
|  | MCODE\_17 | R-HSA-109582 | Hemostasis | -5.1 |

#### All lists merged Colored by Cluster(Full Connection)

| GO | Description | Log10(P) |
| --- | --- | --- |
| GO:0045087 | innate immune response | -75.5 |
| GO:0050778 | positive regulation of immune response | -62.8 |
| GO:0001775 | cell activation | -59.6 |

#### All lists merged Colored by Cluster(Keep MCODE Nodes Only)

| Color | MCODE | GO | Description | Log10(P) |
| --- | --- | --- | --- | --- |
|  | MCODE\_1 | GO:0045109 | intermediate filament organization | -12.8 |
|  | MCODE\_1 | GO:0031424 | keratinization | -12.4 |
|  | MCODE\_1 | GO:0045104 | intermediate filament cytoskeleton organization | -12.1 |
|  | MCODE\_2 | R-HSA-909733 | Interferon alpha/beta signaling | -59.2 |
|  | MCODE\_2 | R-HSA-913531 | Interferon Signaling | -48.4 |
|  | MCODE\_2 | GO:0051607 | defense response to virus | -39.5 |
|  | MCODE\_3 | R-HSA-418594 | G alpha (i) signalling events | -37.8 |
|  | MCODE\_3 | R-HSA-373076 | Class A/1 (Rhodopsin-like receptors) | -37.3 |
|  | MCODE\_3 | R-HSA-375276 | Peptide ligand-binding receptors | -35.1 |
|  | MCODE\_4 | hsa04710 | Circadian rhythm | -11.1 |
|  | MCODE\_4 | M95 | PID CIRCADIAN PATHWAY | -9.9 |
|  | MCODE\_4 | GO:0032922 | circadian regulation of gene expression | -9.4 |
|  | MCODE\_5 | R-HSA-388841 | Costimulation by the CD28 family | -28.9 |
|  | MCODE\_5 | M34 | PID TCR PATHWAY | -26.2 |
|  | MCODE\_5 | GO:0050778 | positive regulation of immune response | -21.7 |
|  | MCODE\_6 | R-HSA-1679131 | Trafficking and processing of endosomal TLR | -7.9 |
|  | MCODE\_6 | M5885 | NABA MATRISOME ASSOCIATED | -6.7 |
|  | MCODE\_6 | GO:0019886 | antigen processing and presentation of exogenous peptide antigen via MHC class II | -6.7 |
|  | MCODE\_7 | R-HSA-6799990 | Metal sequestration by antimicrobial proteins | -9.4 |
|  | MCODE\_7 | GO:0035425 | autocrine signaling | -9.2 |
|  | MCODE\_7 | GO:0052548 | regulation of endopeptidase activity | -7.7 |
|  | MCODE\_8 | R-HSA-6809371 | Formation of the cornified envelope | -19.0 |
|  | MCODE\_8 | R-HSA-6805567 | Keratinization | -17.2 |
|  | MCODE\_8 | GO:0031424 | keratinization | -17.2 |
|  | MCODE\_9 | hsa04024 | cAMP signaling pathway | -7.4 |
|  | MCODE\_9 | hsa04371 | Apelin signaling pathway | -5.7 |
|  | MCODE\_9 | R-HSA-9006925 | Intracellular signaling by second messengers | -4.7 |
|  | MCODE\_10 | R-HSA-166663 | Initial triggering of complement | -12.6 |
|  | MCODE\_10 | WP545 | Complement activation | -12.6 |
|  | MCODE\_10 | GO:0006958 | complement activation, classical pathway | -12.1 |
|  | MCODE\_11 | GO:0007204 | positive regulation of cytosolic calcium ion concentration | -9.0 |
|  | MCODE\_11 | R-HSA-416476 | G alpha (q) signalling events | -8.6 |
|  | MCODE\_11 | GO:0051482 | positive regulation of cytosolic calcium ion concentration involved in phospholipase C-activating G protein-coupled signaling pathway | -8.4 |
|  | MCODE\_12 | GO:0048245 | eosinophil chemotaxis | -9.2 |
|  | MCODE\_12 | GO:0072677 | eosinophil migration | -9.1 |
|  | MCODE\_12 | GO:0002548 | monocyte chemotaxis | -8.5 |
|  | MCODE\_15 | hsa04650 | Natural killer cell mediated cytotoxicity | -7.1 |
|  | MCODE\_15 | R-HSA-198933 | Immunoregulatory interactions between a Lymphoid and a non-Lymphoid cell | -7.1 |
|  | MCODE\_15 | R-HSA-1280218 | Adaptive Immune System | -4.8 |
|  | MCODE\_16 | R-HSA-195258 | RHO GTPase Effectors | -5.9 |
|  | MCODE\_16 | GO:0030036 | actin cytoskeleton organization | -5.3 |
|  | MCODE\_16 | GO:0097435 | supramolecular fiber organization | -5.2 |
|  | MCODE\_17 | GO:0009611 | response to wounding | -5.5 |
|  | MCODE\_17 | R-HSA-109582 | Hemostasis | -5.1 |

### Quality Control and Association Analysis

Gene list enrichments are identified in the following ontology categories: COVID, TRRUST, Transcription\_Factor\_Targets, Cell\_Type\_Signatures, DisGeNET, PaGenBase. All genes in the genome have been used as the enrichment background. Terms with a p-value < 0.01, a minimum count of 3, and an enrichment factor > 1.5 (the enrichment factor is the ratio between the observed counts and the counts expected by chance) are collected and grouped into clusters based on their membership similarities. The top few enriched clusters (one term per cluster) are shown in the Figure 6-11. The algorithm used here is the same as that is used for pathway and process enrichment analysis.

Figure 6. Summary of enrichment analysis in COVID^13^.

| \_PATTERN\_ | GO | Description | Count | % | Log10(P) | Log10(q) |
| --- | --- | --- | --- | --- | --- | --- |
|  | COVID011 | RNA\_Blanco-Melo\_A549-ACE2-ruxolitinib\_Down | 89 | 19.00 | -100.00 | -96.00 |
|  | COVID017 | RNA\_Blanco-Melo\_Lung\_Down | 96 | 21.00 | -100.00 | -96.00 |
|  | COVID353 | RNA\_Zhang\_Monocytes\_severe-and-moderate\_Up | 75 | 16.00 | -100.00 | -96.00 |
|  | COVID050 | RNA\_Wyler\_Calu-3\_24h\_Up | 93 | 20.00 | -95.00 | -91.00 |
|  | COVID048 | RNA\_Wyler\_Calu-3\_12h\_Up | 85 | 18.00 | -83.00 | -79.00 |
|  | COVID016 | RNA\_Blanco-Melo\_Calu-3\_Up | 84 | 18.00 | -81.00 | -78.00 |
|  | COVID359 | RNA\_Zhang\_T-cells\_severe-and-moderate\_Up | 63 | 14.00 | -76.00 | -73.00 |
|  | COVID356 | RNA\_Zhang\_NK-cells\_severe-and-moderate\_Up | 53 | 11.00 | -70.00 | -67.00 |
|  | COVID241 | RNA\_Lieberman\_Nasopharynx\_High\_vs\_Low\_Up | 64 | 14.00 | -70.00 | -67.00 |
|  | COVID244 | RNA\_Wilk\_CD14+Monocytes\_patient-C1A-mild\_Up | 46 | 9.90 | -62.00 | -59.00 |
|  | COVID297 | RNA\_Wilk\_NK-cells\_patient-C5\_Up | 39 | 8.40 | -60.00 | -57.00 |
|  | COVID362 | RNA\_Zhang\_B-cells\_severe-and-moderate\_Up | 43 | 9.30 | -58.00 | -55.00 |
|  | COVID239 | RNA\_Lieberman\_Nasopharynx\_Infected\_vs\_Neg\_Up | 35 | 7.60 | -57.00 | -54.00 |
|  | COVID256 | RNA\_Wilk\_CD14+Monocytes\_patient-C6\_Up | 53 | 11.00 | -56.00 | -53.00 |
|  | COVID254 | RNA\_Wilk\_CD14+Monocytes\_patient-C5\_Up | 46 | 9.90 | -55.00 | -52.00 |
|  | COVID266 | RNA\_Wilk\_CD16+Monocytes\_patient-C4\_Up | 41 | 8.90 | -53.00 | -50.00 |
|  | COVID252 | RNA\_Wilk\_CD14+Monocytes\_patient-C4\_Up | 41 | 8.90 | -53.00 | -50.00 |
|  | COVID038 | RNA\_Sun\_Calu-3\_12h\_Up | 63 | 14.00 | -52.00 | -49.00 |
|  | COVID329 | RNA\_Wilk\_CD4+T-cells\_patient-C5\_Up | 31 | 6.70 | -50.00 | -47.00 |
|  | COVID282 | RNA\_Wilk\_Dendritic-cells\_patient-C5\_Up | 38 | 8.20 | -49.00 | -46.00 |

Figure 7. Summary of enrichment analysis in TRRUST.

| \_PATTERN\_ | GO | Description | Count | % | Log10(P) | Log10(q) |
| --- | --- | --- | --- | --- | --- | --- |
|  | TRR00875 | Regulated by: NFKB1 | 40 | 8.60 | -23.00 | -21.00 |
|  | TRR01158 | Regulated by: RELA | 39 | 8.40 | -22.00 | -20.00 |
|  | TRR01275 | Regulated by: STAT1 | 19 | 4.10 | -15.00 | -13.00 |
|  | TRR01264 | Regulated by: SPI1 | 16 | 3.50 | -15.00 | -13.00 |
|  | TRR00602 | Regulated by: IRF1 | 13 | 2.80 | -12.00 | -9.90 |
|  | TRR01256 | Regulated by: SP1 | 28 | 6.00 | -7.80 | -6.10 |
|  | TRR01424 | Regulated by: TRERF1 | 5 | 1.10 | -7.80 | -6.00 |
|  | TRR00645 | Regulated by: JUN | 15 | 3.20 | -7.20 | -5.40 |
|  | TRR00016 | Regulated by: ARNTL | 4 | 1.20 | -6.70 | -5.00 |
|  | TRR00129 | Regulated by: CREB5 | 5 | 1.10 | -6.00 | -4.40 |
|  | TRR00109 | Regulated by: CEBPA | 8 | 1.70 | -5.50 | -3.90 |
|  | TRR00270 | Regulated by: EP300 | 8 | 1.70 | -5.30 | -3.80 |
|  | TRR00892 | Regulated by: NPAS2 | 3 | 0.93 | -5.30 | -3.80 |
|  | TRR01277 | Regulated by: STAT3 | 12 | 2.60 | -5.10 | -3.60 |
|  | TRR01276 | Regulated by: STAT2 | 4 | 0.86 | -4.60 | -3.10 |
|  | TRR00011 | Regulated by: AR | 4 | 8.30 | -4.60 | -3.10 |
|  | TRR00119 | Regulated by: CLOCK | 4 | 1.20 | -4.60 | -3.10 |
|  | TRR01157 | Regulated by: REL | 5 | 1.10 | -4.40 | -2.90 |
|  | TRR00604 | Regulated by: IRF3 | 4 | 0.86 | -4.20 | -2.80 |
|  | TRR01419 | Regulated by: TP53 | 4 | 9.10 | -3.90 | -2.50 |

Figure 8. Summary of enrichment analysis in Transcription Factor Targets^14^.

| \_PATTERN\_ | GO | Description | Count | % | Log10(P) | Log10(q) |
| --- | --- | --- | --- | --- | --- | --- |
|  | M16200 | ISRE 01 | 31 | 6.70 | -18.00 | -16.00 |
|  | M2727 | ICSBP Q6 | 27 | 5.80 | -14.00 | -12.00 |
|  | M533 | STTTCRNTTT IRF Q6 | 23 | 5.00 | -13.00 | -11.00 |
|  | M30054 | MAML1 TARGET GENES | 27 | 5.80 | -12.00 | -10.00 |
|  | M14066 | IRF Q6 | 24 | 5.20 | -12.00 | -10.00 |
|  | M15616 | RGAGGAARY PU1 Q6 | 34 | 7.30 | -12.00 | -9.90 |
|  | M9412 | RTTTNNNYTGGM UNKNOWN | 16 | 5.00 | -11.00 | -9.00 |
|  | M2709 | IRF1 01 | 23 | 5.00 | -11.00 | -8.90 |
|  | M12520 | SOX9 B1 | 19 | 5.90 | -11.00 | -8.90 |
|  | M30200 | TERF1 TARGET GENES | 24 | 5.20 | -10.00 | -8.50 |
|  | M9902 | ELF1 Q6 | 21 | 4.50 | -9.50 | -7.60 |
|  | M12258 | IRF7 01 | 21 | 4.50 | -9.20 | -7.30 |
|  | M4770 | COREBINDINGFACTOR Q6 | 21 | 4.50 | -8.60 | -6.80 |
|  | M18230 | ETS Q4 | 19 | 4.10 | -7.80 | -6.00 |
|  | M572 | TGCCAAR NF1 Q6 | 27 | 8.40 | -7.60 | -5.90 |
|  | M14376 | PU1 Q6 | 18 | 3.90 | -7.50 | -5.80 |
|  | M9034 | OCT1 04 | 15 | 4.70 | -7.20 | -5.50 |
|  | M19265 | SREBP1 Q6 | 15 | 4.70 | -7.10 | -5.40 |
|  | M17687 | IRF2 01 | 13 | 2.80 | -7.00 | -5.30 |
|  | M3147 | PAX4 03 | 15 | 4.70 | -6.90 | -5.20 |

Figure 9. Summary of enrichment analysis in Cell Type Signatures.

| \_PATTERN\_ | GO | Description | Count | % | Log10(P) | Log10(q) |
| --- | --- | --- | --- | --- | --- | --- |
|  | M39160 | GAO LARGE INTESTINE 24W C11 PANETH LIKE CELL | 103 | 22.00 | -100.00 | -96.00 |
|  | M39305 | CUI DEVELOPING HEART C8 MACROPHAGE | 94 | 20.00 | -100.00 | -96.00 |
|  | M40157 | DESCARTES FETAL CEREBELLUM MICROGLIA | 129 | 28.00 | -100.00 | -96.00 |
|  | M39051 | MANNO MIDBRAIN NEUROTYPES HMGL | 114 | 25.00 | -93.00 | -89.00 |
|  | M39266 | HU FETAL RETINA MICROGLIA | 94 | 20.00 | -85.00 | -82.00 |
|  | M39166 | GAO LARGE INTESTINE ADULT CI MESENCHYMAL CELLS | 92 | 20.00 | -85.00 | -81.00 |
|  | M39306 | CUI DEVELOPING HEART C9 B T CELL | 68 | 15.00 | -81.00 | -78.00 |
|  | M39077 | ZHONG PFC MAJOR TYPES MICROGLIA | 95 | 21.00 | -80.00 | -77.00 |
|  | M39022 | FAN EMBRYONIC CTX BIG GROUPS MICROGLIA | 89 | 19.00 | -79.00 | -76.00 |
|  | M40210 | DESCARTES FETAL INTESTINE MYELOID CELLS | 71 | 15.00 | -72.00 | -69.00 |
|  | M40168 | DESCARTES FETAL CEREBRUM MICROGLIA | 93 | 20.00 | -72.00 | -69.00 |
|  | M39110 | AIZARANI LIVER C6 KUPFFER CELLS 2 | 66 | 14.00 | -64.00 | -61.00 |
|  | M39106 | AIZARANI LIVER C2 KUPFFER CELLS 1 | 63 | 14.00 | -64.00 | -61.00 |
|  | M39109 | AIZARANI LIVER C5 NK NKT CELLS 3 | 54 | 12.00 | -64.00 | -60.00 |
|  | M39105 | AIZARANI LIVER C1 NK NKT CELLS 1 | 56 | 12.00 | -60.00 | -57.00 |
|  | M40314 | DESCARTES FETAL THYMUS ANTIGEN PRESENTING CELLS | 57 | 12.00 | -59.00 | -56.00 |
|  | M40264 | DESCARTES FETAL PANCREAS MYELOID CELLS | 63 | 14.00 | -59.00 | -56.00 |
|  | M40154 | DESCARTES FETAL ADRENAL LYMPHOID CELLS | 55 | 12.00 | -58.00 | -55.00 |
|  | M39021 | FAN EMBRYONIC CTX BIG GROUPS BRAIN IMMUNE | 53 | 11.00 | -57.00 | -54.00 |
|  | M41715 | FAN OVARY CL13 MONOCYTE MACROPHAGE | 77 | 17.00 | -56.00 | -53.00 |

Figure 10. Summary of enrichment analysis in DisGeNET^15^.

| \_PATTERN\_ | GO | Description | Count | % | Log10(P) | Log10(q) |
| --- | --- | --- | --- | --- | --- | --- |
|  | C3714514 | Infection | 71 | 15.00 | -47.00 | -44.00 |
|  | C0024138 | Lupus Erythematosus, Discoid | 72 | 16.00 | -44.00 | -42.00 |
|  | C0024131 | Lupus Vulgaris | 67 | 14.00 | -40.00 | -38.00 |
|  | C0004623 | Bacterial Infections | 71 | 15.00 | -40.00 | -37.00 |
|  | C0409974 | Lupus Erythematosus | 68 | 15.00 | -40.00 | -37.00 |
|  | C0018133 | Graft-vs-Host Disease | 60 | 13.00 | -38.00 | -35.00 |
|  | C2363741 | HIV-1 infection | 72 | 16.00 | -37.00 | -35.00 |
|  | C0524910 | Hepatitis C, Chronic | 55 | 12.00 | -33.00 | -31.00 |
|  | C0036202 | Sarcoidosis | 53 | 11.00 | -32.00 | -30.00 |
|  | C0524909 | Hepatitis B, Chronic | 53 | 11.00 | -32.00 | -29.00 |
|  | C4048329 | Immunosuppression | 63 | 14.00 | -32.00 | -29.00 |
|  | C0007570 | Celiac Disease | 58 | 13.00 | -31.00 | -29.00 |
|  | C0021368 | Inflammation | 55 | 12.00 | -31.00 | -29.00 |
|  | C0867389 | Chronic graft-versus-host disease | 42 | 9.10 | -31.00 | -28.00 |
|  | C0011633 | Dermatomyositis | 41 | 8.90 | -30.00 | -28.00 |
|  | C0011615 | Dermatitis, Atopic | 66 | 14.00 | -30.00 | -27.00 |
|  | C0017661 | IGA Glomerulonephritis | 53 | 11.00 | -30.00 | -27.00 |
|  | C0024143 | Lupus Nephritis | 55 | 12.00 | -30.00 | -27.00 |
|  | C0042384 | Vasculitis | 44 | 9.50 | -30.00 | -27.00 |
|  | C0019348 | Herpes Simplex Infections | 61 | 13.00 | -29.00 | -27.00 |

Figure 11. Summary of enrichment analysis in PaGenBase^16^.

| \_PATTERN\_ | GO | Description | Count | % | Log10(P) | Log10(q) |
| --- | --- | --- | --- | --- | --- | --- |
|  | PGB:00011 | Tissue-specific: spleen | 103 | 22.00 | -85.00 | -82.00 |
|  | PGB:00041 | Tissue-specific: Blood | 69 | 15.00 | -67.00 | -64.00 |
|  | PGB:00016 | Tissue-specific: thymus | 41 | 8.90 | -32.00 | -30.00 |
|  | PGB:00017 | Tissue-specific: skin | 30 | 9.30 | -24.00 | -22.00 |
|  | PGB:00048 | Tissue-specific: bone marrow | 34 | 7.30 | -23.00 | -21.00 |
|  | PGB:00086 | Cell-specific: Lymphoma burkitts Daudi | 13 | 2.80 | -13.00 | -11.00 |
|  | PGB:00081 | Cell-specific: Bronchial Epithelial Cells | 14 | 4.40 | -9.60 | -7.70 |
|  | PGB:00018 | Tissue-specific: lung | 29 | 6.30 | -8.90 | -7.10 |
|  | PGB:00125 | Cell-specific: RS11846 | 6 | 1.30 | -7.70 | -6.00 |
|  | PGB:00005 | Tissue-specific: tonsil | 10 | 2.20 | -7.60 | -5.90 |
|  | PGB:00064 | Cell-specific: RPMI 8226 | 8 | 1.70 | -7.50 | -5.80 |
|  | PGB:00131 | Cell-specific: B-lymphocyte | 8 | 1.70 | -6.40 | -4.80 |
|  | PGB:00043 | Cell-specific: CD56+ NKCells | 9 | 1.90 | -6.20 | -4.60 |
|  | PGB:00156 | Tissue-specific: Tongue | 7 | 1.50 | -6.10 | -4.40 |
|  | PGB:00036 | Cell-specific: MOLT4 | 12 | 2.60 | -5.80 | -4.20 |
|  | PGB:00053 | Cell-specific: NHEK | 20 | 4.30 | -5.70 | -4.10 |
|  | PGB:00085 | Cell-specific: RL7 | 6 | 1.30 | -5.40 | -3.90 |
|  | PGB:00184 | Cell-specific: SR | 6 | 1.30 | -5.00 | -3.50 |
|  | PGB:00082 | Cell-specific: Breast cell | 6 | 1.90 | -4.60 | -3.10 |
|  | PGB:00010 | Tissue-specific: adipose tissue | 9 | 2.80 | -4.00 | -2.60 |

### Reference

1. Zhou et al., Metascape provides a biologist-oriented resource for the analysis of systems-level datasets. Nature Communications (2019) 10(1):1523.
2. Saldanha AJ. Java Treeview - extensible visualization of microarray data. Bioinformatics (2004) 20:3246-3248
3. Krzywinski M. et al. Circos: an information aesthetic for comparative genomics. Genome Res (2009) 19:1639-1645
4. Zar, J.H. Biostatistical Analysis 1999 4th edn., NJ Prentice Hall, pp. 523
5. Hochberg Y., Benjamini Y. More powerful procedures for multiple significance testing. Statistics in Medicine (1990) 9:811-818.
6. Cohen, J. A coefficient of agreement for nominal scales. Educ. Psychol. Meas. (1960) 20:27-46.
7. Shannon P. et al., Cytoscape: a software environment for integrated models of biomolecular interaction networks. Genome Res (2003) 11:2498-2504.
8. Szklarczyk D. et al. STRING v11: protein-protein association networks with increased coverage, supporting functional discovery in genome-wide experimental datasets. Nucleic Acids Res. (2019) 47:D607-613.
9. Stark C. et al. BioGRID: a general repository for interaction datasets. Nucleic Acids Res. (2006) 34:D535-539.
10. Turei D. et al. A scored human protein-protein interaction network to catalyze genomic interpretation. Nat. Methods. (2016) 13:966-967.
11. Li T. et al. A scored human protein-protein interaction network to catalyze genomic interpretation. Nat. Methods. (2017) 14:61-64.
12. Bader, G.D. et al. An automated method for finding molecular complexes in large protein interaction networks. BMC bioinformatics (2003) 4:2.
13. https://metascape.org/COVID.
14. Subramanian A, et al. Gene set enrichment analysis: A knowledge-based approach for interpreting genome-wide expression profiles. Proc Natl Acad Sci U S A 102, 15545-15550 (2005).
15. Pinero J, et al. DisGeNET: a comprehensive platform integrating information on human disease-associated genes and variants. Nucleic acids research 45, D833-D839 (2017).
16. Pan JB, et al. PaGenBase: a pattern gene database for the global and dynamic understanding of gene function. PLoS One 8, e80747 (2013).
